# Mapping programmes for mental health promotion in Singapore: A scoping review

**DOI:** 10.1371/journal.pone.0347518

**Published:** 2026-04-28

**Authors:** P.V. AshaRani, Xin Er Ong, Zakir Karuvetil, Christine S.M. Chen, Lim Ther, Lee Cheng, Mythily Subramaniam, Timothy Liu

**Affiliations:** 1 Research Division, Institute of Mental Health, Singapore; 2 Office of Population Health Operations, Institute of Mental Health, Singapore; 3 National Addictions Management Service, Institute of Mental Health, Singapore; 4 National University Health System, NUHS Tower Block, Singapore; 5 Operations, Office of Population Health Operations, Institute of Mental Health, Singapore; University of Toronto, CANADA

## Abstract

Mental health disorders are on the rise globally, with policies promoting programmes that aim to enhance mental wellbeing across various population domains. This scoping review aims to examine the literature to identify and map the mental health programmes in Singapore, while identifying critical knowledge gaps and scope for future systematic reviews. A systematic search was conducted across multiple databases including Medline, PsycINFO, CINAHL, OpenGrey and ProQuest, complemented by hand and bibliography searches. Articles published from 2000 onwards in English language were included. Independent parallel reviews were conducted by multiple reviewer pairs. Data was extracted into standardised, pre-piloted templates that incorporated the Template for Intervention Description and Replication (TIDieR) checklist. One hundred and six studies were included in the review, demonstrating diverse programmes targeting different age groups, with particular focus on older adults in hospital and community settings. The programmes addressed mental health outcomes in individuals with mental disorders, physical conditions, neurodevelopmental and learning disabilities, and among healthy individuals. Implemented by trained care teams or multidisciplinary professionals, these programmes generally showed positive outcomes. However, significant gaps were identified in the literature regarding user experiences, with minimal focus on implementation barriers and enablers. Notably, there was limited evidence of successful community-level implementation beyond the experimental phase, raising questions about programme sustainability and real-world effectiveness. Critical gaps were also identified in youth-specific suicide prevention programmes, despite suicide being a leading cause of death among youths, and programmes targeting the impact of built environment on mental health outcomes and workplace wellbeing. These areas represent important opportunities for future research and intervention development in Singapore’s mental health landscape. Singapore has developed an active research network over the past decade to design and implement programmes aimed at improving mental health across different populations and settings. However, stronger collaborative approaches between academics and policymakers are needed to better utilise research findings and understand which programmes add value to the public health domain. Future research should focus on implementation science, long-term sustainability, and cost-effectiveness of these programmes in real-world settings.

## Introduction

Mental health extends beyond the absence of psychological disorders. It represents a state of wellbeing that enables individuals to cope with routine life stressors while maintaining productive functioning [1]. Optimal mental health facilitates better functioning, coping, and thriving [2], whereas individuals with mental health conditions experience varying states along a continuum – from optimal cognitive and emotional functioning to severe psychological distress and disabilities [2].

Global statistics reveal that approximately 970 million people lived with mental health disorders in 2019 [2–3], with 11.63% of individuals aged 5–24 years experiencing at least one mental health condition [4]. The burden of these disorders is substantial, accounting for 31.14 million years lost to disabilities (YLDs) within this age group. In 2019, mental health disorders constituted 15.6% of YLDs globally, with depressive and anxiety disorders ranking second and sixth, respectively, among the leading causes of YLDs worldwide [2]. A recent review spanning 29 countries and 156, 331 adults (aged 18 and above) revealed lifetime prevalence rates of mental health disorders at 28.6% for males and 29.8% for females [5]. The study further demonstrated elevated lifetime morbidity risk at age 75, reaching 46.4% in males and 53.1% in females. Among men, alcohol abuse, major depressive disorder, and substance use disorder were the primary contributors, while women were predominantly affected by major depressive disorder, post-traumatic stress disorder, and generalised anxiety disorders.

A systematic review of 439 articles from 48 countries demonstrated substantial economic implications, with societal costs ranging from USD 547 Purchasing Power Parity (PPP) to 16,783 PPP per capita [6]. The global burden of mental health disorders intensified significantly during the COVID-19 pandemic [2]. The prevalence of major depressive disorder increased from 193 million pre-pandemic cases to 246 million during the pandemic, while anxiety disorders escalated from 298 million to 374 million cases, representing increases of 27.5% and 25.5% respectively. These escalating prevalence rates and associated economic burden underscore the urgent need for evidence-based preventive strategies and management interventions to mitigate both individual suffering and societal costs.

In response to this growing crisis, population-level prevention and health promotion strategies have become essential for enhancing mental wellbeing and resilience. The World Health Organisation’s (WHO) global mental health action plan 2013–2030 emphasises multisectoral collaboration in delivering preventive activities through integrated healthcare systems, promoting mental health among at-risk populations, and ensuring accessible community-level interventions [2]. These efforts focus on enhancing protective factors (including social connections, employment, and physical activity) while reducing risk factors (such as substance use, chronic conditions, and stigma) [7]. However, implementation challenges have resulted in significant treatment gaps, with coverage varying dramatically between high-income nations (33%) and low-middle income countries (8%) [8–9]. The subsequent revision endorsed by the World Health Assembly included additional targets to address these challenges through improved caregiver support, awareness programmes, suicide prevention initiatives, and integration of mental health into primary care which was adopted by various countries worldwide as a framework for national mental health policy development [10]. In Singapore, these global imperatives take on particular significance given the nation’s commitment to building a comprehensive healthcare system that addresses both physical and mental wellbeing within its unique multicultural and urban context.

In Singapore, mental health disorders affect 13.9% of the population [11], imposing an annual incremental societal cost of S$1.7 billion [12]. The post-pandemic landscape has witnessed an escalation in both prevalence and costs, with annual expenditure reaching approximately S$15.7 billion [13]. To address these challenges, Singapore implemented the National Mental Health Blueprint (NMHB, 2010–2012) and subsequent National Mental Health Strategy, which established frameworks for community healthcare integration, enhanced mental health-primary care partnerships, and developed integrated care pathways addressing both mental and physical health needs [14]. The Community Mental Health (CMH) Masterplan further strengthened this initiative by prioritising early intervention and community-based support systems [15]. Recent developments, including workplace mental health initiatives, digital mental health services, and the Healthier SG programme, have reinforced this systematic approach to mental healthcare delivery [16]. Healthier SG shifts the care model from reactive care for those who are sick to prevention of health problems among the population. The plan includes promotion of health beyond clinical care by addressing and learning more about the social, environmental, and behavioural determinants of health. It involves increasing the number of family doctors who can provide preventive care, develop personalised health plans (diet, exercise, regular screening, etc.), and extend activities through community partners. This will facilitate onboarding of the population to the Healthier SG initiatives to ensure a holistic approach to care, and reinforce the enablers (e.g., IT systems, manpower, and regional health managers). To date, no reviews were conducted locally to map the literature landscape that is needed for understanding the reach and impact of these programmes.

Given the escalating prevalence and associated costs, understanding effective preventive and management strategies for mental health disorders is crucial. Despite Singapore’s strategic policy framework and substantial investment in mental health initiatives, no systematic mapping of implemented programmes and their outcomes has been conducted to date. While international reviews have examined mental health interventions in various contexts [17], none have specifically focused on Singapore’s unique healthcare system, multicultural population, and policy environment. This represents a critical knowledge gap, as the effectiveness of mental health programmes can vary significantly across different healthcare systems, cultural contexts, and implementation settings. Without a comprehensive understanding of what programmes have been implemented locally, their target populations, theoretical foundations, and outcomes, policymakers and practitioners lack essential evidence to guide resource allocation and programme development. There is a dearth of literature on the interventions or programmes that promote the mental health outcome of the population locally. This knowledge synthesis is vital for identifying globally applicable interventions to mitigate the progression of the mental health crisis. The scoping review design was chosen due to the broad nature of the research question, which aimed to map the available evidence on mental health programmes in Singapore across diverse populations, settings, and intervention types. This approach was particularly appropriate given the uncertainty about whether sufficient data existed for a systematic review, and the need to first understand the breadth rather than the depth of available evidence.

This scoping review aimed to identify and map mental health programmes and interventions (concept) implemented across Singapore’s healthcare and community settings (context) targeting all residents (population), following the Population, Context, Concept (PCC) framework. The specific research questions were:

a) what are the available mental health programmes in Singapore, b) what are the key characteristics of these programmes (e.g., target populations, setting, delivery approaches, outcomes and direction of evidence) c) what are the current knowledge gaps, and areas warranting systematic review or future research. By collating and summarising the available evidence, this review provides insights into mental health promotion strategies within Singapore’s urban Asian context, including how these initiatives operate within existing systems and sociocultural frameworks. The findings may be particularly relevant for other urban Asian settings with similar sociocultural characteristics and healthcare structures seeking to enhance their mental health promotion strategies.

## Materials and methods

This scoping review adopted Joanna Briggs Institute’s (JBI) guidance for scoping reviews [18]. The protocol for the scoping review has been published in Open Science Framework repository [19]. The protocol followed JBI best practice guidance and reporting items for scoping review protocol [20]. The PRISMA ScR fillable checklist was used to track the reported item [21].

### Search strategy

A systematic search was conducted in Medline, PsycINFO, CINAHL, OpenGrey and ProQuest. Hand searches were conducted in the Singapore Medical Journal and the Annals of the Academy of Medical Science Singapore. Local grey literature was searched through additional hand searches at government and other stakeholder websites. Bibliographic searches were performed on the included articles to identify any missing articles. The search was restricted to articles published in English between 2000 to the search date. This timeframe was chosen as the majority of Singapore’s mental health policies were implemented after 2000. The focus on English language publications was appropriate as this is the primary language of academic and scientific publication in Singapore. Only studies involving human participants were included given the public health focus of the review. The search strategy and keywords were developed through discussion with the mental health team which included healthcare system administrators, clinicians, population health specialists, experts in evidence synthesis, as well as through literature searches. The preliminary keywords were developed with PCC components and included indexed/MESH terms, keywords, proximity operators, and phrases. Pilot searches were conducted in Medline, and the search terms were modified through discussion among team members. The sensitivity and specificity of the searches were assessed by screening the search outputs against pre-identified articles and by reviewing the initial 100 articles to refine the keywords. The final set of keywords were approved by all team members before the final search in all the included databases. The detailed list of keywords used is included in the supplementary file (**S2 Appendix**). The initial search was conducted on 24 August 2023 and a search update on 15 May 2025.

### Inclusion and exclusion criteria

Studies were included if they involved programmes conducted in humans, looking at programmes targeting mental health outcomes, conducted in Singapore, in English and published from 2000 onwards. Articles were excluded if they were case reports or series or conference proceedings, reviews, studies looking at physical health outcomes, and those conducted outside Singapore or not presenting separate data for multicountry studies. Mental health programmes were defined as structured and planned sets of activities or interventions intended to improve the mental health outcomes of targeted populations, beyond routine clinical care.

### Screening and study selection

References were imported, sorted, deduplicated and managed in EndNote. Screening included title and abstract and full text screening. All screening was conducted by reviewer pairs as independent parallel reviews. For title and abstract screening, a screening template was created in Excel with clear definition of the inclusion and exclusion criteria. Articles with uncertain eligibility during initial screening were retained for full-text review. The reviewers underwent training and conducted a pilot test using 20 articles to ensure consistency in the screening process across individuals and pairs. An agreement of 80% or above was used as a benchmark. Disagreements below this threshold were investigated and the definitions of inclusion and exclusion criteria were revised for clarity and the changes were communicated to the reviewers by the lead author. Upon reaching acceptable agreement level, the screening (independent and parallel) was continued for the rest of the articles. Any discrepancies were discussed and resolved, and advice was sought from a senior reviewer when needed. The screening templates captured the audit trails for all discussions, screening decisions and reasons for exclusion of articles. Where the information was insufficient to determine the inclusion of the articles, the corresponding author of the article was contacted for clarification. Three attempts were made before the article was excluded. A similar strategy was employed for full text reviews of the included articles.

### Data extraction

A data extraction template was developed based on the aims, PCC components, TIDieR checklist, and other data fields recommended by JBI [18]. The fields captured were author, year, source of publication, population, sociodemographic characteristics of the population. Additional fields included sample size, study settings, sampling method, type and description of the programme, duration of the programme, study design, discipline, treatment and comparator arms (for interventional studies), institution, outcomes, tools used to measure outcomes, main findings, direction of evidence. The template was piloted using 5 articles and discrepancies were noted and resolved through discussion. A standardised definition guide was developed for each item in the data extraction template to ensure consistent interpretation and extraction across all reviewers. This guide provided explicit operational definitions and criteria for data extraction. Data extraction was conducted in pairs independently and checked by AR and XE. Since scoping reviews typically do not include risk of bias assessments (RoB) [20], the current review did not include RoB assessments.

### Data analysis

The extracted data were analysed descriptively and synthesised narratively in accordance with the scoping review methodology. No meta-analysis or RoB assessments were conducted due to the scoping review methodology which does not include inferential statistics. Missing information was recorded and marked as “not reported” for transparency, and overlapping categories were reported separately. This information is reflected in the data summary and in the tables. Programme categories were derived through discussion within the team that followed an inductive thematic analysis. This included review of programmes, components and descriptions to generate initial codes, which were then refined and regrouped based on programme characteristics. AR and XE checked all entries from individual coders and conflicts were resolved through discussion or a third reviewer (MS) where needed. Quantitative analysis was performed using Microsoft Excel and Statistical Package for the Social Sciences (SPSS) version 23 (IBM SPSS Statistical Package). Results were summarised and presented through tables, Figures, and graphs to facilitate clear visualisation of patterns and trends in the data. The narrative synthesis followed a framework approach, organising findings by programme categories and characteristics to address the research objectives.

## Results

**Fig 1:**
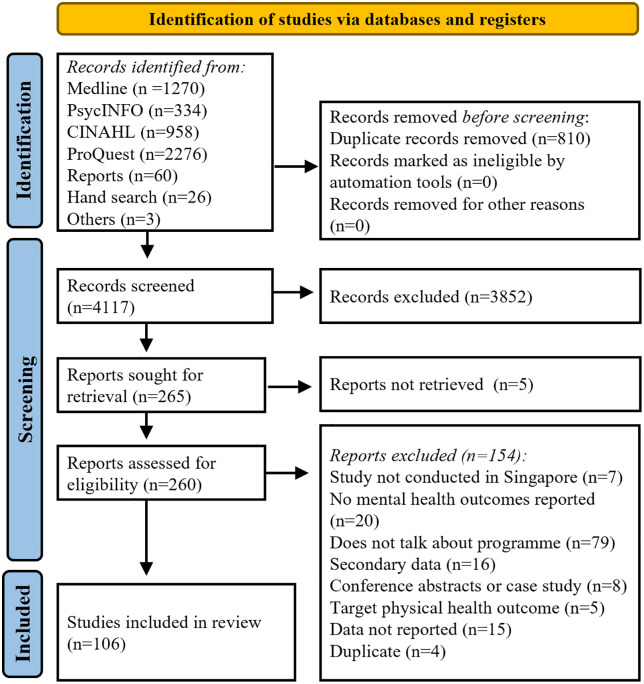
PRISMA flow chart: The flowchart shows the article screening and selection.

Adherence to JBI guidance was documented using Preferred Reporting Items for Systematic Reviews and Meta-Analyses extension for Scoping Reviews (PRISMA-ScR, **S1 Table**). Fig 1 shows the PRISMA flowchart that details the search results and screening.

### Study characteristics and overview of programmes

**Fig 2:**
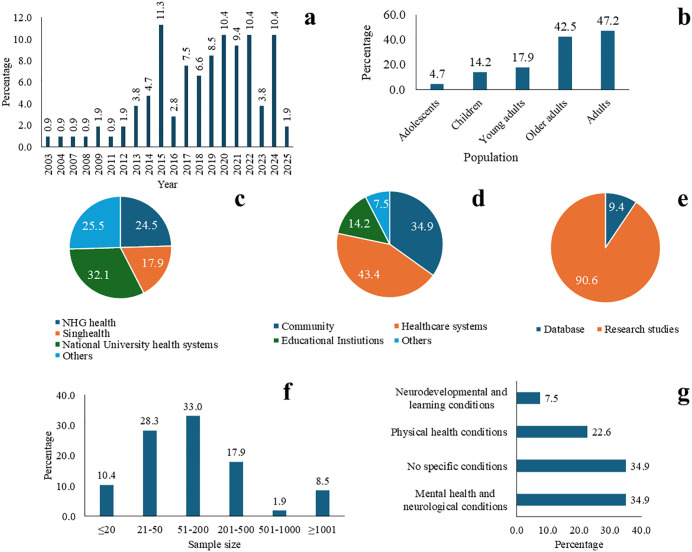
Distribution of key study characteristics across articles. **(a)** Publication frequency, **(b)** Population, **(c)** Institutions that hosted the study, **(d)** Study settings, **(e)** Data sources, **(f)** Sample sizes for included studies, **(g)** Health conditions under study.

A total of 106 articles were included for data extraction. Publication frequency showed a notable increase from 2013 (Fig 2a), with peak publication numbers occurring in 2015 (n = 12, 11.3%), 2020 (n = 11, 10.4%), and 2022 and 2024 (n = 11, 10.4% each). Fifteen studies included children (14.2%), five included adolescents (4.7%), 19 included young adults (17.9%), 50 included adults (47.2%), and 45 included older adults (42.5%, Fig 2b) with 27 studies (25.5%) having a combination of different age groups that were counted in the respective categories.

The institutional contributions (for publications) were distributed across three major healthcare clusters and other institutions. The National University Health System (NUHS) was the leading contributor with 34 publications (32.1%, Fig 2c). This included National University of Singapore (NUS) (n = 26, 24.5%), National University Health System/National University Hospital (NUHS/NUH) (n = 4, 3.8%), Jurong Health Campus (JHC) including Ng Teng Fong General Hospital (NTFGH) (n = 3, 2.8%) and NUHS polyclinic (n = 1, 0.9%). The National Healthcare Group (NHG health) contributed 26 publications (24.5%), with Institute of Mental Health (IMH) being the major contributor (n = 20, 18.9%), followed by Tan Tock Seng Hospital (TTSH) (n = 4, 3.8%), Khoo Teck Puat Hospital (KTPH) and National Skin Centre (NSC) contributing 1 each (0.9%).

The SingHealth cluster contributed 19 publications (17.9%), distributed across KK Women’s and Children’s Hospital (KKH) (n = 6, 5.7%), Singapore General Hospital (SGH) (n = 6, 5.7%), Changi General Hospital (CGH) (n = 4, 3.8%), National Cancer Centre Singapore (NCCS) (n = 2, 1.9%), and SingHealth Polyclinics (n = 1, 0.9%). The remaining 27 publications (25.2%) came from other institutions, with Duke-NUS Medical School being the main contributor (n = 7, 6.5%), followed by Nanyang Technological University (NTU; n = 7, 6.5%), Dyslexia Association of Singapore (DAS) and Ministry of Social and Family Development (MSF) (n = 2 each, 1.9%), and various other institutions contributing one publication each.

Study participants were primarily recruited from healthcare (n = 46, 43.4%), community settings (n = 37, 34.9%; Fig 2d) and educational institutions (n = 15, 14.2%). Eight studies (7.5%) recruited participants from other sources such as prison, nursing homes, children’s home, dyslexia association, hospices and online platforms. The studies utilised both primary data collection (n = 96, 90.6%) and existing databases (n = 10, 9.4%), including clinical databases (Fig 2e).

Sample sizes varied considerably across the 106 articles, with the largest group of studies (n = 35, 33%) having sample sizes between 51–200 participants (Fig 2f). Eleven studies (10.4%) exclusively recruited female participants, and one (0.9%) recruited only male participants, while gender-specific data was not reported in 12 studies (11.3%). Study designs included Randomized Controlled Trials (RCT; n = 25, 23.6%), quasi-experimental studies (n = 18, 17.0%), other interventional studies (n = 34, 32.1%), cohort studies (n = 7, 6.6%), other observational studies (n = 8, 7.5%), mixed methods studies (n = 7, 6.6%) and qualitative studies (n = 7, 6.6%). Of these, 12 (11.3%) were pilot studies across different study designs.

The mental health programmes were categorised into four main types based on their focus area (Fig 2g): programmes for those with mental health and neurological conditions (n = 37, 34.9%), those addressing mental health in those with physical health conditions (n = 24, 22.6%), programmes for those with neurodevelopmental and learning conditions (n = 8, 7.5%), and programmes for healthy populations without any specific diseases (n = 37, 34.9%). The intervention approaches in these programmes included seven categories: therapeutic interventions (n = 15, 14.2%), educational and psychoeducational interventions (n = 30, 28.3%), management and treatment programmes (n = 27, 25.5%), prevention and early intervention initiatives (n = 25, 23.6%), psychosocial support services (n = 1, 0.9%), physical and activity-based programmes (n = 4, 3.8%), and other programmes (n = 4, 3.8%). The majority of programmes (96 out of 106, 90.6%) demonstrated positive outcomes across various mental health domains, with strong representation from Singapore’s three major healthcare clusters and academic institutions. Programmes addressed diverse age groups with a focus on older adults, had strong theoretical foundations, and were implemented across settings through diverse delivery modes. However, significant gaps were identified in youth-focused interventions, workplace mental health programmes, and community-based sustainability of research findings. The detailed profiles of the studies is indicated in S2 Table. The following sections examine these four categories in great detail.

### Programmes for mental health and neurological conditions

#### Types of programmes.

Among the 37 articles under this category (Table 1), there were 35 peer-reviewed articles (94.6%), one practice forum and one thesis (2.7% each), with 8 articles (21.6%) using existing databases as data sources and the rest involving primary research (78.4%).

**Table 1:** Study characteristics for those targeting mental health outcomes in those with mental health and neurological conditions.

Author	Year	Institution	Population	Setting	Gender	Intervention/ Programme	Focus of the programme	Sample Size	Mental Health Outcomes	Measures	Direction
Aloweni et al., 2022 [22]	2022	SGH	Adults	Hospital	Male = 13 Female = 31	Mindfulness Therapy	Caregiver stress and anxiety	N = 44	1. Stress 2. Anxiety 3. QOL 4. Caregiver response	1. PSS 2. STAI 3. SF-36, KDQOL-SF 4. CRA	Positive outcome
Chan et al., 2013 [23]	2013	NUS	Older adults	Community	Male = 5 Female = 21	Life story book	Depression	N = 26	1. Depression	1. GDS-15	Positive outcome
Chan et al., 2014 [24]	2014	NUS	Older adults	Community	Male = 6 Female = 23	Lifestory reviews	Depression	N = 29	1. Depression	1. GDS-15	Positive outcome
Chew et al., 2025 [25]	2025	TTSH	Older adults	Community	Male = 35 Female = 115	ADL+	Cognitive decline	N = 150	1. Cognitive function 2. Cognitive decline 3. Depressive symptoms 4. QOL 5. Attention 6. Processing speed 7. Memory 8. Executive function	1. mCMMSE 2. AD8 3. GDS 4. EQ-5D-5L, EQ VAS 5. CTT 1 6. Symbol Search, SDMT 7. Logical Memory sub-test from the WMS-IV 8. CTT 2, animal category	Positive outcome
Huang et al., 2017 [26]	2017	IMH	Adults, older adults	Community	Male = 135 Female = 386	General Practitioner Partnership Programme	Mental disorders	N = 521	1. Referral acceptance rate 2. Readmission rate	1. Referral acceptance rate 2. Readmission rate	Positive outcome
Jiao et al., 2019 [27]	2019	NUS	Adults	Community	Female = 204	Postnatal psychoeducational intervention for first-time mothers	PND	N = 204	1. Maternal parental self-Efficacy 2. Social support 3. Psychological wellbeing	1. PMPSE 2. PICSS-modified 3. EPDS & HADS-A	Positive outcome
Kasmani et al., 2018 [28]	2018	IMH	Young adults, adults, older adults	Hospital and Community	NR	Multilevel Bidirectional Care Coordination Model	Mental disorders	N = 1065	Acceptance rates for referrals to CBPRFs, rejection rates	Annual average acceptance and rejection rates for referrals to CBPRFs	Positive outcome
Khine et al., 2020 [29]	2020	NUS	Older adults	Community	Male = 33 Female = 90	Mindful Awareness Programme	Cognitive function	N = 123	1. Cognitive function 2. Psychological wellbeing	1. Cognitive Function: (a) CDR (b) RAVLT (c) DGS (d) BDT (e) CTT (f) Semantic Fluency Span 2. Psychological wellbeing: (a) GDS (b) GAI	Positive outcome
Klainin-Yobas et al., 2016 [30]	2016	NUS	Adults	Hospital	Male = 21 Female = 34	S-Manage Programme	Stress management	N = 55	1. Objective stress 2. Subjective stress 3. Psychological health	1. Skin temperature, SIgA 2. PSS 3. GHQ	Positive outcome
Koh et al., 2020 [31]	2020	CGH	Older adults	Community	Male = 13 Female = 22	Person-centred creative dance intervention	Dementia care	N = 35	1. Wellbeing 2. QOL 3. Caregiver stress	1. DCM 2. QOL-AD 3. ZBI	Positive outcome
Lai et al., 2019 [32]	2019	NUP	Older adults	Primary Care (Polyclinic)	First visit Male = 90 Female = 176 Review visit NR	Memory Clinic	Dementia care	N = 489	1. Management of dementia cases 2. Presence of behavioral and psychological symptoms of dementia 3. Caregiver needs	1. Percentage of cases managed in primary care 2. Percentage with behavioral/psychological symptoms 3. Percentage of caregivers needing support	Positive outcome
Lee at al., 2016 [33]	2016	KKH	Adults	Hospital	Female = 5245	Postnatal depression screening and early intervention programme	PND	N = 5245 screened N = 307 received intervention	1. Depression 2. Functioning 3. HrQOL	1. EPDS 2. GAF 3. EQ VAS	Positive outcome
Lee et al., 2003 [34]	2003	IMH	Adolescents	Hospital	Male = 1372 Female = 1164	Child Guidance Clinic	Mental disorders	N = 2536	1. Utilisation of mental health services	1. Number of new cases, referral sources	Neutral outcome
Lee et al., 2024 [35]	2024	NUS	Young adults	University	Male = 52 Female = 171 Nonbinary = 1 Other = 1	Intellect	Subclinicial OCD	N = 225	1. OCD symptom severity 2. Psychological distress 3. Perfectionism	1. OCI-R 2. DASS-21 3. FMPS	Positive outcome
Loh et al., 2023 [36]	2023	KKH	Adults	Hospital	Females = 25	Sure-mums intervention	PND and postnatal anxiety	N = 25	1. Mother and baby bonding 2. Depression 3. Functioning	1. PBQ 2. EPDS 3. GAF	Positive outcome
Low et al., 2013 [37]	2013	IMH	Young adults, Adults, older adults	Hospital	NR	Assertive Community Treatment (ACT)	Severe mental illness	N = 155	1. Number of admissions 2. Total number of hospital stay pre and post assertive community management	Medical record review	Positive outcome
Ng et al., 2020 [38]	2020	NUS	Older adults	Community	Male = 149 Female = 185	Community-Based Early Psychiatric Interventional Strategy	Depression	N = 334	1. Receipt of treatment and care 2. Depression 3. QOL	1. Reports from GPs 2. SCID-I, GDS, HDRS, BDI 3. SF-12	Positive outcome
Nyunt et al., 2009 [39]	2009	NUS	Older adults	Community	Male = 1922 Female = 2711	Community-Based Early Psychiatric Interventional Strategy	Depression	N = 4633	1. Depressive symptoms 2.Treatment acceptance 3. Any psychiatric disorder 4. Mental health status	1. GDS 2. Primary care self-report 3. SCID 4. Self-report	Positive outcome
Ong et al., 2019 [40]	2019	IMH	Children	Hospital	Male = 49 Female = 23	Mobile app: RegnaTales	Aggression	N = 72	1. Aggression	1. RPQ	Positive outcome
Pat-Horenczyk et al., 2015 [41]	2015	MSF	Children	Childrens’ Home	Male = 33 Female = 40	Building Emotion and Affect Regulation (BEAR) programme	Emotional dysregulation	N = 73	1. Coping 2. Distress	1-2. Self-report	Positive outcome
Rawtaer et al., 2015 [42]	2015	NUH	Older adults	Community	Male = 25 Female = 76	Pychosocial intervention (Tai Chi exercise, Art Therapy, Mindfulness Awareness Practice and Music Reminiscence Therapy)	Subsyndromal depression and anxiety	N = 101	1. Depression 2. Cognition 3. Anxiety	1. GDS, SDS 2. MMSE 3. GAI, SAS	Positive outcome
Saxena et al., 2018 [43]	2018	NHG	Older adults	Primary Care (Polyclinic)	Male = 91 Female = 172	Primary Care Dementia Clinic	Dementia care	N = 263	1. QOL 2. Caregiver burden	1. QOL-AD, EQ-5D-5L 2. ZBI	Positive outcome
Shah et al., 2015 [44]	2015	IMH	Adults, older adults	Hospital	Male = 6 Female = 16	VR-based stress management (VR DE-STRESS) programme	Stress management	N = 22	1. Stress 2. Depression 3. Anxiety 4. Perceived relaxation 5. Knowledge on stress and stress management	1-3. DASS-21 4. PRS 5. KSSMQ	Positive outcome
Shorey et al., 2013 [45]	2013	NUS	Adults	Community	Female = 122	Postnatal pychoeducation programme	PND	N = 122	1. Maternal parental self-efficacy 2. Social support 3. PND	1. PMPSE 2. PICSS 3. EPDS	Positive outcome
Shorey et al., 2019 [46]	2019	NUS	Adults	Community	Female = 138	Technology-based peer-support intervention programme	PND	N = 138	1. PND 2. Postnatal anxiety 3. Loneliness 4. Perceived social support	1. EDPS, PHQ-9 2. STAI 3. UCLA Loneliness Scale 4. PSSP	Positive outcome
Shorey et al., 2021 [47]	2021	NUS	Older adults	Community	Male = 9 Female = 19	Where-there-is-no-psychiatrist Integrated Personal Therapy (WIPT)	Subsyndromal depression and/or SS anxiety	N = 28	1. Depression 2. Anxiety 3. Life satisfaction 4. Social connectedness (friendship) 5. QOL	1. GDS 2. GAI 3. SWLS 4. FS 5. WHO-QoL-Old	No effect
Sim et al., 2007 [48]	2007	IMH	Young adults and adults	Hospital	Male = 153 Female = 125	Early Psychosis Intervention Programme (EPIP)	Early psychosis	N = 278	1. Severity of psychopathology 2. Level of insight 3. Functioning 4. QOL	1. PANSS 2. SUMD 3. GAF 4. WHOQOL-BREF	Postive outcome
Sim et al., 2021 [49]	2021	NUS	Adolescents, adults	Community	Adolescents Male = 10 Adults Male = 2 Female = 8	Gaming disorder intervention	Gaming disorder	N = 21	1. Changes in gaming habits	1. Qualitative interviews	Positive Outcome
Tan et al., 2015 [50]	2015	IMH	NR	Prison	Male = 50	Psychiatric Housing Unit programme	Life skills	N = 50	1. Engagement 2. Initiative 3. Concentration 4. Interpersonal skills 5. Communication	1-5. Task Behavioural Scale	Positive outcome
Tan et al., 2017 [51]	2017	IMH	Adults, older adults	Community	Male = 19 Female = 31	Illness management and recovery programme	Mental disorders	N = 50	1. Illness management and recovery 2. Psychotic symptoms 3. Functioning	1. IMRS, number of admissions, length of stay 2. BRPS 3. GAS	Positive outcome
Tan et al., 2021 [52]	2021	NUH	Adults	Hospital	Male = 16 Female = 24	Virtual screen-based stress management programme (V-DESSERTS)	Stress management	N = 40	1. Subjective stress 2. Objective stress 3. Relaxation levels 4. Knowledge on stress and medication management	1. PSS, NSRS 2. Blood pressure, heart rate, skin temperature 3. PRS 4. KSMMQ	Positive outcome
Tan et al., 2022 [53]	2022	Dementia Singapore Ltd	Older Adults	Community	Male = 6 Female = 15	Arts and Dementia Programme	Wellbeing	N = 21	1. Behavioural tendencies 2. Mood and engagement	1-2. DCM	Positive outcome
Teo et al., 2021 [54]	2021	CGH	Adults, older adults	Primary Care	Male = 75 Female = 152	Health Wellness Programme	Mental disorders	N = 228	1. Genetic health status 2. Depression 3. Functioning	1. EQ VAS 2. PHQ-9 3. Sheehan Disability Scale	Positive outcome
Verma et al., 2021 [55]	2012	IMH	Young adults, Adults	Hospital	Male = 404 Female = 391	EPIP	Early psychosis	N = 795	1. Psychopathology 2. Functioning	1. PANSS 2. GAF	Positive outcome
Xie et al., 2015 [56]	2015	IMH	Children, adolescents	School	NR	Response, Early Intervention and Assessment in Community Mental Health (REACH)	Mental health	N = 1500	1. Behavioural and emotional problems 2. Severity of illness 3. Functioning	1. SDQ 2. CGI 3. CGAS	Positive outcome
Yeo and Choi, 2011 [57]	2011	NTU	Children	School	Male = 82 Female = 13	Cognitive-behavioural therapy	Disruptive behaviour	N = 95	1. Impulsivity and self-control 2. Classroom and home behaviour 3. Self-esteem	1. Teacher self-control rating scale 2. Teacher rating scale, Student rating scales 3. Self-esteem scale	Positive outcome
Zhenru and Jern-Yi 2014 [58]	2014	IMH	Young adults, Adults	Community	NR	Community Mental Health Team (CMHT)	Mental disorders	N = 3652	1. Length of inpatient stay 2. Number of admissions	NR	Positive outcome

SGH: Singapore General Hospital, QOL: Quality of Life, PSS: Perceived Stress Scale, STAI: State-Trait Anxiety Inventory, SF-36: 36-Item Short Form Health Survey, KDQOL: Kidney Disease Quality of Life Short Form, CRA: Caregiver Reaction Assessment, NUS: National University of Singapore, GDS-15: Geriatric Depression Scale-15, ADL + : Activities of Daily Living + , TTSH: Tan Tock Seng Hospital, mCMMSE: modified Chinese Mini-Mental State Examination, AD8: Eight-item Informant Interview to Differentiate Aging and Dementia, GDS: Geriatric Depression Scale, EQ-5D-5L: European Quality of Life (EuroQOL) 5 Dimensions 5 Level questionnaire, EQ VAS: EuroQOL Visual Analogue Scale, CTT: Colour Trails Test, SDMT: Symbol Digit Modalities Test, WMS-IV: Wechsler Memory Scales-4th Edition, IMH: Institute of Mental Health, PND: Postnatal Depression, PMPSE: Perceived Maternal Parental Self-efficacy tool, PICSS-modified: Perinatal Infant Care Social Support, EPDS: Edinburgh Postnatal Depression Scale, HADS-A: Hospital Anxiety and Depression Scale – Anxiety subscale, CBPRF: Community-based Residential Psychiatric Rehabilitation Facilities, CDR: Clinical Dementia Rating, RAVLT: Rey Auditory Verbal Learning Test, DGS: Digit Span Test, BDT: Block Design Test, GAI: Geriatric Anxiety Inventory, SIgA: Salivary Immunoglobulin A, GHQ: General Health Questionnaire, CGH: Changi General Hospital, DCM: Dementia Care Mapping, QOL-AD: Quality of Life in Alzheimers’ Disease, ZBI: Zarit Burden Interview, NUP: National University Polyclinics, KKH: KK Women’s and Children’s Hospital, HrQOL: Health-related Quality of Life, GAF: Global Assessment of Functioning, OCD: Obsessive Compulsive Disorder, OCI-R: Obsessive Compulsive Inventory–Revised, DASS-21: Depression Anxiety Stress Scale-21, FMPS: Frost Multidimensional Perfectionism Scale, PBQ: Postpartum Bonding Questionnaire, SCID-I: Structured Clinical Interview for DSM-IV Axis I Disorders, HDRS: Hamilton Depression Rating Scale, BDI: Beck’s Depression Inventory, SF-12: 12-Item Short Form Health Survey, SCID: Structured Clinical Interview for DSM Disorders, RPQ: Reactive-Proactive Aggression Questionnaire, MSF: Ministry of Social and Family Development, NUH: National University Hospital, SDS: Zung Self-Rating Depression Scale, MMSE: Mini-Mental State Examination, SAS: Zung Self- Rating Anxiety Scale, NHG: National Healthcare Group, PRS: Perceived Relaxation Scale, KSSMQ: Knowledge on Stress and Stress Management Questionnaire, PHQ-9: Patient Health Questionnaire-9, PSSP: Perceived Social Support for Parenting, SWLS: Satisfaction with Life Scale, FS: Friendship Scale, WHO-QoL-Old: World Health Organisation Quality of Life OLD, PANSS: Positive and Negative Scale for Schizophrenia, SUMD: Scale to Assess Unawareness of Mental Disorder, WHOQOL-BREF: World Health Organization Quality of Life-BREF scale, IMRS: Illness Management and Recovery Scale, BRPS: Brief Psychiatric Rating Scale, GAS: Global Assessment Scale, NSRS: Numeric Stress Rating Scale, SDQ: Strengths and Difficulties Questionnaire, CGI: Clinical Global Impression, CGAS: Children’s Global Assessment Scale, NTU: Nanyang Technological University

The programmes demonstrated considerable diversity in their approach and target populations. Management and Treatment interventions were the most prevalent (n = 16, 43.2%), followed by prevention and early interventions (n = 9, 24.3%), educational and psychoeducational interventions (n = 6, 16.2%), therapeutic interventions (n = 5, 13.5%), and psychosocial support (n = 1, 2.7%). These programmes included technology-enhanced programmes featuring mobile applications such as RegnaTales and Intellect alongside virtual reality-based stress management systems. Community-based early intervention programmes included the Community-Based Early Psychiatric Interventional Strategy (CEPIS) and Assertive Community Treatment (ACT). Specialised clinical programmes included memory clinics and primary care dementia clinics, while other programmes included mindfulness-based interventions, and creative arts-based therapies incorporating dance and music reminiscence therapy. Several ongoing hospital-based programmes were also evaluated, including the Early Psychosis Intervention Programme (EPIP). Programmes such as RegnaTales, an app-based intervention for managing aggression in children, showed promising results but were limited to a single pilot study. Further details are indicated in **Table 1**.

### Characteristics of the programmes

#### Population and setting.

A substantial number of programmes (n = 12) focused solely on older adults’ mental health in either hospital (n = 2) or community settings (n = 10), aiming to improve depressive symptoms and to manage cognitive decline. An additional 6 studies included mixed population including adults and older adults. Twelve studies that focused on children, adolescents or young adults were conducted among patient populations (n = 7), students (n = 2), and community residents (n = 3) in various settings. The programmes for adults (n = 15) were mainly conducted in hospital (n = 8) and community (n = 7) settings.

#### Disease specific approaches.

Eleven of the articles (29.7%) were on mood disorders, with 2 of them also focusing on anxiety, 5 (13.5%) on cognitive impairment and dementia and the rest on various mental illnesses. Among the 11 programmes on mood disorders, five focused on post-natal depression; early detection through regular screening, prevention through case management and psychoeducation and management through close follow up at the community level. Six studies focused on depression and/or subsyndromal depression, and included life story reviews, art therapy and collaborative care models. Virtual reality (VR) guided stress management programme was the focus of another study.

#### Population specific approaches.

The programmes for older adults included dementia care programmes, psychosocial interventions (tai chi, art therapy, mindfulness awareness practice, music reminiscence therapy), creative interventions (person-centred dance, arts and dementia program), therapeutic approaches (life-story reviews, integrated personal therapy), community-based strategies (CEPIS), and functional programmes (ADL+). The programmes showed considerable variation across studies. Life story book reviews were evaluated in 2 studies, which were from the same research group. Cognitive stimulation and dementia-focused programmes were examined in 6 studies, with emphasis on preventing and managing dementia in community care models. The programmes targeting children and adolescents focused on behavioural and emotional regulation, including cognitive-behavioural therapy for disruptive behaviours, the Building Emotion and Affect Regulation (BEAR) programme for emotional dysregulation, and the mobile app RegnaTales for aggression management, with delivery primarily in school, hospital, and residential care settings. The programmes for young adults targeted early psychosis intervention, gaming disorder, and subclinical obsessive-compulsive disorder (OCD). The programmes for adults foused on maternal mental health through interventions such as postnatal psychoeducational programmes, the Sure-mums intervention, and technology-based peer-support programmes for postnatal depression and anxiety, alongside comprehensive care coordination models (General Practitioner Partnership Programme, Multilevel Bidirectional Care Coordination Model) and innovative stress management approaches including VR DE-STRESS and V-DESSERTS virtual reality-based programmes.

#### Theoretical foundation of the programmes.

Many of the programmes had theoretical underpinnings based on evidence drawn from overseas (n = 14), with Bandura’s self-efficacy theory (for depression), solution-focused brief therapy (for depression), relaxation theory (for stress), Cognitive Behavioural Therapy (CBT) for disruptive behaviours, emotional dysregulation, and obscessive compulsive disorders (OCD), CBT with Exposure and Response Prevention for OCD, the illness management and recovery model for those with various mental health disorders, life story review for depression being the common ones. Other theoretical foundations included the Theory of Psychosocial Development, gut-brain axis theory combined with mindfulness practices, collaborative care models for depression management, the Neuman System Model for VR-based stress interventions, and the Response, Early intervention and Assessment in Community mental Health (REACH) model’s five operating criteria for children and adolescent mental health services. Some programmes were developed in Singapore to suit the population, healthcare systems and cultural demands, for example, the GP and community centre partnerships to care for older adults in the community setting, whilst the others were adapted with minimal modifications.

#### Programme delivery and implementation.

The majority (n = 24) were conducted by clinicians (psychiatrists, psychologists, case managers, social workers, nurses or counsellors), whilst the rest were conducted by trained research staff, health coaches or volunteers. Seven programmes were conducted in participants’ homes, 10 in community settings, 2 in schools, 16 in hospitals (with 2 overlapping with participants’ homes), and the remainder in various settings including prisons, nursing homes, and social service agencies. Programme duration varied from single sessions to two years, while intensity of the session varied from daily practice to monthly sessions, with seven studies not reporting timing and dose. Most of the programmes demonstrated personalisation through various approaches (n = 26), including age-appropriate tailoring, language-specific allotments, disease severity adjustments, and participant choice and needs. Eleven programmes did not report any tailoring.

Twenty-eight programmes had no modifications from the original structure during implementation. Programme modifications included cultural adaptations through renaming to match the cultural context, pandemic-aligned recruitment strategies, additional module elements, and feedback-based improvements. Adherence reporting was inconsistent across studies. Where reported, it showed mixed responses ranging from moderate to high, with no clear correlation between dose and mode of delivery. Adult and adolescent-focused programmes primarily utilised technology-based or psychoeducational approaches. In healthcare settings, programmes mainly targeted adults and focused on continued care for those diagnosed with mental health conditions or at-risk populations (such as those at risk for postpartum depression). These primarily comprised educational interventions (psychoeducation or clinician-led education) delivered either face-to-face or through web platforms, mindfulness-based programmes, and management approaches. A detailed record of programme characteristics was captured using the TIDieR checklist and is presented in **S3 Table**.

#### Mental health outcomes and direction of evidence.

The outcomes captured across the 37 programmes included clinical symptoms (depression, anxiety, stress and other psychopathology), cognitive outcomes (such as decline in cognitive function, attention, memory and executive functions), functional capacity (Quality of life (QoL), activities of daily living), healthcare utilisation (referral, readmission, service utilisation, length of stay), and psychosocial outcomes (coping, social connection, caregiver burden, self-efficacy). Thirty-three (89.2%) studies employed validated questionnaires to capture the outcomes while three studies (8.1%) utilised hospital records and a single study (2.7%) included self-reported measures. Thirty-five studies (94.6%) reported positive outcomes observed as improved service utilisation, symptom severity, functional capacity and other wellbeing measures. The rest (n = 2) reported neutral/no effects. Overall, these programmes encompass a systematic, multi-faceted, tailored approach targeting the mental health of different populations across age groups and disease conditions, with the majority demonstrating positive outcomes through measurable improvements in clinical symptoms, service access and utilisation, and wellbeing indices.

### Programmes to improve mental health outcomes in those with physical health conditions

#### Types of programmes.

All 24 research articles were peer-reviewed publications. Programmes on Management and Treatment approaches were predominant (n = 9, 37.5%), followed by therapeutic interventions (n = 6, 25.0%), educational and psychoeducational interventions (n = 5, 20.8%), prevention and early intervention programmes and physical and activity-based programmes, each represented a smaller proportion (n = 2, 8.3% each), highlighting a strong emphasis on management-focused approaches rather than preventive or supportive interventions. The programmes addressed multiple health conditions and demonstrated considerable diversity in their therapeutic approaches. These ranged from technology-driven self-management interventions (Combined Diabetes and Renal Control Trial (C-DIRECT) for complex comorbidities, Chronic Disease Self-Management Programme (CDSMP) for self-care education), to tele-health monitoring (OPTIMUM for telemedicine-based diabetes management). Other programmes focused on psychological support (Cognitive Behavioral Therapy with Mindfulness and Values-based activity (CBT-MV), the Renewing Intimacy and SExuality Intervention (RISE) for cancer survivors, and the “Caring for the Caregiver Programme” for family support) and other creative programmes (music therapy, therapeutic play interventions, and biography and life story book (BLSB) approaches). These diverse programmes show the breadth of innovative treatment modalities employed to improve mental health outcomes in those with physical health conditions. The characteristics are presented in **Table 2**.

**Table 2:** Study characteristics for those targeting mental health outcomes in those with physical health conditions.

Author	Year	Institution	Population	Setting	Gender	Intervention/ Programme	Focus of the programme	Sample Size	Mental Health Outcomes	Measures	Direction
Chandran et al., 2024 [59]	2024	NUH	Adults, older adults	Hospital	Female = 70	Multidimensional rehabilitation programme	Breast cancer	N = 70	1. Cognitive function 2. Fatigue 3. QOL	1-2. FACT-G 3. EORTC QLQ-C30	Negative outcome
Guna et al., 2022 [60]	2022	Sunlove Nursing Home	Older adults	Nursing Home	Male = 49 Female = 25	Biography and life story book (BLSB)	Depression	N = 74	1. Depression 2. Life satisfaction 3. QOL	1. GDS-15 2. LSIA 3. QoL-NHR	Positive outcome
Griva et al., 2019 [61]	2019	NTU	Adults, older adults	Hospital	Male = 21 Female = 23	Combined Diabetes and Renal Control Trial (C-DIRECT) intervention	Psychosocial functioning	N = 44	1. Anxiety 2. Depression 3. Emotional wellbeing 4. Role emotional 5. Social functioning 6. Mental composite summary 7. Cognitive function 8. Quality of social interaction 9. Sleep 10. Social support 11. Positive and active engagement in life 12. Constructive attitudes and approaches 13. Self-monitoring and insight 14. Social integration and support 15. Emotional wellbeing	1-2. HADS 3-10. KDQOL 11-15. HEIQ	No effect
He et al. 2015 [62]	2015	NUS	Adults, children	Hospital	Parents Male = 17 Female = 78	Therapeutic play intervention	Anxiety	N = 190	1. Perioperative anxiety	1. SAS-C, SAS-A, qualitative interviews	No effect
Hoong et al., 2023 [63]	2023	JurongHealth Campus	Older adults	Community	Male = 146 Female = 369	Chronic Disease Self-Management Programme (CDSMP)	Health outcomes	N = 461	1. Self-efficacy 2. Social activities 3. Stress 4. Sleep 5. Cognitive symptom management 6. Depression	1. CDSES 2. Social/role Activities Limitations Scale 3-4. VNS 5. Cognitive Symptom Management Behaviour Change Scale 6. PHQ-8	Positive outcome
Leow et al. 2015 [64]	2015	NSC	Adults, older adults	Hospice, clinic	Male = 26 female = 54	Caring for the Caregiver Programme	Caregiver support	N = 80	1. Stress and depression 2. QOL 3. Social support 4. Closeness between caregivers 5. Self-efficacy 6. Positive gains of caregiver 7. Awareness of caregiver towards ACP	1. DASS 2. CQOLC 3. SSQ 4. General Closeness Scale 5. SCSES 6. RC Scale 7. Self-reported questionnaire	Positive outcome
Lim et al., 2014 [65]	2014	NUS	Adults, older adults	Hospital	Male = 5 Female = 13	Relaxation intervention	Psychological distress	N = 18	1. Stress 2. Relaxation 3. Self-efficacy 4. Anxiety	1. NSRS, ST 2. PRS 3. SES 4. STAI	Positive outcome
Lim et al., 2019 [66]	2019	SGH	NR	Hospital	Male = 10 Female = 3	Support availability, Thinking positively with acceptance, Overcoming social stigma, Minimizing negative feelings, Analysing self‐efficacy in stoma care (STOMA) psychosocial intervention programme	Coping	N = 13	1. Attitude towards STOMA	1. One to one interview	Positive outcome
Lim et al., 2019b [67]	2019	SGH	Adults	Hospital	Male = 33 Female = 18	STOMA programme	Coping	N = 51	1. Anxiety 2. Depression 3. QOL 4. Length of stay	1 and 2: HADS 3. EORTC QLQ-C29 4. Hospital records	No change
Mahendran etal., 2015 [68]	2015	NUS	Adults	Hospital	Male = 43 Female = 78	Brief nurse-led psychosocial intervention program	Psychological distress	N = 121	1. Distress 2. Anxiety 3. Depression 5. QOL	1. Distress Thermometer 2-3: HADS 4. EQ VAS	Positive outcome
Merchant et al., 2024 [69]	2024	NUS	Older adults	Primary Care	Male = 87 Female = 100	Exercise and Cognitive Stimulation Therapy	Intrinsic capacity	N = 187	1. Cognition 2. Psychological function	1. MoCA 2. GDS-15	Positive outcome
Neo et al., 2024 [70]	2024	NCSS	Adults, older adults	Hospital	Male = 65 Female = 34	Nurse-led telehealth programme	Palliative care	N = 99	1. Emotional concerns	1. IPOS	Positive outcome
Ramazanu et al., 2021 [71]	2021	PolyU	Adults Older adults	Hospital	Male = 7 Female = 7	3H (Hand, head and heart) intervention	Coping	N = 14	1. Coping	1. Interviews	Positive outcome
Tan et al., 2009 [72]	2009	SGH	Adults	Hospital	Male = 7 Female = 32	Group CBT programme	Chronic pain	N = 39	1. Use of Unhelpful Strategies 2. Problem-Solving Ability 3. Self-efficacy 4. Positive Statements 5. Negative Statements 6. Fear of Harm 7. Pathophysiological Beliefs 8. Depression 9. Anxiety 10. Stress	1-2. PSMC 3. PSEQ 4-5. PRSS 6-7. TSK 8-10. DASS	Positive outcome
Tan et al., 2024 [73]	2024	SHP	Adults	Primary Care	Male = 11 Female = 10	OPTIMUM—Optimising care of Patients via Telemedicine In Monitoring and Augmenting their control of Diabetes Mellitus	Self-management	N = 21	1. Self-efficacy	1. Semi-structured interviews	Positive outcome
Teo et al., 2020 [74]	2020	Duke-NUS	Adults, older adults	Hospital	Female = 95	Cognitive Behavioral Therapy with Mindfulness and Values-based activity (CBT-MV)	Advanced breast cancer	N = 95	1. Engagement 2. Psychological distress	1. Participant ratings 2. HADS	Positive outcome
Teo et al., 2020b [75]	2020	Duke-NUS	Adults, older adults	Hospital	Male = 37 Female = 23	CBT-based intervention	Colorectal cancer	N = 60	1. Psychological distress 2. Self-efficacy	1. HADS 2. CBI (Version 2.0)	Positive outcome
Teo et al., 2024 [76]	2024	NCSS	Adults	Hospital	Female = 33	Renewing Intimacy and SExuality Intervention (RISE)	Marital sexual distress	N = 33	1. Marital satisfaction 2. Sexual satisfaction 3. Sexual dysfunction 4. Body image disturbance	1. DAS-10 2. FSFI-Satisfaction subscale 3. ASEX 4. BIS	Positive outcome
Wang et al., 2015 [77]	2015	SGH	Adults	Hospital	Male = 380 Female = 809	Multi-component psychological intervention programme	Psychological wellbeing	N = 1189	1. Anxiety 2. Depression 3. QOL	1-2. HADS 3. IBS-QOL, EQ-5D	Positive outcome
Wang et al., 2018 [78]	2018	NUS	Adults, older adults	Community	Male = 115 Female = 14	Coronary Heart Disease Self‐management Programme (CHDSMP)	Self-management	N = 129	1. Anxiety 2. Depression 3. HrQOL 4. Perceived stress 5. Self-efficacy	1-2. HADS 3. SF-12 4. PSS 5. CSE Scale	No effect
Wong et al., 2021 [79]	2021	KKH	Children	Hospital	Male = 18, Female = 7	Music Therapy	Wellbeing	N = 25	Individualised goals achieved (physical and mental functioning)	1. Goal Attainment Scale	Positive outcome
Woo et al., 2022 [80]	2022	NUS	Adults, older adults	Hospital	Male = 29 Females = 14	Integrated Chronic Care E-Enhanced Atrial Fibrillation (NICE-AF)	Atrial fibrillation	N = 43	1. Depression	1. PHQ-9	Positive outcome
Yang et al., 2017 [81]	2017	TTSH	Adults	Hospital	Male = 8 Female = 25	iACT-CEL (Acceptance and Commitment Therapy intervention)	Chronic pain	N = 33	1. Global life satisfaction 2. Depression 3. Pain acceptance 4. General/psychological acceptance 5. Committed action	1. SWLS 2. PHQ-9 3. CPAQ-8 4. AAQ-II 5. CAQ	Positive outcome
Zhang et al., 2025 [82]	2025	CGH	Young adults, adults, older adults	Community	Male = 41 Female-22	Cancer Prehabilitation Exercise Diary	Prehabilitation	N = 63	1. Anxiety 2. Depression	1-2. HADS	No effect

NUH: National University Hospital, QOL: Quality of Life^,^ FACT-G: Functional Assessment of Cancer Therapy, EORTC QLQ-C30: European Organisation for Research and Treatment of Cancer Quality of Life Questionnaire-Core 30, GDS-15: Geriatric Depression Scale-15, LSIA: Life Satisfaction Index, QoL-NHR: Quality of Life for Nursing Home Residents, NTU: Nanyang Technological University, HADS: Hospital Anxiety and Depression Scale, KDQOL: Kidney Disease Quality of Life Short Form, HEIQ: Health Education Impact Questionnaire, NUS: National University of Singapore, SAS-C: State Anxiety Scale for Children, SAS-A: State Anxiety Scale for Adults, CDSES: Chronic Disease Self-Efficacy Scale, VNS: Visual Numeric Scale, PHQ-8: Patient Health Questionnaire-8, NSC: National Skin Centre, ACP: Advanced Care Planning, DASS: Depression Anxiety Stress Scale, CQOLC: The Caregiver Quality of Life Index–Cancer, SSQ: Social Support Questionnaire, SCSES: Self Care Self Efficacy Scale, RC Scale: Rewards of Caregiving Scale, NSRS: Numeric Stress Rating Scale, ST: Stress Thermometer, PRS: Perceived Relaxation Scale, SES: Self-efficacy Expectation Scale, STAI: State-Trait Anxiety Inventory, SGH: Singapore General Hospital, EORTC QLQ-C29: European Organisation for Research and Treatment of Cancer Quality of Life Questionnaire Colorectal 29-item questionnaire, EQ VAS: EuroQoL Visual Analogue Scale, MoCA: Montreal Cognitive Assessment, NCSS: National Cancer Centre Singapore, IPOS: Integrated Palliative Outcome Scale, PolyU: The Hong Kong Polytechnic University, PSMC: Pain Self-Management Checklist, PSEQ: Pain Self-Efficacy Questionnaire, PRSS: Pain Response Self-Statement, TKS: Tampa Scale for Kinesiophobia, SHP: SingHealth Polyclinics, Duke-NUS: Duke-NUS Medical School, CBI: Cancer Behaviour Inventory, DAS-10: Dyadic Adjustment Scale-10, FSFI: Female Sexual Functioning Index, ASEX: Arizona Sexual Experience Scale, BIS: Body Image Scale, IBS-QOL: Irritable Bowel Syndrome Quality of Life, European Quality of Life (EuroQOL) 5 Dimensions, HrQOL: Health-related Quality of Life, SF-12: 12-Item Short Form Health Survey, PSS: Perceived Stress Scale, CSE Scale: Cardiac Self Efficacy Scale, KKH: KK Women’s and Children’s Hospital, PHQ-9: Patient Health Questionnaire-9, TTSH: Tan Tock Seng Hospital, SWLS: Satisfaction with Life Scale, CPAQ-8: Chronic Pain Acceptance Questionnaire-8, AAQ-II: Acceptance and Action Questionnaire-II, CAQ: Committed Action Questionnaire, CGH: Changi General Hospital

### Characteristics of the programmes

#### Population and setting.

The majority of studies focused on patients alone (n = 21, 87.5%), with 2 studies focusing on caregivers (8.3%) and one for nursing home residents (n = 1, 4.2%). Among the 24 programmes, 3 were solely for older adults (12.5%), 7 only for adults (29.2%), 11 for both adults and older adults (45.8%), 1 for adult and child (4.2%), 1 for children and 1 for unreported age groups (4.2%). Nineteen programmes were conducted in healthcare settings (79.2%), 3 in community settings (12.5%), 1 in a nursing home (4.2%), and 1 in combined hospice and clinic settings (4.2%). There was no age-specific variation in programme settings across different age groups.

#### Disease specific approaches.

The programmes addressed diverse health conditions with cancer being the common focus of the studies (n = 11, 45.8%), followed by chronic conditions, cardio/cerebrovascular conditions (n = 3 each, 12.5%), chronic pain (n = 2, 8.3%), and other physical conditions (gastrointestinal, surgical interventions, knee pathology, surgery, other physical ailment) (n = 1 each, 4.2% each).

The programmes were tailored to disease pathology, symptom burden, comorbidities and management needs of different conditions. For cancer (n = 11, 45.8%), the programmes addressed physical and mental health functioning, cognitive functions, quality of life, emotional concerns and other challenges in oncology care. The interventions included CBT-based therapies, mindfulness, psychosocial interventions and rehabilitation programmes. For cardiovascular diseases (n = 3, 12.5%), the programmes addressed long-term management challenges to empower patients in home-based condition management, to provide post-stroke couple support focusing on information, decision-making and practical skills, and to shift them towards integrated chronic care models. Programmes targeting chronic conditions (n = 3, 12.5%) focused on self-management and self-efficacy of the disease and comorbidities through motivational interviews, group therapies and psychoeducation. Programmes for other conditions addressed disease-specific challenges through Acceptance and Commitment Therapy (ACT), CBT, cognitive stimulation, life story books, relaxation interventions, and psychological support.

#### Population specific approach.

The programmes targeted different age groups with mixed populations (adults and older adults) being the most common (n = 11, 45.8%), followed by adults (n = 9, 37.5%), older adults (n = 3, 12.5%) and children (n = 1, 4.2%). The programmes demonstrated distinct approaches tailored to the developmental needs, health challenges, and care preferences of different population groups.

The programmes for older adults addressed complex comorbidities (chronic kidney disease, diabetes complications, etc.) and their impact on life. The content was aimed at maintaining independence while addressing age-related vulnerabilities through support systems. These programmes focused on improving coping, psychological wellbeing, and disease management whilst providing essential psychosocial support, including life story book reviews, cognitive stimulation, and self-management programmes.

The programmes for adults focused on active disease management, symptom control and psychological coping, caregiver support, psychoeducation and maintaining functional capacity across different diseases. These included tailored interventions such as internet-based therapies and telehealth for disease management which addressed both physical and psychological wellbeing of the population. Programmes for children were designed to create awareness of surgical and hospitalisation processes to reduce anxiety and promote wellbeing through play interventions.

#### Theoretical foundations of the programmes.

The theoretical underpinnings of the programmes (n = 15, 62.5%) included frameworks such as self-efficacy theory, based on Bandura’s work (n = 6, 25.0%), which was mainly adopted by programmes targeting chronic disease management, caregiver support, stoma care, pain management, and diabetes care. This theory’s emphasis on personal mastery, vicarious experiences, verbal persuasion, and physiologic feedback were translated to the self-management focus of many interventions. CBT formed the theoretical basis for several programmes (n = 4, 16.7%), particularly those addressing psychological outcomes in gastrointestinal disorders, chronic pain, and cancer. Other psychological frameworks adopted by the programmes included ACT and the psychological flexibility model for chronic pain management, social cognition theory combined with motivational interviewing for complex comorbidities, and the health belief model for diabetes self-management.

Several programmes incorporated locally adapted frameworks, such as the biography and life story book intervention developed using Agency for Integrated Care (AIC) Dementia Resource Kit, and the chronic care management model with integrated care approaches shifting from traditional clinician-centric hospital care. Some programmes (n = 9, 37.5%) did not explicitly state their theoretical foundations, though they demonstrated evidence-based approaches through structured interventions combining health education, stress management, behavioural training, and psychosocial support tailored to specific patient populations and clinical contexts.

#### Programme delivery and implementation.

Overall, the majority of the programmes (n = 15, 62.5%) were delivered face to face with 2 delivered through mobile apps, 1 through online, and the rest were through hybrid mode. The main content of the programme included psychoeducation, psychotherapy and included behavioural therapies such as ACT, CBT and mindfulness. These programmes were delivered mainly by trained clinicians (n = 21, 87.5%) and varied in duration and intensity from 3 days to 6 months. Programmes for chronic conditions were aimed at creating awareness of long-term complications and improving the self-management of the conditions, with education, and CBT being common elements. Tailoring was done for 10 (41.7%) programmes and included adaptations for local use, (e.g., languages, activities, population characteristics), flexible schedules, level of risk, and symptom severity. Modifications were implemented for 5 programmes only (20.8%) and included pandemic specific adaptations. Adherence to the programme showed mixed results. A detailed list of items as per the **TIDieR checklist is given in** **S4 Table**.

#### Mental Health outcomes and direction of evidence.

The studies measured multiple outcomes including anxiety, depressive symptoms, self-efficacy, cognition (cognitive symptom management, cognitive functions), coping, quality of life and life satisfaction, using validated measurement tools. Programmes also measured psychological and psychosocial domains such as emotional wellbeing, social functioning and interpersonal relationships, with marital and sexual satisfaction included among the measures. Outcomes aligned with the health contexts. For example, cancer-related programmes measured body image concerns, caregiver burden and awareness towards advance care. Patient experiences were also captured in some programmes, which shows the breadth of mental health outcomes and the recognition of the importance of psychological wellbeing in those with physical health conditions rather than merely symptom reduction and management of functional capacity. Three studies (12.5%) employed semi-structured interviews to capture the outcomes, while the rest employed validated questionnaires. Eighteen studies (75.0%) showed a positive outcome, and one showed a negative outcome (4.2%), and five (20.8%) reported no changes.

### Programmes to improve mental health outcomes in those with neurodevelopmental and learning conditions

#### Types of programmes.

All studies (n = 8) were peer-reviewed publications. The programmes were focused on educational and psychoeducational approaches (n = 5, 62.5%), management and treatment (n = 2, 25.0%) and prevention and early intervention initiatives (n = 1, 12.5%). Educational interventions included technology driven programmes such as the Immersive Interactive Mixed Reality (IMR) educational intervention for enhanced learning experiences, literacy-focused programmes such as the English Main Literacy Programme and Lexicaid, which provide language support for those with learning difficulties. Management approaches included behavioural interventions such as the Signposts for Building Better Behaviour programme and innovative attention training through The brain-computer interface (BCI) based Attention Training Game System. Therapeutic interventions encompassed cognitive-behavioural therapy and combined traditional behavioural approaches with natural play interventions.

### Characteristics of the programmes

#### Population and setting.

Regarding study settings, educational or special educational institutions (n = 4, 50.0%) and hospitals (n = 3, 37.5%) were most common, with community settings (n = 1, 12.5%). The study populations mainly comprised children (n = 6, 75.0%), both children and adolescents (n = 1, 12.5%) and adults (n = 1, 12.5%).

#### Disease specific approaches.

Three studies (37.5%) included programmes targeting dyslexia, 2 (25.0%) for autism spectrum disorder (ASD), and 1 each (12.5%) for intellectual disability, developmental disability, and attention deficit hyperactivity disorder (ADHD). The programmes followed approaches specific to each condition. Dyslexia-focused programmes utilised the English Main Literacy Programme for targeted literacy development, alongside Lexicaid for specialised language support. ASD-related programmes incorporated the Signposts for Building Better Behaviour programme for behavioural management and traditional behavioural approaches combined with natural play interventions to address social communication and behavioural challenges. The ADHD-targeted programme employed The BCI-based Attention Training Game System, utilising brain-computer interface technology specifically designed for attention enhancement. Programmes addressing intellectual and developmental disabilities implemented CBT based approaches adapted for cognitive and developmental needs, while the IMR educational intervention provided technology-enhanced learning experiences suitable for various neurodevelopmental conditions.

#### Population specific approach.

All programmes targeted paediatric populations. Age-specific adaptations were made to accomodate cognitive capacity and developmental stages, with the majority of the programmes employing behavioural and play-based interventions and educational technologies (BCI attention training) that leverage children’s interest in interactive platforms while addressing the learning and attention challenges.

#### Theoretical foundations of the programmes.

The programmes demonstrated strong theoretical underpinnings, with the majority (n = 6, 75.0%) grounding their interventions in established evidence-based frameworks. Educational interventions for dyslexia were predominantly based on the Orton-Gillingham (OG) principles and incorporated evidence from the National Reading Panel (US), Professional Practice Guidelines (Singapore), and the Rose Report (UK). The PPP (Presentation, Practice, Production) stages approach provided structured learning progressions for literacy interventions. Therapeutic interventions employed manualised CBT protocols adapted for high-functioning children with autism spectrum disorder, emphasising anxiety reduction and stress management. Behavioural interventions employed applied behaviour analysis principles, comparing traditional behavioural approaches with natural play interventions for ASD. Technology-related interventions integrated neurofeedback theory through brain-computer interface attention training, whilst immersive interactive mixed reality interventions combined educational technology theory with cognitive disability support frameworks.

#### Programme delivery and implementation.

Programmes were delivered in educational settings (n = 3, 37.5%), hospitals (n = 3, 37.5%), community settings (n = 1, 12.5%) and in special needs schools (n = 1, 12.5%), focusing on children and adolescents with an aim to improve learning and self-management domains. These were delivered by educators or therapists through face-to-face sessions. The duration varied from weeks to several months. These were tailored to students’ needs and characteristics, though only one of the programmes reported modifications to match the pandemic regulations. High adherence was reported across all studies with no withdrawals. A detailed TIDieR checklist is included in **S5 Table**.

#### Mental health outcomes and direction of evidence.

The studies looked at various outcomes such as anxiety, stress, wellbeing, symptom severity, social and academic skills. Seven studies (87.5%) employed validated questionnaires and a single study used curriculum-based assessments (12.5%). All studies reported positive outcomes in all measured domains (100%; **Table 3**).

**Table 3:** Study characteristics for those targeting mental health outcomes in those with neurodevelopmental and learning conditions.

Author	Year	Institution	Population	Setting	Gender	Intervention/ Programme	Focus of the programme	Sample Size	Mental Health Outcomes	Measures	Direction
Bernard-Opitz et al., 2004 [83]	2004	NUS	Children	Hospital	NR	Traditional behavioural approaches and natural play interventions	Autism	N = 8	1. Autism scores	1. PL-ADOS, SPT	Positive outcome
Fitriya., 2021 [84]	2021	DAS	Children	School	NR	English Main Literacy Programme	Dyslexia	N = 1343	1. Progress of students (reading writing and learning)	Curriculum-Based Assessment	Positive outcome
Fitriya., 2022 [85]	2022	DAS	Children	School	NR	English Main Literacy Programme	English literacy	N = 1280	Mean percentage scores for reading, writing and spelling	Curriculum-Based Assessment	Positive outcome
Lim et al., 2012 [86]	2012	IMH	Children	Hospital	Male = 16 Female = 4	The BCI-based Attention Training Game System	ADHD	N = 20	1. ADHD symptoms 2. ADHD severity	1. ADHD-RS 2. BASM	Positive outcome
Ooi et al., 2008 [87]	2008	IMH	Children	School	NR	Cognitive-behavioural therapy	Anxiety	N = 6	1. Anxiety 2. Parental stress 3. Teachers’ stress	1. SCAS-C, SPAC-P, ACAS 2. PSI 3. ITS	Positive outcome
Tan et al., 2022 [88]	2022	SUTD	Children	Community	Male = 20 Female = 11 (drop outs unknown)	Lexicaid	Learning and psychosocial well being	N = 45	1. Writing competence 2. Reading competence 3. Spelling competence 4. General intelllectual ability	1-4. SPPLD	Positive outcome
Tan et al., 2024 [89]	2024	Digital Dream Singapore	Children, adolescents	School	NR	Immersive Interactive Mixed Reality (IMR) educational intervention	Psyschosocial functioning	N = 86 (students) N = 10 (teachers)	1. Mental wellbeing 2. Social skills 3. Teachers’ work satisfaction 4. Teachers’ sense of efficacy	1. SWEMWBS 2. Social Skills Checklist 3. UWES 4. TSES	Positive outcome
Yap et al., 2019 [90]	2019	KKH	Adults	Hospital	Female = 285	Signposts for Building Better Behaviour programme	Parenting skills	N = 285	1. Parenting efficacy	1. PSOC	Positive outcome

NUS: National University of Singapore, PL-ADOS: Pre-Linguistic Autism Diagnostic Observation Schedule, SPT: Symbolic Play Test, DAS: Dyslexia Association of Singapore, IMH: Institute of Mental Health, ADHD-RS: ADHD Rating Scale, BASM: BCI ADHD Severity Measure, SCAS-C: Spence Child Anxiety Scale-Child, SCAS-P: Spence Child Anxiety Scale-Parent, ACAS: Asian Children Anxiety Scale-Caretaker Version, PSI: Parenting Stress Index, ITS: Index of Teaching Stress, SUTD: Singapore University of Technology and Design, SPLLD: Self-Perception Profile for Learning Disabled Students, SWEMWBS: Modified Short Warwick-Edinburgh Mental Wellbeing Scale, UWES: Utrecht Work Engagement Scale, TSES: Sense of Teacher Efficacy Scale, PSOC: Parenting Sense of Competence

### Programmes to improve mental health of healthy population (no specific disease)

#### Types of programmes.

All (n = 37) studies were peer-reviewed publications. The study characteristics are indicated in **Table 4**. Among the programmes, educational and psychoeducational approaches were most common (n = 14, 37.8%), followed by prevention and early intervention (n = 13, 35.1%), therapeutic interventions (n = 4, 10.8%), other programmes (wellbeing, performance improvement programmes, n = 4, 10.8%), and physical and activity-based interventions (n = 2, 5.4%). These programmes were intended to improve health behaviours (n = 6, 16.2%; includes health literacy, help-seeking), mental health stigma (n = 3, 8.1%), wellbeing (n = 14, 37.8%; overall, postnatal), and cognitive functions (n = 3, 8.1%). Other target areas included psychological distress and parenting skills (n = 2 each, 5.4% each), anxiety, suicidality, and attitudes towards those with intellectual disability, obesity management, peer support for domestic workers, stress, burnout, and work performance (n = 1 each, 2.7% each). Technology-driven programmes were common, including mobile health applications such as the Headspace mindfulness app, Home-but-not-Alone application, and Supportive Parenting App, alongside web-based platforms like the Live Chat online counselling (Ask iZ Master) and Online HOPE interventions. Advanced technology approaches included Brain-Computer Interface Based Cognitive Training Systems and computerised cognitive training through the NeeuroFIT programme. Evidence-based approaches such as Functional Family Therapy, CBT, and mindfulness-based wellness programmes were also included. Educational and psychoeducational programmes featured anti-stigma and disability awareness, neonatal care and postnatal care programmes. Activity-based interventions such as horticulture, exergames and pet therapy were also employed. Specialised programmes such as driver retirement programmes and better behaviour programmes were intended for those with special support needs.

**Table 4:** Study characteristics for those targeting mental health outcomes in healthy population.

Author	Year	Institution	Population	Setting	Gender	Intervention/ Programme	Focus of the programme	Sample Size	Mental Health Outcomes	Measures	Direction
Ang, 2018 [91]	2018	TTSH	NR	Hospital	NR	Suicide management programme	Suicide	N = 7	Completed suicides	1. Actual numbers	Positive outcome
Bos at al., 2018 [92]	2018	KKH	Adults	Hospital	Male = 3 Female = 39	Neonatal discharge programme (parentcraft teaching)	Psychological distress	N = 42	1. Parental efficacy 2. Psychological distress	1. PSES 2. DASS-21	Positive outcome
Chong et al., 2024 [93]	2024	NTFGH	Adults, older adults	Community	Male = 3 Female = 16	Get Well, Live Well programme	HL, health behaviours	N = 19	1. Perceptions and experiences of the Get Well, Live Well programme	1. Semi-structured interviews	Positive outcome
Gan et al., 2021 [94]	2021	MSF	Young adults	Community	Male = 107 Female = 13	Functional Family Therapy	Wellbeing	N = 120	1. Mental wellbeing 2. Perceived family functioning	1. YOQSR2.0 2. FAD‐GF	Positive outcome
Keng et al., 2022 [95]	2022	Duke-NUS	Adults	Online	Female = 72	Headspace mobile app-based mindfulness practice	Psychological distress	N = 80	1. Depression 2. Anxiety 3. Fear of COVID-19 4. PTSD symptoms 5. Personal wellbeing 6. Compassion satisfaction 7. Burnout 8. Perceived sleep quality 9. Trait mindfulness 10. Self-compassion 11. Digit-span forward 12. Digit-span backward	1-2. DASS-21 3.FCV-19S 4. PCL-C 5. Personal Wellbeing Index 6-7. ProQoL – Burnout and Compassion Satisfaction subscales 8. PSQI 9. FFMQ 10. SCS 11-12. DGS – Forward and Backward from WAIS	Positive outcome
Kit et al., 2019 [96]	2019	NTU	Children	School	Male = 18 Female = 15	Live Chat online counselling (Ask iZ Master) via iZ Hero Challenge portal	Help-seeking	N = 33	1. Qualititative experiences	1. Qualitative interviews	Positive outcome
Lee at al., 2013 [97]	2013	Duke-NUS	Older adults	Community	Male = 12 Female = 19	Brain-Computer Interface Based Cognitive Training System	Cognitive function	N = 31	1. Cognitive function	1. RBANS	Positive outcome
Lee at al., 2015 [98]	2015	Duke-NUS	Older adults	Community	Male = 12 Female = 27	Brain-Computer Interface Based Cognitive Training System	Cognitive function	N = 39	1. Cognitive function	1. RBANS	Positive outcome
Lee et al., 2020 [99]	2020	Duke-NUS	Adolescents	Community	Male = 100 Female = 211	Social norm-based intervention for physical activity	Obesity	N = 311	1. QOL 2. Depression 3. Social support for exercise 4. Physical activity self efficacy 5. Physical activity enjoyment	1. Peds-QL 2. AADS 3. SSE 4. PASES 5. PACES-8	No effect
Leong et al., 2022 [100]	2022	CGH	Older adults	Community	Male = 5 Female = 10	Intergenerational Programme	Wellbeing	N = 15	1. Emotional responses, attitudes and perceptions towards younger generation	1. Thematic analysis	Positive outcome
Li et al. 2014 [101]	2014	NTU	Young adults	University	Male = 18 female = 24	Disability awareness programme	Attitude towards intellectual disability	N = 42	1. Attitude towards disability	1. CLAS, Reflection journals	Positive outcome
Lim et al., 2024 [102]	2024	NUS	Young adults	School	NR	Educational	HL, health behaviours	N = 131	1. Mental health knowledge 2. Mental health attitudes 3. Mental health practices	1-3. 5-point Likert scale questions	Positive outcome
Lim et al., 2024 [103]	2024	NTFGH	Older adults	Community	Male = 112 Female = 352	“Wise and Well” programme	Health behaviours	N = 464	1. Lifestyle goal attainment	1. Goal Attainment Scale	Positive outcome
Chan et al., 2015 [104]	2015	TTSH	Older adults	Community	NR	Driver Retirement Programme	Wellbeing	N = 15	1. Depression 2. Self-efficacy 3. Goal satisfaction 4. HrQOL	1. GDS 2. Self-efficacy questionnaire 3. Individual goal satisfaction 4. SF-36 v2	Positive outcome
Metrat-Depardon and Teo, 2023 [105]	2023	NTU	Young adults	School	Male = 12 Female = 22	Happiness Mentoring Programme	Psychological wellbeing	N = 34	1. Adolescent well being 2. Happiness 3. Feedback on the programme	1 and 2. PERMA-Profiler questionnaire with added items from EPOCH Measure of Adolescent Wellbeing, Children’s Hope Scale, Gratitude Questionnaire and SWLS 3. Open ended questions on feedback	Positive outcome
Muckle and Lasikiewicz, 2017 [106]	2017	JCU	Young adults	School	Male = 11 Female = 51	Animal assisted activities	Wellbeing	N = 62	1. Stress 2. Anxiety 3. Self-esteem	1. PSS 2. STAI 3. SSES	Positive outcome
Ng et al., 2018 [107]	2018	NUS	Older adults	Community	Male = 8 Female = 72	Horticultural therapy	Mental wellbeing	N = 59	1. Cognitive function 2. Depression 3. Anxiety 4. Psychological wellbeing 5. Social connectedness 6. Satisfaction with life	1. MoCA 2. SDS 3. SAS 4. SPWB 5. FS 6. SWLS	Positive outcome
Yeo et al., 2021 [108]	2021	GERI	Older adults	Community	Male = 26 Female = 68	Computerized cognitive training (CCT) NeeuroFIT programme	Cognitive function	N = 94	1. Cognition 2. Gait	1. CTT-2, RBANS, BBS 2. GAITRite walkway measures	Positive outcome
Shahwan et al., 2020 [109]	2020	IMH	Young adults	University	Male = 155 Female = 235	Anti-stigma intervention	Mental health stigma	N = 390	1. Psychological openness 2. Help-seeking propensity 3. Indifference to stigma	1-3. IASMHS	Positive outcome
Shorey et al., 2015 [110]	2015	NYP	Adults	Hospital	Female = 18	Postnatal psychoeducation programme	Postnatal care	N = 18	1. Maternal confidence 2. Emotional wellbeing	1-2. Semi-structured interviews	Positive outcome
Shorey et al., 2017 [111]	2017	NUS	Adults	Hospital	Male = 125 Female = 125	Home-but not Alone mobile health application	Postpartum parenting outcomes	N = 250	1. Parental self-efficacy 2. Social support 3. Postnatal depression 4. Parenting satisfaction	1. PES 2. PSSP 3. EPDS 4. WBPL- Satisfaction subscale	Positive outcome
Shorey et al., 2023 [112]	2023	NUS	Adults	Hospital	Male = 200 Female = 200	Supportive Parenting App (SPA)	Postnatal wellbeing	N = 400	1. Postnatal depression 2. Anxiety 3. Parental bonding 4. Parental self-efficacy 5. Social support	1. EPDS 2. STAI 3. PIBQ 4. PES 5. PSSP 6. WPBL	Positive outcome
Sia, A., et al., 2020 [113]	2020	NUS	Older adults	Community	Male = 14 Female = 33	Therapeutic horticulture programme	Psychological wellbeing	N = 47	1. Happiness 2. Anxiety 3. Depression 4. Cognitive function 5. Sleep quality 6. Social connectedness and loneliness	1. EQ VAS 2. STAI 3. SDS 4. MMSE 5. PSQI 6. FS	Postive outcome
Subramaniam et al., 2020 [114]	2020	IMH	Young adults	University	Male = 155 Female = 236	Anti-stigma intervention	Mental health stigma	N = 390	1. Depression literacy 2. Personal stigma 3. Social distance	1. D-Lit 2. DSS – Personal Stigma subscale 3. Social Distance Scale	Positive outcome
Suyi et al., 2017 [115]	2017	IMH	Young adults, adults, older adults	Hospital	Male = 7 Female = 30	Mindfulness-based training programme	Stress and burnout	N = 37	1. Mindfulness 2. Self-compassion 3. Compassion for others 4. Perceived stress 5. Burnout	1. FFMQ 2.SCS-SF 3. CS 4. PSS-10 5. OLBI	Positive outcome
Tan and Mankiewicz, 2024 [116]	2024	NUS	Young adults	Community	Male = 31 Female = 137	Brief video contact-based intervention	Mental health stigma	N = 168	1. Social distancing attitudes 2. Tolerance/support for community care attitudes 3. Social restrictiveness attitudes 4. Prejudice and discrimination attitudes	1-4. AMI-SG	Positive outcome
Tan et al., 2021 [117]	2021	IMH	Young adults	University	Male = 155 Female = 236	Advancing Research Toward Eliminating Mental Illness Stigma (ARTEMIS)	Help-seeking, MHL	N = 390	1. Recognition of depression 2. Help-seeking beliefs	1-2. Vignettes	Positive outcome
Tay et al., 2022 [118]	2022	IMH	Young adults	Online	Male = 50, Female = 124	Online HOPE Intervention	Pyschological wellbeing, MHL	N = 174	1. Depression literacy 2. Anxiety literacy 3. Personal stigma 4. Psychological wellbeing 5. Perceived stress	1. D-Lit 2. A-Lit 3. DSS – Personal Stigma subscale 4. SWPB (18-item) 5. PSS	Positive outcome
Tay, 2022b [119]	2022	IMH	Young adults	Online	Male = 50, Female = 124	Online HOPE intervention	Help-seeking	N = 174	1. Recognition of depression 2. Help-seeking intentions	1-2. Individual semi-structured phone interviews	Positive outcome
Wong et al., 2018 [120]	2018	SGH	Adults	Hospital	Male = 2 Female = 34	Mindfulness-based training programme for nurses	Work performance	N = 36	1. Sustained attention	1. PVT, EEG Responses	Positive outcome
Wong et al., 2020 [121]	2020	NUS	Adults	Community	Female = 39	Mental health paraprofessional training for FDWs using CBT principles	Peer support	N = 39	1. Depression literacy 2. Knowledge of CBT 3. Confidence in supporting individuals with depression 4. Help-seeking attitudes 5. Attitudes towards depression	1. D-Lit 2. Knowledge of CBT questionnaire 3. Single item scale 4. ATSPPH-SF 5. DSS	Positive outcome
Yap et al., 2014 [122]	2014	KKH	Adults, older adults	Hospital	Male = 374 Female = 641 Caregivers (grandparents) = 6 (gender not available)	Signposts for Building Better Behaviour programme	Parenting skills	N = 1021	1. Parental efficacy and satisfaction 2. Depression 3. Anxiety 5. Stress 6. Hassles 7. Child’s difficult behaviour 8. Child aggression and compliance	1. PSOC 2-5. DASS 6. PHS 7. DBC 8. DBAF	Positive outcome
Yap et al., 2017 [123]	2017	Duke-NUS	Older adults	Community	Male = 2 Female = 29	Rhythm Wellness Programme	Psychological wellbeing	N = 54	1. QOL 2. Depressive mood 3. Sleep quality 4. Social isolation	1. EQ-5D 2. GDS 3. PSQI 4. LSNS	No effect
Yeo et al., 2016 [124]	2016	NTU	Children	School	Male = 70 Female = 45	School-based intervention for test anxiety	Test anxiety	N = 115	1. Test anxiety	1. CTAS	Positive outcome
Zheng et al., 2020 [125]	2020	NTU	Older adults	Community	Male = 63 Female = 257	Exergames (exercise games)	Wellbeing	N = 319	1. Sociability 2. Positive and negative affect	1. Sociability Scale 2. PANAS	Positive outcome
Zheng et al., 2022 [126]	2022	NUHS	Adults	Hospital	Female = 204	Web-based and home-based postnatal psychoeducational interventions	Postnatal wellbeing	N = 204	1. Maternal parental self-efficacy 2. Social support 3. Postnatal depression 4. Anxiety	1. PMPSE 2. PICSS 3. EPDS 4. HADS-A	Positive outcome
Zhou et al., 2017 [127]	2017	MSF	Adults	Community	Male = 37 Female = 70	Positive Parenting Programme (Triple P)	Parenting skills	N = 107	1. Caregiver perception of child’s behaviour problem 2. Parenting style 3. Depression 4. Anxiety 5. Stress 6. Parenting self efficacy 7. Parenting satisfaction 8. Attribution style for child’s disruptive behaviours 9. Parental anger in response to child caring	1. ECBI 2. PS 3-5. DASS 6-7. BAPS 8. PACBM 9. PAI	Positive outcome

TTSH: Tan Tock Seng Hospital, KKH: KK Women’s and Children’s Hospital, PSES: Parenting Self Efficacy Scale, DASS-21: Depression Anxiety Stress Scale-21, NTFGH: Ng Teng Fong General Hospital, HL: Health Literacy, MSF: Ministry of Social and Family Development, YOQSR2.0: Youth Outcome Questionnaire Self‐Report Version 2.0, FAD-GF: Family Assessment Device—General Functioning Scale, Duke-NUS: Duke-NUS Medical School, FCV-19S: Fear of COVID-19 Scale, PCL-C: Posttraumatic Stress Disorder Check List – Civilian Version, ProQoL: Professional Quality of Life, PSQI: Pittsburgh Sleep Quality Index, FFMQ: Five Facet Mindfulness Questionnaire, SCS: Self Compassion Scale, DGS: Digit Span, WAIS: Wechsler Adult Intelligence Scale, NTU: Nanyang Technological University, RBANS: Repeatable Battery for the Assessment of Neuropsychological Status, Peds-QL: Pediatric Quality of Life, AADS: Asian Adolescent Depression Scale, SSE: Social Support and Exercise, PASES: Physical Activity Self-Efficacy Scale, PACES-8: Physical Activity Enjoyment Scale, CGH: Changi General Hospital, CLAS: Community Living Attitudes Scale, NUS: National University of Singapore, HrQOL: Health-related Quality of Life, GDS: Geriatric Depression Scale, SF-36: 36-Item Short Form Health Survey, SWLS: Satisfaction with Life Scale, JCU: James Cook University, PSS: Perceived Stress Scale, STAI: State-Trait Anxiety Inventory, SSES: State Self Esteem Scale, MoCA: Montreal Cognitive Assessment, SDS: Zung Self-Rating Depression Scale, SAS: Zung Self- Rating Anxiety Scale, SPWB: Ryff’s Scales of Psychological Wellbeing, FS: Friendship Scale, GERI: Geriatric Education and Research Institute, CTT: Colour Trails Test, BBS: Berg Balance Scale, IMH: Institute of Mental Health, IASMHS: Inventory of Attitudes toward Seeking Mental Health Services, NYP: Nanyang Polytechnic, PES: Parenting Efficacy Scale, PSSP: Perceived Social Support for Parenting, EPDS: Edinburgh Postnatal Depression Scale, WBPL: What Being the Parent of a New Baby Is Like, PIBQ: Parent-to-Infant Bonding Questionnaire, EQ VAS: EuroQoL Visual Analogue Scale, MMSE: Mini-Mental State Examination, D-Lit: Depression Literacy Questionnaire, DSS: Depression Stigma Scale, SCS-SF: Self-Compassion Scale-Short Form, CS: Compassion Scale, PSS-10: Perceived Stress Scale-10, OLBI: Oldenburg Burnout Inventory, AMI-SG: Attitudes to Mental Illness questionnaire Singapore version, MHL: Mental Health Literacy, A-Lit: Anxiety Literacy Questionnaire, SGH: Singapore General Hospital, PVT: Psychomotor Vigilance Task, EEG: Electroencephalogram, FDW: Foreign Domestic Worker, ATSPPH-SF: Attitudes Toward Seeking Professional Psychological Help Scale – Short Form, PSOC: Parenting Sense of Competence, DASS: Depression Anxiety Stress Scale, PHS: Parenting Hassles Scale, DBC: Developmental Behaviour Checklist, DBAF: Difficult Behaviour Assessment Form, EQ-5D-5L: European Quality of Life (EuroQOL) 5 Dimensions questionnaire, LSNS: Lubben Social Network Scale, CTAS: Children’s Test Anxiety Scale, PANAS: Positive and Negative Affect Schedule, NUHS: National University Health System, PMPSE: Perceived Maternal Parental Self-efficacy tool, PICSS: Perinatal Infant Care Social Support, BAPS: Being a Parent Scale, PACBM: Parental Attributions for Child’s Behaviour Measure, PAI: Parental Anger Inventory

### Characteristics of the programmes

#### Population and setting.

Nearly half of the studies (n = 16, 43.2%) were conducted in community settings, 9 each in hospitals and educational institutions (24.3% each), and 3 were hosted online (8.1%). The programmes targeted diverse age groups with young adults being the most commonly targeted population (n = 11, 29.7%), followed by older adults (n = 10, 27.0%) and adults (n = 9, 24.3%). Adolescents (n = 1, 2.7%) and children (n = 2, 5.4%) were less frequently targeted. Three programmes (8.1%) targeted multiple age groups, including young adults with adults and older adults, and adults with older adults. One programme (2.7%) did not report the target population.

#### Disease specific approaches.

The population had no specific disease. Hence, no specific adaptations were made for the programmes.

#### Population-specific approaches.

The programmes for older adults addressed age-related mental health concerns including cognitive decline, social isolation, and late-life transitions. The content was aimed at maintaining cognitive function and psychological wellbeing while addressing vulnerabilities through community-based support systems. These programmes focused on improving mental wellbeing, cognitive stimulation, and social engagement through interventions such as horticultural therapy, exergames, brain-computer interface training, and wellness programmes that provided essential psychosocial support and cognitive enhancement.

The programmes for adults focused on work-related stress management, parenting support, psychological coping, and mental health promotion across various life domains. These included tailored interventions such as mobile health applications, online counselling platforms, and mindfulness-based training which addressed both psychological wellbeing and functional capacity in workplace and family contexts. Programmes for young adults were designed to address transitional life challenges, university-related stress, stigma reduction, and help-seeking behaviours through technology-enhanced interventions and peer support systems. Programmes for children focused on reducing anxiety, promoting emotional wellbeing, and developing coping skills through school-based interventions and online counselling platforms designed for their developmental stage.

#### Theoretical foundations.

The programmes demonstrated strong theoretical foundations, with the majority (n = 28, 75.7%) explicitly basing their interventions in evidence-based frameworks. Bandura’s Self-Efficacy Theory was the most frequently employed theoretical foundation (n = 6, 16.2%), particularly in parenting programmes and health behaviour interventions, followed by CBT principles (n = 4, 10.8%) addressing psychological distress and anxiety management. Other prominent frameworks included Social Cognitive Theory for health behaviour change, mindfulness-based approaches drawing upon Mindfulness-Based Stress Reduction programmes, positive psychology interventions utilising the PERMA model, and specialised theories such as the Theory of Planned Behaviour for help-seeking interventions, Attachment Theory for parent-child programmes, and Social Identity Theory for stigma reduction. Technology-enhanced interventions incorporated frameworks such as the mHealth user engagement pyramid, whilst family-based programmes drew upon Systems Therapy and parent-child interaction theories, reflecting the evidence-based approaches required to address the multifaceted nature of mental health promotion across cognitive, behavioural, social, and family system domains.

#### Programme delivery and implementation.

The programmes were delivered by clinicians (n = 17, 45.9%) and the rest (n = 20, 54.1%) by educators, coaches, researchers or other trained personnel, with 4 programmes (10.8%) delivered through mobile apps and 8 (21.6%) through online mode whilst the rest (n = 25, 67.6%) were conducted through face-to-face sessions. Programme duration ranged from a single day to a year, with the majority lasting for extended periods with multiple sessions. Only 13 studies (35.1%) tailored their programmes to population characteristics, setting requirements, intended outcomes, and cultural contexts. Whilst 26 programmes (70.3%) reported no modifications, others (n = 11, 29.7%) adapted during the course of the study to address recruitment challenges and pandemic-related constraints. Moderate to high adherence was noted for most programmes (TIDieR Checklist is indicated in **S6 Table**).

#### Mental health outcomes and direction of evidence.

The programmes measured a comprehensive range of mental health outcomes across four main domains: psychological and mental wellbeing (most frequently assessed), emotional states (depression, anxiety, stress), cognitive functioning (attention, memory, neuropsychological performance), and behavioural indicators. Additional outcome categories included parenting-related measures (parental self-efficacy, confidence, postnatal depression, parent-child bonding), mental health literacy (depression literacy, help-seeking attitudes, stigma reduction), and specialised outcomes (mindfulness, self-compassion, burnout, compassion satisfaction, quality of life, social connectedness, sleep quality, test anxiety). Seven studies (18.9%) employed qualitative measures and the rest employed validated questionnaires to capture the outcome. Thirty-five (94.6%) of these programmes showed positive results.

#### Comparison of characteristics of different programmes.

A comparison of characteristics between the 4 groups showed that the programmes were mainly targeting those with mental health and neurological conditions and healthy populations (n = 37 each, 34.9%), while physical health conditions received moderate attention (n = 24, 22.6%) and neurodevelopmental and learning conditions were relatively underrepresented (n = 8, 7.5%). Key differences emerged in interventional approaches. Programmes for mental health and neurological conditions emphasised management and treatment interventions (n = 16, 43.2%), those for physical health conditions similarly focused on management approaches (n = 9, 37.5%) with limited prevention components, whereas neurodevelopmental programmes emphasised educational initiatives (n = 5, 62.5%). Programmes for healthy populations demonstrated the most diverse combination emphasising education and psychoeducational interventions (n = 14, 37.8%) and prevention and early intervention (n = 13, 35.1%). Populations varied markedly across categories, with neurodevelopmental programmes serving younger populations (75% children, 12.5% children/adolescents) in educational settings, physical health programmes predominantly included adults (n = 19, 79.2% including diverse age groups) in healthcare settings (n = 20, 83.3%), mental health and neurological programmes showed mixed age distribution with particular emphasis on adults and older adults (n = 30, 81.1% including overlapping age groups), and healthy population programmes demonstrated the broadest age representation across young adults alone (n = 11, 29.7%), older adults alone (n = 10, 27.0%) and adults alone (n = 9, 24.3%), with greatest community-based delivery (n = 16, 43.2%) and technology integration. Theoretical foundations were strongest in neurodevelopmental, and healthy population programmes, while outcomes varied from universal success in neurodevelopmental programmes to more modest results in physical health programmes (75.0%). These differences highlight distinct approaches and reveal critical population gaps, particularly the absence of school-aged student programmes for mental health promotion and limited workplace mental health interventions across all categories, despite these being crucial demographic groups in Singapore’s context. Additionally, mental wellbeing programmes for paediatric populations with physical health conditions were underrepresented, as were programmes targeting parents or caregivers of children with physical health conditions, highlighting significant gaps in addressing the mental health needs of these vulnerable populations.

#### Depth of evidence and knowledge gaps.

The evidence base predominantly consisted of primary data, whilst government reports largely described programmes from a policy perspective without detailed implementation insights. Reports lacking evidence or reviews were systematically excluded. Although quantitative outcomes were well-documented across studies, there was a critical lack of qualitative data capturing user experiences and programme satisfaction. Even when primary articles demonstrated implementation feasibility, they failed to adequately capture user perspectives. This created a significant gap in understanding the barriers and enablers of programme implementation in Singapore’s unique context. This limitation hampers the assessment of programme acceptability at both population and healthcare system levels.

Additionally, a major methodological weakness is evident in the research design, with only 25 RCTs among all studies, undermining the robustness of evidence. Programmes emphasised improving mental health through multiple approaches: reducing depressive symptoms, anxiety, improving cognition and managing mental health conditions in healthcare and community settings, prevention and health promotion and education and psychoeducational approaches to mental health. This focus revealed a balanced approach targeting multiple domains of health promotion. Nonetheless, the programmes under each domain lacked diversity. The same programmes were implemented in different populations by many studies. Given the rising prevalence of mental health conditions, new programmes that meet the changing needs of the population need to be designed and evaluated.

A significant oversight is the absence of programmes targeting school-aged students to promote help-seeking behaviours and improve mental wellbeing. This gap is particularly concerning given the crucial developmental stage and the increasing mental health challenges faced by youth in educational settings. Similarly, workplace mental wellbeing, despite being a critical aspect of adult mental health and productivity, was not addressed in any of the reviewed articles, representing another substantial gap in the literature.

Some programmes addressed mental health outcomes in individuals with physical disorders, focusing on coping, symptom management of depression and anxiety, and overall wellbeing. However, significant gaps remain in enhancing help-seeking behaviours among the general public. This is particularly crucial considering Singapore’s multi-ethnic population and unique cultural contexts. A substantial limitation emerges in the translation of research to practice: despite positive outcomes in experimental settings, there is insufficient evidence of successful implementation or sustainability in real-world community settings. The uncertainty about programme continuation beyond the research phase creates a concerning disconnect between research findings and practical community implementation. Additionally, digital mental health and artificial intelligence are important topics with immense public health potential. These elements were underrepresented in programmes, especially the youth-focused ones.

Several critical areas remain notably under-addressed. Suicidality and self-harm, despite their significance, were not targeted by any programmes. Whilst considerable attention was given to older adults in response to the ageing population, youth mental health programmes programmes are limited, need resourcing, and require substantial attention. Furthermore, although most studies employed psychometrically sound measures, there is a pressing need for culturally validated assessment tools specific to Singapore’s population. The substantial body of research focusing on older adults, while aligned with Singapore’s demographic challenges, warrants a systematic review to analyse the evidence on programmes improving mental health outcomes in older adults. Accumulating evidence demonstrates that environmental factors, including climate change, natural disasters, pandemics and urban design, significantly affect population mental health [128–130]. This highlights the paramount importance of developing interventions to build community resilience. However, these programmes were notably absent from the current research landscape. Lastly, the feasibility of population-level implementation requires economic evaluation to understand the cost-effectiveness of different approaches and how to make the economic case for investing in population mental health interventions, which is a significant limitation in current articles on programmes targeting mental health outcomes in Singapore.

## Discussion

The scoping review aimed to capture programmes promoting mental health outcomes in Singapore’s population to understand the literature extent on various programmes targeting different age groups, and knowledge gaps to guide future research. One hundred and six articles were included, encompassing programmes targeting mental health outcomes in those with mental disorders, physical conditions, neurodevelopmental and learning disabilities, and healthy individuals. Whilst many programmes targeted older adults, others focused on adults and adolescents. There was a dearth of articles targeting key areas such as youth-specific suicide prevention, participants’ experiences, feasibility of expansion beyond the experimental stage, resilience and policy implications.

The publication timeline showed an increasing trend from 2013 onwards, coinciding with the NMHB plan and Community Mental Health Masterplan launched in 2012 [15]. These policies aimed to improve mental health through early detection and treatment, with more focus on community mental health. The policy paved the way for active community outreach efforts through various partners to understand the problem and design strategies to combat the emerging crisis. The focus areas identified (e.g., depression, anxiety, dementia) coincide with the mental health outcomes targeted by the programmes included in the current review. The policy also led to multiple research projects examining the nation’s mental health status to understand areas for intervention.

For example, the Wellbeing of the Singapore Elderly study (WiSE) launched in 2013 examined the prevalence of dementia and depression in Singapore and noted that 1 in 10 residents had dementia [131]. Following the study, dementia-friendly communities were implemented for early detection and management of dementia at the community level. Depression and anxiety have been recognised as major population health threats, with the Singapore Mental Health Study (SMHS) showing a lifetime prevalence of 13.9% for at least one mood, anxiety, or alcohol use disorder at the population level, higher than the 12% observed in SMHS2012. Depression alone had a prevalence of 6.3%, with significant differences noted for generalised anxiety disorder between SMHS2010 and 2016 [11].

Notably, a large proportion of programmes focused on depressive symptoms in hospital and community settings, emphasising management, treatment, and continued care. This focus aligns with the high prevalence of depression in Singapore, reported at 6.3% in the Singapore Mental Health Study [114]. Whilst many programmes demonstrated positive outcomes in controlled settings, their translation to broader community implementation remained limited. This gap between efficacy in research settings and real-world implementation is a recognised challenge in mental health interventions [132]. The limited community-level implementation could be attributed to several factors, primarily the lack of information on users’ perceptions and experiences. Understanding user perspectives is crucial for programme sustainability, as highlighted by implementation science frameworks [133]. User acceptance and engagement are key determinants of programme success, particularly in mental health interventions where stigma and cultural beliefs can significantly influence help-seeking behaviours and treatment adherence [134]. Additionally, sustainable implementation requires consideration of local context, resources, and capacity building [135], aspects that were often underexplored in the existing programmes. Given the changing mental health landscape globally, programmes that target areas beyond the current focus of the Community Mental Health (CMH) are needed.

The programmes included those developed and implemented by healthcare organisations for specific patient populations (such as EPIP, guidance clinics for children, and GP partnership programmes), to innovative mobile app-based games used in clinical settings. Additionally, programmes incorporated dance, music, other arts, mindfulness, memory clinics, care coordination programmes, and psychoeducation to improve mental health outcomes across various disease domains by promoting recovery and teaching essential coping skills. These programmes also aimed to foster self-efficacy in the target population. Enns et al., [17] summarised evidence on interventions targeting population mental health outcomes and observed that programmes mainly focused on depression, anxiety, stress, quality of life, and overall wellbeing, similar to our findings. The content shared similarities with overlapping components of psychoeducation, psychosocial elements, and overall wellbeing. What was lacking in the Singapore setting was a focus on academic development for students, parenting skills, and workplace mental health programmes. Enns et al. noted that none of the programmes in their study focused on environmental impacts on residents’ mental health, similar to the current review. Das and colleagues [136], in their umbrella review, summarised evidence on youth-targeted programmes and noted a focus on school-based, community-level, digital, and individual-based interventions mainly targeting depression and anxiety. Whilst these programmes shared elements with those in our review, they also addressed eating behaviours, suicide prevention, and knowledge – areas lacking in Singapore. Singapore has a suicide rate of 6.17 per 100,000 residents [137], which persists as a leading cause of death among youth. Given the importance of this topic, literature on programmes targeting suicide prevention and related knowledge is currently lacking. Studies have identified the importance of workplace interventions in mental and physical health promotion with many workplaces adopting various policies and programmes in this regard [138–139]. Given the significant proportion of time spent at work and its impact on mental wellbeing, there is a pressing need for evidence-based workplace mental health programmes in Singapore.

We have noted only limited evidence on early detection and prevention in youth. Recent studies among youth in Singapore have evidenced that 14.9% and 27% of youth experience severe depressive and anxiety symptoms respectively [140]. The lifetime prevalence of non-suicidal self-injury among youth was noted to be 25% [141]. These findings emphasise the need for more intensive youth-specific preventive programmes at the community level. Similarly, chronic diseases have a significant impact on the mental health of the population, with evidence showing that depressive symptoms are common among those with chronic conditions [7]. The research landscape in Singapore has begun to focus on this relatively underexplored area for intervention, which is a positive step in the right direction.

The strength of the scoping review includes the use of established JBI methodology, and the breadth of search strategy used. Despite the systematic search, it is possible that some articles might have been missed. Whilst the review focused on articles published in Singapore, which could be seen as limiting the generalisability of the findings, it offers valuable insights for other Asian countries. The findings are particularly relevant as they showcase evidence-based programmes and interventions in a multiethnic Asian population, providing potential models that could be adapted and implemented in similar Asian contexts. The study population’s ethnic diversity (predominantly Chinese, Malay, and Indian) mirrors the demographic composition found across many Asian countries, making the interventions and outcomes potentially more culturally relevant and adaptable compared to Western studies. This allows other Asian countries to learn from Singapore’s experiences and potentially adapt successful programmes to their local contexts whilst considering their specific cultural and healthcare system characteristics.

This scoping review identified critical gaps in Singapore’s mental health programme landscape. These include underrepresentation of youth-focused interventions despite rising mental health challenges in this population, limited workplace mental health programmes and suicide prevention initiatives, lack of school-based mental health promotion, and limited research on environmental impacts on psychological wellbeing in Singapore’s urban context. These gaps highlight priority areas requiring immediate policy attention and resource allocation to ensure comprehensive mental health coverage across all population groups and settings. Future research should prioritise developing and evaluating programmes to address these gaps, while incorporating user experiences and perspectives for sustainable and impactful programmes.

The substantial success rate (90.6%) of mental health programmes regardless of setting provides a compelling case for clinicians and policymakers to implement them for those who will benefit from these interventions. These programmes demonstrate significant value for healthcare systems by reducing symptom burden, improving functional capacity, decreasing healthcare utilisation through better self-management, and enhancing quality of life across diverse populations, ultimately contributing to more efficient resource allocation and improved patient outcomes.

However, critical policy action is urgently required to address significant gaps including the absence of school-based mental health programmes, workplace interventions, and programmes targeting suicidality, with immediate investment needed in youth mental health initiatives. Policymakers must prioritise sustainable funding mechanisms for scaling successful interventions beyond research settings, mandate economic evaluations to establish cost-effectiveness and return on investment, and strengthen research methodologies given the limited number of clinical trials. Policy frameworks should facilitate the translation of research findings into routine practice.

## Conclusion

Mental health is a fundamental component of overall wellbeing, serving as a critical balance point that influences physical, social, and emotional functioning. Our review revealed a substantial increase in locally published research since the implementation of the NMHB and CMH Masterplan, with active research participation across all healthcare clusters in Singapore.

This scoping review identified 106 mental health programmes implemented across Singapore, with 90.6% showing positive outcomes across four main categories. The evidence revealed strong institutional engagement across Singapore’s three major healthcare clusters, with programmes successfully implemented across healthcare, community, and educational settings using diverse delivery modalities. Whilst there was robust evidence for programmes targeting older adults and adults, there was notably less emphasis on preventive strategies and health promotion initiatives among youth, adolescents and children. This imbalance is particularly concerning given the rising mental health challenges among youth and working young adults. The limited research on crucial areas such as suicide prevention programmes, workplace mental health initiatives, and the impact of built environments on mental health represents a significant gap, especially considering Singapore’s urbanised setting, workplace stress levels, and youth suicide rates.

Future research should prioritise addressing knowledge gaps identified in the review. Key priorities include incorporating user experiences and perspectives, developing and evaluating preventive interventions particularly for youth mental health, and investigating environmental influences on psychological wellbeing. Additional priorities for future research include creating sustainable workplace mental health programmes, establishing evidence-based suicide prevention strategies, evaluating the long-term effectiveness of community-based interventions, and assessing the cultural appropriateness and adaptability of existing programmes. These priorities will help ensure that mental health interventions in Singapore are not only effective in controlled settings but also sustainable and impactful in real-world applications across diverse population groups.

## Supporting information

S1 Table Preferred Reporting Items for Systematic reviews and Meta-Analyses extension for Scoping Reviews (PRISMA-ScR) Checklist. The table shows the list of PRISMA items and corresponding page numbers.
(DOCX)

S2 Appendix Search strategy. (DOCX)

S3 Table Study characteristics: Characteristics of all included studies. (DOCX)

S4 Table Study characteristics based on TIDieR checklist list: Programmes targeting mental health outcomes in those with mental health and neurological conditions. (DOCX)

S5 Table Study characteristics based on TIDieR checklist list: Programmes targeting mental health outcomes in those with physical health conditions. (DOCX)

S6 Table Study characteristics based on TIDieR checklist list: Programmes targeting mental health outcomes in those with neurodevelopmental and learning conditions. (DOCX)

S7 Table Study characteristics based on TIDieR checklist list: Programmes targeting mental health outcomes in healthy population. (DOCX)
