# Supplementary material for: Mapping programmes for mental health promotion in Singapore: A scoping review
Source: PLoS One. 2026 Apr 28;21(4):e0347518. doi: 10.1371/journal.pone.0347518 (PMC13124008; doi:10.1371/journal.pone.0347518)
Supplement: S2 Appendix — (DOCX) [file pone.0347518.s002.docx]

**Search strategy for Medline**

1 (mental health or (mental health or Mental Health Services or Mental health program or Mental health treatment or Mental health care or Mental health screening or Mental health promotion or Mental health assessment or mental health counseling or Mental health review or mental health education or Mental Health Associations or community mental health or Child Mental Health or mental health organization or mental health center or mental health worker or Mental Health Personnel or Teen Mental Health or Seniors' Mental Health or mental health evaluation or Mental Health Consultation)).mp. [mp=title, book title, abstract, original title, name of substance word, subject heading word, floating sub-heading word, keyword heading word, organism supplementary concept word, protocol supplementary concept word, rare disease supplementary concept word, unique identifier, synonyms, population supplementary concept word, anatomy supplementary concept word]

2 psychological.mp. or (Psychiatry/ or Psychology/) [mp=title, book title, abstract, original title, name of substance word, subject heading word, floating sub-heading word, keyword heading word, organism supplementary concept word, protocol supplementary concept word, rare disease supplementary concept word, unique identifier, synonyms, population supplementary concept word, anatomy supplementary concept word]

3 (mental adj3 health promot$).mp. [mp=title, book title, abstract, original title, name of substance word, subject heading word, floating sub-heading word, keyword heading word, organism supplementary concept word, protocol supplementary concept word, rare disease supplementary concept word, unique identifier, synonyms, population supplementary concept word, anatomy supplementary concept word]

4 (mental health adj2 outcome$).mp. [mp=title, book title, abstract, original title, name of substance word, subject heading word, floating sub-heading word, keyword heading word, organism supplementary concept word, protocol supplementary concept word, rare disease supplementary concept word, unique identifier, synonyms, population supplementary concept word, anatomy supplementary concept word]

5 Positive mental health.mp. or positive psycholog$.ab.

6 (Mental adj2 well?being).mp. [mp=title, book title, abstract, original title, name of substance word, subject heading word, floating sub-heading word, keyword heading word, organism supplementary concept word, protocol supplementary concept word, rare disease supplementary concept word, unique identifier, synonyms, population supplementary concept word, anatomy supplementary concept word]

7 (psycholog$ adj3 well?being).mp. [mp=title, book title, abstract, original title, name of substance word, subject heading word, floating sub-heading word, keyword heading word, organism supplementary concept word, protocol supplementary concept word, rare disease supplementary concept word, unique identifier, synonyms, population supplementary concept word, anatomy supplementary concept word]

8 mental disorder$.ab.

9 mental$ ill$.ab.

10 (psychiatric disorder or psychiatric ill$).ab.

11 ((mental adj4 treatment outcome) or (mental adj4 treatment adherence) or (mental adj4 treatment compliance)).mp.

12 (mental adj2 wellness).ab. or suicid$.ti,ab.

13 1 or 2 or 3 or 4 or 5 or 6 or 7 or 8 or 9 or 10 or 11 or 12

14 exp "Quality of Health Care"/og, sn [Organization & Administration, Statistics & Numerical Data]

15 exp "Quality of Life"/ or (quality adj2 care).mp. [mp=title, book title, abstract, original title, name of substance word, subject heading word, floating sub-heading word, keyword heading word, organism supplementary concept word, protocol supplementary concept word, rare disease supplementary concept word, unique identifier, synonyms, population supplementary concept word, anatomy supplementary concept word]

16 exp Health Status Indicators/ or exp Public Health/ or exp Population Health/ or exp Population Surveillance/ or exp Health Status/

17 (community adj3 mental health).mp. [mp=title, book title, abstract, original title, name of substance word, subject heading word, floating sub-heading word, keyword heading word, organism supplementary concept word, protocol supplementary concept word, rare disease supplementary concept word, unique identifier, synonyms, population supplementary concept word, anatomy supplementary concept word]

18 (school$ or student$).ab.

19 ((octave or REACH) adj2 service*).ab. or ((psychosis adj2 intervention program$) or EPIP or "Community Health Assessment Team").ti,ab.

20 "Community Health Assessment team".mp. [mp=title, book title, abstract, original title, name of substance word, subject heading word, floating sub-heading word, keyword heading word, organism supplementary concept word, protocol supplementary concept word, rare disease supplementary concept word, unique identifier, synonyms, population supplementary concept word, anatomy supplementary concept word]

21 ((program$ adj3 health) or (suicide adj2 prevent$) or intervention*).mp. [mp=title, book title, abstract, original title, name of substance word, subject heading word, floating sub-heading word, keyword heading word, organism supplementary concept word, protocol supplementary concept word, rare disease supplementary concept word, unique identifier, synonyms, population supplementary concept word, anatomy supplementary concept word] 1481076

22 14 or 15 or 16 or 17 or 18 or 19 or 20 or 21

23 exp Adult/ or exp Young Adult/ or exp Adult Children/

24 exp aged/ or exp middle aged/ or exp caregivers/ or exp men/ or exp minors/ or exp parents/ or exp patients/ or exp population groups/ or exp prisoners/ or exp students/ or exp women/ or exp "health care (non mesh)"/

25 exp Aging/ or older adult*.mp. or exp "Aged, 80 and over"/

26 Elderly.ab.

27 Child Psychiatry/ or child.mp. or *Child Health Services/ or *Child Behavior Disorders/ or exp Child Health/ or *Child Behavior/ or *Psychology, Child/ or exp Child$/

28 exp Adolescent Psychiatry/ or exp Adolescent Health/ or Psychology, Adolescent/ or Adolescent*.mp. or exp Adolescent Health Services/ or exp Adolescent/ or exp Adolescent, Institutionalized/ or exp Adolescent Medicine/

29 (general population or community or resident* or (treatment adj2 seek$) or public).ab.

30 23 or 24 or 25 or 26 or 27 or 28 or 29

31 exp Singapore/ or Singapore.mp.

32 13 and 22 and 30 and 31

33 limit 65 to (english language and humans and yr="2000 -Current")
