# Supplementary material for: Mapping programmes for mental health promotion in Singapore: A scoping review
Source: PLoS One. 2026 Apr 28;21(4):e0347518. doi: 10.1371/journal.pone.0347518 (PMC13124008; doi:10.1371/journal.pone.0347518)
Supplement: S3 Table — (DOCX) [file pone.0347518.s003.docx]

**S3 Table: Study characteristics of included studies**

| **Author** | **Year** | **Institution** | **Population** | **Setting** | **Gender** | **Intervention/Programme** | **Focus of the programme  (what condition)** | **Sample Size** | **Mental Health Outcomes** | **Measures** | **Direction** |
| --- | --- | --- | --- | --- | --- | --- | --- | --- | --- | --- | --- |
| Aloweni et al., 2022 | 2022 | SGH | Adults | Hospital | Male=13 Female=31 | Mindfulness Therapy | Caregiver stress and anxiety | N=44 | 1. Stress 2. Anxiety 3. QOL 4. Caregiver response | 1. PSS 2. STAI 3. SF-36, KDQOL-SF 4. CRA | Positive outcome |
| Ang, 2018 | 2018 | TTSH | NR | Hospital | NR | Suicide management programme | Suicide | N=7 | Completed suicides | 1. Actual numbers | Positive outcome |
| Bernard-Opitz et al., 2004 | 2004 | NUS | Children | Hospital | NR | Traditional behavioural approaches and natural play interventions | Autism | N=8 | 1. Autism scores | 1. PL-ADOS, SPT | Positive outcome |
| Bos at al., 2018 | 2018 | KKH | Adults | Hospital | Male=3 Female=39 | Neonatal discharge programme (parentcraft teaching) | Psychological distress | N=42 | 1. Parental efficacy 2. Psychological distress | 1. PSES 2. DASS-21 | Positive outcome |
| Chan et al., 2013 | 2013 | NUS | Older adults | Community | Male=5 Female=21 | Life storybook | Depression | N=26 | 1. Depression | 1. GDS-15 | Positive outcome |
| Chan et al., 2014 | 2014 | NUS | Older adults | Community | Male=6 Female=23 | Lifestory reviews | Depression | N=29 | 1. Depression | 1. GDS-15 | Positive outcome |
| Chan et al., 2015 | 2015 | TTSH | Older adults | Community | NR | Driver Retirement Programme | Wellbeing | N=15 | 1. Depression 2. Self-efficacy 3. Goal satisfaction 4. HrQOL | 1. GDS 2. Self-efficacy questionnaire 3. Individual goal satisfaction 4. SF-36 v2 | Positive outcome |
| Chandran et al., 2024 | 2024 | NUH | Adults, older adults | Hospital | Female=70 | Multidimensional rehabilitation programme | Breast cancer | N=70 | 1. Cognitive function 2. Fatigue 3. QOL | 1-2. FACT-G 3. EORTC QLQ-C30 | Negative outcome |
| Chew et al., 2025 | 2025 | TTSH | Older adults | Community | Male=35 Female=115 | ADL+ | Cognitive decline | N=150 | 1. Cognitive function 2. Cognitive decline 3. Depressive symptoms 4. QOL 5. Attention 6. Processing speed  7. Memory 8. Executive function | 1. mCMMSE 2. AD8 3. GDS 4. EQ-5D-5L, EQ VAS 5. CTT 1 6. Symbol Search, SDMT 7. Logical Memory sub-test from the WMS-IV 8. CTT 2, animal category | Positive outcome |
| Chong et al., 2024 | 2024 | NTFGH | Adults, older adults | Community | Male=3 Female=16 | Get Well, Live Well programme | HL, health behaviours | N=19 | 1. Perceptions and experiences of the Get Well, Live Well programme | 1. Semi-structured interviews | Positive outcome |
| Guna et al., 2022 | 2022 | Sunlove Nursing Home | Older adults | Nursing Home | Male=49 Female=25 | Biography and life storybook (BLSB) | Depression | N=74 | 1. Depression 2. Life satisfaction 3. QOL | 1. GDS-15 2. LSIA 3. QoL-NHR | Positive outcome |
| Fitriya., 2021 | 2021 | DAS | Children | School | NR | English Main Literacy Programme | Dyslexia | N=1343 | 1. Progress of students (reading writing and learning) | Curriculum-Based Assessment | Positive outcome |
| Fitriya., 2022 | 2022 | DAS | Children | School | NR | English Main Literacy Programme | English literacy | N=1280 | Mean percentage scores for reading, writing and spelling | Curriculum-Based Assessment | Positive outcome |
| Gan et al., 2021 | 2021 | MSF | Young adults | Community | Male=107 Female=13 | Functional Family Therapy | Wellbeing | N=120 | 1. Mental wellbeing 2. Perceived family functioning | 1. YOQSR2.0 2. FAD‐GF | Positive outcome |
| Griva et al., 2019 | 2019 | NTU | Adults, older adults | Hospital | Male=21 Female=23 | Combined Diabetes and Renal Control Trial (C-DIRECT) intervention | Psychosocial functioning | N=44 | 1. Anxiety 2. Depression 3. Emotional wellbeing 4. Role emotional 5. Social functioning 6. Mental composite summary 7. Cognitive function 8. Quality of social interaction 9. Sleep 10. Social support 11. Positive and active engagement in life 12. Constructive attitudes and approaches 13. Self-monitoring and insight 14. Social integration and support 15. Emotional wellbeing | 1-2. HADS 3-10. KDQOL 11-15. HEIQ | No effect |
| He et al. 2015 | 2015 | NUS | Adults, children | Hospital | Parents Male=17 Female=78 | Therapeutic play intervention | Anxiety | N=190 | 1. Perioperative anxiety | 1. SAS-C, SAS-A, qualitative interviews | No effect |
| Hoong et al., 2023 | 2023 | JurongHealth Campus | Older adults | Community | Male=146 Female=369 | Chronic Disease Self-Management Programme (CDSMP) | Health outcomes | N=461 | 1. Self-efficacy 2. Social activities 3. Stress 4. Sleep 5. Cognitive symptom management 6. Depression | 1. CDSES 2. Social/role Activities Limitations Scale 3-4. VNS 5. Cognitive Symptom Management Behaviour Change Scale 6. PHQ-8 | Positive outcome |
| Huang et al., 2017 | 2017 | IMH | Adults, older adults | Community | Male=135 Female=386 | General Practitioner Partnership Programme (GPPP) | Mental disorders | N=521 | 1. Referral acceptance rate 2. Readmission rate | - | Positive outcome |
| Jiao et al., 2019 | 2019 | NUS | Adults | Community | Female=204 | Postnatal psychoeducational intervention for first-time mothers | PND | N=204 | 1. Maternal parental self-Efficacy 2. Social support 3. Psychological wellbeing | 1. PMPSE 2. PICSS-modified 3. EPDS & HADS-A | Positive outcome |
| Kasmani et al., 2018 | 2018 | IMH | Young adults, adults, older adults | Hospital and Community | NR | Multilevel Bidirectional (MLB) Care Coordination Model | Mental disorders | N=1065 | Acceptance rates for referrals to CBPRFs, rejection rates | Annual average acceptance and rejection rates for referrals to CBPRFs | Positive outcome |
| Keng et al., 2022 | 2022 | Duke-NUS | Adults | Online | Female=72 | Headspace mobile app-based mindfulness practice | Psychological distress | N=80 | 1. Depression 2. Anxiety 3. Fear of COVID-19 4. PTSD symptoms 5. Personal wellbeing 6. Compassion satisfaction 7. Burnout 8. Perceived sleep quality 9. Trait mindfulness 10. Self-compassion 11. Digit-span forward 12. Digit-span backward | 1-2. DASS-21 3.FCV-19S 4. PCL-C 5. Personal Well-being Index 6-7. ProQoL - Burnout and Compassion Satisfaction subscales 8. PSQI 9. FFMQ 10. SCS 11-12. DGS – Forward and Backward from WAIS | Positive outcome |
| Khine et al., 2020 | 2020 | NUS | Older adults | Community | Male=33 Female=90 | Mindful Awareness Programme (MAP) | Cognitive function | N=123 | 1. Cognitive function 2. Psychological wellbeing | 1. Cognitive Function:  (a) CDR (b) RAVLT (c) DGS (d) BDT (e) CTT (f) Semantic Fluency Span  2. Psychological wellbeing: (a) GDS (b) GAI | Positive outcome |
| Kit et al., 2019 | 2019 | NTU | Children | School | Male=18 Female=15 | Live Chat online counselling (Ask iZ Master) via iZ Hero Challenge portal | Help-seeking | N=33 | 1. Qualititative experiences | 1. Qualitative interviews | Positive outcome |
| Klainin-Yobas et al., 2016 | 2016 | NUS | Adults | Hospital | Male=21 Female=34 | S-Manage Programme | Stress management | N=55 | 1. Objective stress 2. Subjective stress 3. Psychological health | 1. Skin temperature, SIgA 2. PSS 3. GHQ | Positive outcome |
| Koh et al., 2020 | 2020 | CGH | Older adults | Community | Male=13 Female=22 | Person-centred creative dance intervention | Dementia care | N=35 | 1. Well-being 2. QOL 3. Caregiver stress | 1. DCM 2. QOL-AD 3. ZBI | Positive outcome |
| Lai et al., 2019 | 2019 | NUP | Older adults | Primary Care (Polyclinic) | First visit Male=90 Female=176  Review visit NR | Memory Clinic | Dementia care | N=489 | 1. Management of dementia cases 2. Presence of behavioural and psychological symptoms of dementia 3. Caregiver needs | 1. Percentage of cases managed in primary care 2. Percentage with behavioural/psychological symptoms 3. Percentage of caregivers needing support | Positive outcome |
| Lee at al., 2013 | 2013 | Duke-NUS | Older adults | Community | Male=12 Female=19 | Brain-Computer Interface Based Cognitive Training System | Cognitive function | N=31 | 1. Cognitive function | 1. RBANS | Positive outcome |
| Lee at al., 2015 | 2015 | Duke-NUS | Older adults | Community | Male=12 Female=27 | Brain-Computer Interface Based Cognitive Training System | Cognitive function | N=39 | 1. Cognitive function | 1. RBANS | Positive outcome |
| Lee et al., 2003 | 2003 | IMH | Adolescents | Hospital | Male=1372 Female=1164 | Child Guidance Clinic | Mental disorders | N=2536 | 1. Utilisation of mental health services | 1. Number of new cases, referral sources | Neutral outcome |
| Lee et al., 2016 | 2016 | KKH | Adults | Hospital | Male=0, Female=307 | Postnatal depression screening and clinical intervention | PND | N=307 | 1. PND 2. Functioning 3. QOL | 1. EPDS  2. GAF  3. EQ VAS | Positive outcome |
| Lee et al., 2020 | 2020 | Duke-NUS | Adolescents | Community | Male=100 Female=211 | Social norm-based intervention for physical activity | Obesity | N=311 | 1. QOL 2. Depression 3. Social support for exercise 4. Physical activity self efficacy 5. Physical activity enjoyment | 1. Peds-QL 2. AADS 3. SSE 4. PASES 5. PACES-8 | No effect |
| Lee et al., 2024 | 2024 | NUS | Young adults | University | Male=52 Female=171 Nonbinary=1 Other=1 | Intellect | Subclinicial Obsessive-Compulsive Disorder | N=225 | 1. OCD symptom severity 2. Psychological distress 3. Perfectionism | 1. OCI-R 2. DASS-21 3. FMPS | Positive outcome |
| Leong et al., 2022 | 2022 | CGH | Older adults | Community | Male=5 Female=10 | Intergenerational Programme | Wellbeing | N=15 | 1. Emotional responses, attitudes and perceptions towards younger generation | 1. Thematic analysis | Positive outcome |
| Leow et al. 2015 | 2015 | NSC | Adults, older adults | Hospice, clinic | Male=26 female=54 | Caring for the Caregiver Programme | Caregiver support | N=80 | 1. Stress and depression 2. QOL 3. Social support 4. Closeness between caregivers  5. Self-efficacy 6. Postive gains of caregiver 7. Awareness of caregiver towards ACP | 1. DASS 2. CQOLC 3. SSQ 4. General Closeness Scale 5. SCSES 6. RC Scale 7. Self-reported questionnaire | Positive outcome |
| Li et al. 2014 | 2014 | NTU | Young adults | University | Male=18 female=24 | Disability awareness programme | Attitude towards intellectual disability | N=42 | 1. Attitude towards disability | 1. CLAS, Reflection journals | Positive outcome |
| Lim et al., 2012 | 2012 | IMH | Children | Hospital | Male=16 Female=4 | The BCI-based Attention Training Game System | ADHD | N=20 | 1. ADHD symptoms 2. ADHD severity | 1. ADHD-RS 2. BASM | Positive outcome |
| Lim et al., 2014 | 2014 | NUS | Adults,older adults | Hospital | Male=5 Female=13 | Relaxation intervention | Psychological distress | N=18 | 1. Stress 2. Relaxation 3. Self-efficacy 4. Anxiety | 1. NSRS, ST 2. PRS 3. SES 4. STAI | Positive outcome |
| Lim et al., 2019 | 2019 | SGH | NR | Hospital | Male=10 Female=3 | Support availability, Thinking positively with acceptance, Overcoming social stigma, Minimizing negative feelings, Analyzing self‐efficacy in stoma care (STOMA) psychosocial intervention programme | Coping | N=13 | 1. Attitude towards STOMA | 1. One to one interview | Positive outcome |
| Lim et al., 2019 | 2019 | SGH | Adults | Hospital | Male=33 Female=18 | STOMA programme | Coping | N=51 | 1. Anxiety 2. Depression 3. QOL 4. Length of stay | 1 and 2: HADS 3. EORTC QLQ-C29 4. Hospital records | No change |
| Lim et al., 2024 | 2024 | NUS | Young adults | School | NR | Educational | HL, health behaviours | N=131 | 1. Mental health knowledge 2. Mental health attitudes 3. Mental health practices | 1-3. 5-point Likert scale questions | Positive outcome |
| Lim et al., 2024 | 2024 | NTFGH | Older adults | Community | Male=112 Female=352 | "Wise and Well" programme | Health behaviours | N=464 | 1. Lifestyle goal attainment | 1. Goal Attainment Scale | Positive outcome |
| Loh et al., 2023 | 2023 | KKH | Adults | Hospital | Females=25 | Sure-mums intervention | PND and postnatal anxiety | N=25 | 1. Mother and baby bonding 2. Depression 3. Functioning | 1. PBQ  2. EPDS 3. GAF | Positive outcome |
| Low et al., 2013 | 2013 | IMH | Young adults, Adults, older adults | Hospital | NR | Assertive Community Treatment (ACT) | Severe mental illness | N=155 | 1. Number of admissions 2. Total number of hospital stay pre and post assertive community management | Medical record review | Positive outcome |
| Mahendran etal., 2015 | 2015 | NUS | Adults | Hospital | Male=43 Female=78 | Brief nurse-led psychosocial intervention program | Psychological distress | N=121 | 1. Distress 2. Anxiety 3. Depression 5. QOL | 1. Distress Thermometer 2-3: HADS 4. EQ VAS | Positive outcome |
| Merchant et al., 2024 | 2024 | NUS | Older adults | Primary Care | Male=87 Female=100 | Exercise and Cognitive Stimulation Therapy | Intrinsic capacity | N=187 | 1. Cognition 2. Psychological function | 1. MoCA 2. GDS-15 | Positive outcome |
| Metrat-Depardon and Teo, 2023 | 2023 | NTU | Young adults | School | Male=12 Female=22 | Happiness Mentoring Programme | Psychological wellbeing | N=34 | 1. Adolescent well being 2. Happiness 3. Feedback on the programme | 1 and 2. PERMA-Profiler questionnaire  with added items from EPOCH Measure of Adolescent Well-being, Children's Hope Scale, Gratitude Questionnaire and SWLS 3. Open ended questions on feedback | Positive outcome |
| Muckle and Lasikiewicz, 2017 | 2017 | JCU | Young adults | School | Male=11 Female=51 | Animal assisted activities | Wellbeing | N=62 | 1. Stress 2. Anxiety 3. Self-esteem | 1. PSS 2. STAI 3. SSES | Positive outcome |
| Neo et al., 2024 | 2024 | NCSS | Adults, older adults | Hospital | Male=65 Female=34 | Nurse-led telehealth programme | Palliative care | N=99 | 1. Emotional concerns | 1. IPOS | Positive outcome |
| Ng et al., 2018 | 2018 | NUS | Older adults | Community | Male=8 Female=72 | Horticultural therapy | Mental well being | N=59 | 1. Cognitive function 2. Depression 3. Anxiety 4. Psychological wellbeing 5. Social connectedness 6. Satisfaction with life | 1. MoCA 2. SDS 3. SAS 4. SPWB 5. FS 6. SWLS | Positive outcome |
| Ng et al., 2020 | 2020 | NUS | Older adults | Community | Male=149 Female=185 | Community-Based Early Psychiatric Interventional Strategy | Depression | N=334 | 1. Receipt of treatment and care 2. Depression 3. QOL | 1. Reports from GPs 2. SCID-I, GDS, HDRS, BDI 3. SF-12 | Positive outcome |
| Nyunt et al., 2009 | 2009 | NUS | Older adults | Community | Male=1922 Female=2711 | Community-Based Early Psychiatric Interventional Strategy | Depression | N=4633 | 1. Depressive symptoms  2.Treatment acceptance 3. Any psychiatric disorder 4. Mental health status | 1. GDS 2. Primary care self report 3. SCID 4. Self report | Positive outcome |
| Ong et al., 2019 | 2019 | IMH | Children | Hospital | Male=49 Female=23 | Mobile app: RegnaTales | Aggression | N=72 | 1. Aggression | 1. RPQ | Positive outcome |
| Ooi et al., 2008 | 2008 | IMH | Children | School | NR | Cognitive-behavioural therapy | Anxiety | N=6 | 1. Anxiety 2. Parental stress 3. Teachers' stress | 1. SCAS-C, SPAC-P, ACAS 2. PSI 3. ITS | Positive outcome |
| Pat-Horenczyk et al., 2015 | 2015 | MSF | Children | Childrens' Home | Male=33 Female=40 | Building Emotion and Affect Regulation (BEAR) programme | Emotional dysregulation | N=73 | 1. Coping 2. Distress | 1-2. Self report | Positive outcome |
| Ramazanu et al., 2021 | 2021 | PolyU | Adults Older adults | Hospital | Male=7 Female=7 | 3H (Hand, head and heart) intervention | Coping | N=14 | 1. Coping | 1. Interviews | Positive outcome |
| Rawtaer et al., 2015 | 2015 | NUH | Older adults | Community | Male=25 Female=76 | Pychosocial intervention (Tai Chi exercise, Art Therapy, Mindfulness Awareness Practice and Music Reminiscence Therapy) | Subsyndromal depression and anxiety | N=101 | 1. Depression 2. Cognition 3. Anxiety | 1. GDS, SDS 2. MMSE 3. GAI, SAS | Positive outcome |
| Saxena et al., 2018 | 2018 | NHG | Older adults | Primary Care (Polyclinic) | Male=91 Female=172 | Primary Care Dementia Clinic | Dementia care | N=263 | 1. QOL 2. Caregiver burden | 1. QOL-AD, EQ-5D-5L 2. ZBI | Positive outcome |
| Shah et al., 2015 | 2015 | IMH | Adults, older adults | Hospital | Male=6 Female=16 | VR-based stress management (VR DE-STRESS) programme | Stress management | N=22 | 1. Stress 2. Depression 3. Anxiety 4. Perceived relaxation 5. Knowledge on stress and stress management | 1-3. DASS-21 4. PRS 5. KSSMQ | Positive outcome |
| Shahwan et al., 2020 | 2020 | IMH | Young adults | University | Male=155 Female=235 | Anti-stigma intervention | Mental health stigma | N=390 | 1. Psychological openness 2. Help-seeking propensity 3. Indifference to stigma | 1-3. IASMHS | Positive outcome |
| Shorey et al., 2013 | 2013 | NUS | Adults | Community | Female=122 | Postnatal pychoeducation programme | PND | N=122 | 1. Maternal parental self-efficacy 2. Social support 3. PND | 1. PMPSE 2. PICSS 3. EPDS | Positive outcome |
| Shorey et al., 2015 | 2015 | NYP | Adults | Hospital | Female=18 | Postnatal psychoeducation programme | Postnatal care | N=18 | 1. Maternal confidence 2. Emotional wellbeing | 1-2. Semi-structured interviews | Positive outcome |
| Shorey et al., 2017 | 2017 | NUS | Adults | Hospital | Male=125 Female=125 | Home-but not Alone mobile health application | Postpartum parenting outcomes | N=250 | 1. Parental self-efficacy 2. Social support 3. Postnatal depression 4. Parenting satisfaction | 1. PES 2. PSSP 3. EPDS 4. WBPL- Satisfaction subscale | Positive outcome |
| Shorey et al., 2019 | 2019 | NUS | Adults | Community | Female=138 | Technology-based peer-support intervention programme | PND | N=138 | 1. PND 2. Postnatal anxiety 3. Loneliness 4. Perceived social support | 1. EPDS, PHQ-9 2. STAI 3. UCLA Loneliness Scale 4. PSSP | Positive outcome |
| Shorey et al., 2021 | 2021 | NUS | Older adults | Community | Male=9 Female=19 | Where-there-is-no-psychiatrist Integrated Personal Therapy (WIPT) | Subsyndromal depression and/or SS anxiety | N=28 | 1. Depression 2. Anxiety 3. Life satisfaction 4. Social connectedness (friendship) 5. QOL | 1. GDS 2. GAI 3. SWLS 4. FS 5. WHO-QoL-Old | No effect |
| Shorey et al., 2023 | 2023 | NUS | Adults | Hospital | Male=200 Female=200 | Supportive Parenting App (SPA) | Postnatal wellbeing | N=400 | 1. Postnatal depression  2. Anxiety 3. Parental bonding 4. Parental self-efficacy 5. Social support | 1. EPDS 2. STAI 3. PIBQ 4. PES 5. PSSP 6. WPBL | Positive outcome |
| Sia, A., et al., 2020 | 2020 | NUS | Older adults | Community | Male=14 Female=33 | Therapeutic horticulture programme | Psychological wellbeing | N=47 | 1. Happiness 2. Anxiety 3. Depression 4. Cognitive function  5. Sleep quality 6. Social connectedness and loneliness | 1. VAS 2. STAI 3. SDS 4. MMSE 5. PSQI 6. FS | Postive outcome |
| Sim et al., 2007 | 2007 | IMH | Young adults and adults | Hospital | Male=153 Female=125 | Early Psychosis Intervention Programme (EPIP) | Early psychosis | N=278 | 1. Severity of psychopathology 2. Level of insight 3. Functioning 4. QOL | 1. PANSS  2. SUMD  3. GAF  4. WHOQOL-BREF | Postive outcome |
| Sim et al., 2021 | 2021 | NUS | Adolescents, adults | Community | Adolescents Male=10   Adults Male=2 Female=8 | Gaming disorder intervention | Gaming disorder | N=21 | 1. Changes in gaming habits | 1. Qualitative interviews | Positive Outcome |
| Subramaniam et al., 2020 | 2020 | IMH | Young adults | University | Male=155 Female=236 | Anti-stigma intervention | Mental health stigma | N=390 | 1. Depression literacy 2. Personal stigma 3. Social distance | 1. D-Lit 2. DSS - Personal Stigma subscale 3. SDS | Positive outcome |
| Tan and Mankiewicz, 2024 | 2024 | NUS | Young adults | Community | Male=31 Female=137 | Brief video contact-based intervention | Mental health stigma | N=168 | 1. Social distancing attitudes 2. Tolerance/support for community care attitudes 3. Social restrictiveness attitudes 4. Prejudice and discrimination attitudes | 1-4. AMI-SG | Positive outcome |
| Tan et al., 2009 | 2009 | SGH | Adults | Hospital | Male=7 Female=32 | Group CBT programme | Chronic pain | N=39 | 1. Use of Unhelpful Strategies 2. Problem-Solving Ability 3. Self-efficacy 4. Positive Statements 5. Negative Statements 6. Fear of Harm 7. Pathophysiological Beliefs 8. Depression 9. Anxiety 10. Stress | 1-2. PSMC 3. PSEQ 4-5. PRSS  6-7. TSK 8-10. DASS | Positive outcome |
| Tan et al., 2015 | 2015 | IMH | NR | Prison | Male=50 | Psychiatric Housing Unit programme | Life skills | N=50 | 1. Engagement 2. Initiative 3. Concentration 4. Interpersonal skills 5. Communication | 1-5. Task Behavioural Scale | Positive outcome |
| Tan et al., 2017 | 2017 | IMH | Adults, older adults | Community | Male=19 Female=31 | Illness management and recovery programme | Mental disorders | N=50 | 1. Illness management and recovery 2. Psychotic symptoms 3. Functioning | 1. IMRS, number of admissions, length of stay  2. BRPS  3. GAS | Positive outcome |
| Tan et al., 2021 | 2021 | NUH | Adults | Hospital | Male=16 Female=24 | Virtual screen-based stress management programme (V-DESSERTS) | Stress management | N=40 | 1. Subjective stress 2. Objective stress 3. Relaxation levels 4. Knowledge on stress and medication management | 1. PSS, NSRS 2. Blood pressure, heart rate, skin temperature 3. PRS 4. KSMMQ | Positive outcome |
| Tan et al., 2021 | 2021 | IMH | Young adults | University | Male=155 Female=236 | Advancing Research Toward Eliminating Mental Illness Stigma (ARTEMIS) | Help-seeking, MHL | N=390 | 1. Recognition of depression 2. Help-seeking beliefs | 1-2. Vignettes | Positive outcome |
| Tan et al., 2022 | 2022 | Dementia Singapore Ltd | Older Adults | Community | Male=6 Female=15 | Arts and Dementia Programme | Wellbeing | N=21 | 1. Behavioural tendencies  2. Mood and engagement | 1-2. DCM | Positive outcome |
| Tan et al., 2022 | 2022 | SUTD | Children | Community | Male=20 Female=11 (drop outs unknown) | Lexicaid | Learning and psychosocial wellbeing | N=45 | 1. Writing competence 2. Reading competence 3. Spelling competence 4. General intelllectual ability | 1-4. SPPLD | Positive outcome |
| Tan et al., 2024 | 2024 | SHP | Adults | Primary Care | Male=11 Female=10 | OPTIMUM—Optimising care of Patients via Telemedicine In Monitoring and Augmenting their control of Diabetes Mellitus | Self management | N=21 | 1. Self-efficacy | 1. Semi-structured interviews | Positive outcome |
| Tan et al., 2024 | 2024 | Digital Dream Singapore | Children, adolescents | School | NR | Immersive Interactive Mixed Reality (IMR) educational intervention | Psyschosocial functioning | N=86 (students) N=10 (teachers) | 1. Mental wellbeing 2. Social skills 3. Teachers' work satisfaction 4. Teachers' sense of efficacy | 1. SWEMWBS 2. Social Skills Checklist 3. UWES 4. TSES | Positive outcome |
| Tay et al., 2022 | 2022 | IMH | Young adults | Online | Male=50, Female=124 | Online HOPE Intervention | Psychological wellbeing, MHL | N=174 | 1. Depression literacy 2. Anxiety literacy 3. Personal stigma 4. Psychological wellbeing 5. Perceived stress | 1. D-Lit  2. A-Lit  3. DSS - Personal Stigma subscale 4. SWPB (18-item) 5. PSS | Positive outcome |
| Tay, 2022 | 2022 | IMH | Young adults | Online | Male=50, Female=124 | Online HOPE intervention | Help-seeking | N=174 | 1. Recognition of depression 2. Help-seeking intentions | 1-2. Individual semi-structured phone interviews | Positive outcome |
| Teo et al., 2020 | 2020 | Duke-NUS | Adults, older adults | Hospital | Female=95 | Cognitive behavioural Therapy with Mindfulness and Values-based activity (CBT-MV) | Advanced breast cancer | N=95 | 1. Engagement 2. Psychological distress | 1. Participant ratings 2. HADS | Positive outcome |
| Teo et al., 2020 | 2020 | Duke-NUS | Adults, older adults | Hospital | Male=37 Female=23 | CBT-based intervention | Colorectal cancer | N=60 | 1. Psychological distress 2. Self-efficacy | 1. HADS 2. CBI (Version 2.0) | Positive outcome |
| Teo et al., 2021 | 2021 | CGH | Adults, older adults | Primary Care | Male=75 Female=152 | Health Wellness Programme | Mental disorders | N=228 | 1. Genetic health status 2. Depression 3. Functioning | 1. EQ VAS 2. PHQ-9 3. Sheehan Disabilty Scale | Positive outcome |
| Teo et al., 2024 | 2024 | NCSS | Adults | Hospital | Female=33 | Renewing Intimacy and SExuality Intervention (RISE) | Marital sexual distress | N=33 | 1. Marital satisfaction 2. Sexual satisfaction 3. Sexual dysfunction 4. Body image disturbance | 1. DAS-10 2. FSFI-Satisfaction subscale 3. ASEX 4. BIS | Positive outcome |
| Verma et al., 2021 | 2012 | IMH | Young adults, Adults | Hospital | Male=404 Female=391 | EPIP | Early psychosis | N=795 | 1. Psychopathology 2. Functioning | 1. PANSS 2. GAF | Positive outcome |
| Wang et al., 2015 | 2015 | SGH | Adults | Hospital | Male=380 Female=809 | Multi-component psychological intervention programme | Psychological wellbeing | N=1189 | 1. Anxiety 2. Depression 3. QOL | 1-2. HADS 3. IBS-QOL, EQ-5D | Positive outcome |
| Wang et al., 2018 | 2018 | NUS | Adults, older adults | Community | Male=115 Female=14 | Coronary Heart Disease Self‐management Programme (CHDSMP) | Self-management | N=129 | 1. Anxiety 2. Depression 3. HrQOL 4. Perceived stress 5. Self-efficacy | 1-2. HADS 3. SF-12 4. PSS 5. CSE Scale | No effect |
| Wong et al., 2018 | 2018 | SGH | Adults | Hospital | Male=2 Female=34 | Mindfulness-based training programme for nurses | Work performance | N=36 | 1. Sustained attention | 1. PVT, EEG Responses | Positive outcome |
| Wong et al., 2020 | 2020 | NUS | Adults | Community | Female=39 | Mental health paraprofessional training for FDWs using CBT principles | Peer support | N=39 | 1. Depression literacy 2. Knowledge of CBT 3. Confidence in supporting individuals with depression 4. Help-seeking attitudes 5. Attitudes towards depression | 1. D-Lit  2. Knowledge of CBT questionnaire  3. Single item scale 4. ATSPPH-SF 5. DSS | Positive outcome |
| Wong et al., 2021 | 2021 | KKH | Children | Hospital | Male=18, Female=7 | Music Therapy | Wellbeing | N=25 | Individualised goals achieved (physical and mental functioning) | 1. Goal Attainment Scale | Positive outcome |
| Woo et al., 2022 | 2022 | NUS | Adults, older adults | Hospital | Male=29 Females=14 | Integrated Chronic Care E-Enhanced Atrial Fibrillation (NICE-AF) | Atrial fibrillation | N=43 | 1. Depression | 1. PHQ-9 | Positive outcome |
| Xie et al., 2015 | 2015 | IMH | Children, adolescents | School | NR | Response, Early Intervention and Assessment in Community Mental Health (REACH) | Mental health | N=1500 | 1. Behavioural and emotional problems 2. Severity of illness 3. Functioning | 1. SDQ 2. CGI 3. CGAS | Positive outcome |
| Yang et al., 2017 | 2017 | TTSH | Adults | Hospital | Male=8 Female=25 | iACT-CEL (Acceptance and Commitment Therapy intervention) | Chronic pain | N=33 | 1. Global life satisfaction 2. Depression 3. Pain acceptance 4. General/psychological acceptance 5. Committed action | 1. SWLS 2. PHQ-9 3. CPAQ-8 4. AAQ-II 5. CAQ | Positive outcome |
| Yang et al., 2017 | 2017 | IMH | Young adults, adults, older adults | Hospital | Male=7 Female=30 | Mindfulness-based training program | Stress and burnout | N=37 | 1. Mindfulness 2. Self-compassion 3. Compassion for others 4. Perceived stress 5. Burnout | 1. FFMQ 2.SCS-SF 3. CS 4. PSS-10 5. OLBI | Positive outcome |
| Yap et al., 2014 | 2014 | KKH | Adults, older adults | Hospital | Male=374 Female=641 Caregivers (grandparents) = 6 (gender not available) | Signposts for Building Better Behaviour programme | Parenting skills | N=1021 | 1. Parental efficacy and satisfaction 2. Depression 3. Anxiety 5. Stress 6. Hassles 7. Child's difficult behaviour 8. Child aggression and compliance | 1. PSOC 2-5. DASS 6. PHS 7. DBC 8. DBAF | Positive outcome |
| Yap et al., 2017 | 2017 | Duke-NUS | Older adults | Community | Male=2 Female=29 | Rhythm Wellness Programme | Psychological wellbeing | N=54 | 1. QOL 2. Depressive mood 3. Sleep quality 4. Social isolation | 1. EQ-5D 2. GDS 3. PSQI 4. LSNS | No effect |
| Yap et al., 2019 | 2019 | KKH | Adults | Hospital | Female=285 | Signposts for Building Better Behaviour programme | Parenting skills | N=285 | 1. Parenting efficacy | 1. PSOC | Positive outcome |
| Yeo and Choi, 2011 | 2011 | NTU | Children | School | Male=82 Female=13 | Cognitive-behavioural therapy | Disruptive behaviour | N=95 | 1. Impulsivity and self-control 2. Classroom and home behaviour 3. Self-esteem | 1. Teacher self-control rating scale 2. Teacher rating scale, Student rating scales 3. Self-esteem scale | Positive outcome |
| Yeo et al., 2016 | 2016 | NTU | Children | School | Male=70 Female=45 | School-based intervention for test anxiety | Test anxiety | N=115 | 1. Test anxiety | 1. CTAS | Positive outcome |
| Yeo et al., 2021 | 2021 | GERI | Older adults | Community | Male=26 Female=68 | Computerized cognitive training (CCT) NeeuroFIT programme | Cognitive function | N=94 | 1. Cognition 2. Gait | 1. CTT-2, RBANS, BBS 2. GAITRite walkway measures | Positive outcome |
| Zhang et al., 2025 | 2025 | CGH | Young adults, adults, older adults | Community | Male=41 Female-22 | Cancer Prehabilitation Exercise Diary | Prehabilitation | N=63 | 1. Anxiety 2. Depression | 1-2. HADS | No effect |
| Zhao and Leong, 2014 | 2014 | IMH | Young adults, Adults | Community | NR | Community Mental Health Team (CMHT) | Mental disorders | N=3652 | 1. Length of inpatient stay 2. Number of admissions | NR | Positive outcome |
| Zheng et al., 2020 | 2020 | NTU | Older adults | Community | Male=63 Female=257 | Exergames (exercise games) | Wellbeing | N=319 | 1. Sociability 2. Positive and negative affect | 1. Sociability Scale 2. PANAS | Positive outcome |
| Zheng et al., 2022 | 2022 | NUHS | Adults | Hospital | Female=204 | Web-based and home-based postnatal psychoeducational interventions | Postnatal wellbeing | N=204 | 1. Maternal parental self-efficacy 2. Social support 3. Postnatal depression, 4. Anxiety | 1. PMPSES  2. PICSS  3. EPDS  4. HADS-A | Positive outcome |
| Zhou et al., 2017 | 2017 | MSF | Adults | Community | Male=37 Female=70 | Positive Parenting Programme (Triple P) | Parenting skills | N=107 | 1. Caregiver perception of child's behaviour problem 2. Parenting style 3. Depression 4. Anxiety 5. Stress 6. Parenting self efficacy 7. Parenting satisfaction 8. Attribution style for child's disruptive behaviours 9. Parental anger in response to child caring | 1. ECBI 2. PS 3-5. DASS 6-7. BAPS 8. PACBM 9. PAI | Positive outcome |
