# Supplementary material for: Mapping programmes for mental health promotion in Singapore: A scoping review
Source: PLoS One. 2026 Apr 28;21(4):e0347518. doi: 10.1371/journal.pone.0347518 (PMC13124008; doi:10.1371/journal.pone.0347518)
Supplement: S4 Table — (DOCX) [file pone.0347518.s004.docx]

**S4 Table: Study characteristics based on TIDieR checklist list for those targeting mental health outcomes in those with mental health and neurological conditions**

| **Author** | **Name of intervention** | **Rationale/goal of elements essential to the intervention** | **Materials used in the intervention** | **Procedures** | **Provider's details** | **Mode of delivery** | **Location** | **Timing and dose** | **Tailoring** | **Modifications** | **Fidelity** | **Actual adherence** |
| --- | --- | --- | --- | --- | --- | --- | --- | --- | --- | --- | --- | --- |
| Aloweni et al., 2022 | Mindfulness-based intervention (MBI) | To determine the effect of MBI on caregiver (CG) stress, anxiety, quality of life (QoL) and reactions to caregiving, and to evaluate the feasibility of MBI and feedback on the programme among CG of patients undergoing peritoneal dialysis (PD). | Reading and audio materials to guide practice, logbooks | The Treatment as Usual (TAU) group received structured PD training for 4.5 days while the Mindfulness-based Training (MBT) group received structured PD training along with mindfulness training for an hour in the evening at home and feedback on the next day. Breathing exercises were done in the morning and body scans in the evenings. MBT techniques were included and practised in the PD process daily. The Research Coordinator (RC) contacted the participants weekly for first 4 weeks and monthly up to 6 months. Outcome measures were recorded at baseline, 1-, 3- and 6-months post randomisation. | Trained clinical psychologist. RC conducted assessments. | Face to face sessions and individual practice at home | Outpatient clinics of acute care hospitals | 1-hour MBT session with daily practice | MBT was integrated to PD procedure which was tailored for home environment. Participants' feedback and follow-up calls were integrated to the programme. | The proposed sample size was not met due to eligibility issues, high dropout, and limited funding. | The MBT intervention and close follow-up were planned systematically and tailored for CG of patients with PD in home environment. Randomisation, practice and log books, trained team members and use of validated tools added to the fidelity. | 138 CG were required, but actual sample only had 44 CG. Dropout rates were indicated to be high. Intention to Treat (ITT) analysis was not conducted. A reduction in stress and anxiety was achieved. |
| Chan et al., 2013 | Life storybook | To examine the effects of life storybook compared to control on reducing depressive symptoms in community dwelling older adults. The framework was developed based on Theory of Psychosocial Development model. | .audio-recorders, interview guides and materials to develop storybook (photographs) | Participants with Geriatric Depression Scale (GDS) scores of 4 or more were randomised into either intervention or control group. For the intervention group, 5 interview sessions lasting 30-45 minutes each were conducted spanning 8 weeks. The first interview centred on childhood and adolescence memories, second and third on subject's memories relating to adolescence and adulthood, and fourth on summarising subject's current life and as a whole. Transcripts were synthesised into life story books which was reviewed by the participants on the fifth interview at week 8 which was held 1 month later. Participants were gifted the book for keeping. GDS scores were collected at all 5 time points for the intervention and control group. | Registered Nurse (RN) | Face to face individual session | Participants' home | 5 time points, week 1-4 and last session on week 8 | NR | NR | The study participants were of Chinese ethnicity with no rationale specified for ethnic restriction. While the study was a Randomised Controlled Trial (RCT), sample size was too small with no attrition reported. Validated measures and trained staff were included. Included qualitative interviews which improved clinician's understanding of the patients’ conditions. | Study conducted as planned. 26 participants completed with no dropouts and a reduction in depressive symptoms was achieved. |
| Chan et al., 2014 | Life story review | To assess effectiveness of life-story review on depression levels in Malay older adults based on a structured reminiscent approach using personal memories and experiences as reported in previous literature. The theory posits that reviewing and documenting life experiences can improve mental wellbeing. | Memorabilia, such as photos or personal items, life storybooks | Participants were randomised into intervention and control groups. Life storybooks containing text and photographs were narrated for each participant through 4 weeks of interviews that were .audio-recorded and included recollection of memories of childhood and family celebrations in the first interview, adulthood, marriage, and other important phases of their life in the second interview, reviewing the contents from past sessions in the third and fourth interview, The fifth interview was conducted on week 8, during which the book was gifted to the participant. GDS scores were collected at all 5 time points for the intervention and control group. | RN with more than 10 years’ experience in geriatric care and research team. | Face to face | Participant's home | 30–45-minute session per week for the initial 4 weeks and last session on week 5. | Individual life stories constructed using personal memorabilia in a language preferred by the participant. | NR | Structured interviews on a weekly basis at a location and language comfortable for the participants. The interviews were audiotaped for easy analysis. Measurements (GDS-15) taken after each interview. | All 29 participants completed the 5 sessions and showed improvement in depressive symptoms. Life story books and data collection completed for all as planned. |
| Chew et al., 2025 | Activities of Daily Living+ (ADL+) | To prevent cognitive decline in community-dwelling seniors without dementia but with subjective memory complaints, through an AI-enabled intervention targeting cognitive, physical, and nutritional domains. | AI-enabled platform with modules, Smart Day Activity app, iPads, Kinect systems | The programme included 7 modules embedded within the ADL+ toolkit. The Smart Day Activity app is the main interface for the modules, which offer gamified interventions targeting cognitive, physical, and nutritional aspects while serving as a cognitive monitoring tool. Participants receive a customised task schedule on the Smart Day Activity app each day and logged their diet 3 times daily. There are also weekly community-based activities found in some modules that participants can register for. | Self-directed | Mobile app | Community | Cognitive stimulation activities daily, diet logging 3 times a week, 2 community-based activities a week, all over 6 months | Data gathered from the toolkit, such as diet logs and game performance, were used to personalise interventions for the user according to their cognitive status. | As the study coincided with the Covid-19 pandemic, recruitment was adjusted to align with Covid-19 restrictions. | Participants were recruited on stringent criteria, requiring subjective memory complaints, and modified Chinese Mini Mental State Examination (mCMMSE) scores above age- and education-specific dementia cutoffs. Participants were excluded if they had known or suspected dementia or significant medical conditions. Validated scales were used to measure outcomes. Data were collected at 3 timepoints: baseline, post-intervention, and at 3-months follow-up. Adherence was tracked for all modules. Sample size was calculated to account for 20% dropout rate. | High adherence was noted with 73 of 75 participants completing the programme. Improvements in neuropsychological battery composite scores, attention, processing speed, memory, and quality of life were observed. |
| Huang et al., 2017 | General Practitioner Partnership Programme (GPPP) | To distribute the responsibility of caring for psychiatric patients with general practitioners (GP) so that the increasing demand for psychiatric services can be managed. | NR | The programme included tailored financial counselling, sharing on available financial schemes, and assuring patients of GP's capability and capacity to care for them. | Case managers (CM) | NR | Hospital | NR | Tailored to patients' needs | None | Data was collected pre- and post-intervention. A single-group interrupted time-series analysis was used to determine impact of intervention in accordance with rollout of the 3 interventions in the programme. Data on patients who returned to the hospital was tracked as a balancing indicator. | The programme increased referral acceptance rates from 64% to 92%. |
| Jiao et al., 2019 | Web-based and home-based postnatal psychoeducational intervention | To increase parental self-efficacy, social support, and psychological wellbeing in first-time mothers. The theoretical framework relies on Bandura's self-efficacy theory. | Website, booklet | The web-based intervention involved access to a tailored website delivering psychoeducation for one month. The home-based intervention involved home visits and a booklet. Both interventions were accompanied with routine care. | Research assistant (RA) | Online website and face to face home visit, individual | Participant's home | NR | None | None | The study was a single-blind procedure. Participants were randomised into the 3 groups. A power analysis was done, and participants were recruited to account for attrition. Data was collected for the 3 groups at 4 timepoints - baseline and repeated post-tests at 1 month, 3 months, and 6 months post-intervention. Validated scales were used to measure outcomes. ITT analysis was done. | Both web-based and home-based interventions improved social support. The web-based intervention group showed improved self-efficacy and reduced postnatal depression. |
| Kasmani et al., 2018 | Multilevel Bidirectional (MLB) Care Coordination Model | To increase referrals to community-based residential psychiatric rehabilitation facilities (CBPRFs) to ensure continuity of care and increase bed capacity in IMH. | Post-discharge phone calls | The programme involved a weekly referral exercise where identified cases by the Institute of Mental Health (IMH) Care Coordination Team (CCT) are reviewed by a CBPRF panel. The CCT then coordinates with patients and caregivers depending on the referral outcome. Bimonthly meetings are held between the CCT and CBPRF representatives to review the weekly referrals and examine ways to increase successful referrals. | Medical social workers (MSW) | Face to face group sessions | Community | 1. Weekly referral exercise 2. Bimonthly network meetings | Joint case management is initiated on a case-by-case basis for complex cases. | None | Annual referrals and referral outcome data are tracked. The CCT is made up of trained MSWs. Patients are pre-screened by MSWs before formal referrals are made. | The model showed initial success in increasing referral acceptance rates from IMH to CBPRFs. |
| Khine et al., 2020 | Mindful Awareness Programme (MAP) | To improve cognitive function in elderly with Mild Cognitive Impairment (MCI). The theoretical framework relies on gut-brain axis theory and mindfulness practices. | Mindfulness practice materials, body scan practice, visuomotor limb tasks. | The programme involved weekly, 40-minute mindfulness awareness practice sessions guided by trained instructors in the first 3 months. Participants were instructed to practise daily. Thereafter, sessions were delivered monthly for 45 minutes each. | Experienced instructors | Face to face group sessions | Hospital | 3 months weekly sessions and 6 months monthly sessions | None | None | Assessments using neuropsychological tests and gut microbiota analysis. | Moderate adherence was noted with 18 of 28 MCI participants completing the intervention. The MAP group showed significant improvement in cognitive function. |
| Klainin-Yobas et al., 2016 | S-Manage Stress Management Programme | To reduce stress and improve psychological health in mental health inpatients using psychoeducation and relaxation. The theoretical framework relies on relaxation theory. | Stress information materials, relaxation scripts, physiological tools | The programme involved daily 1-hour sessions delivered in a group format. Sessions began with a recap of previous content and group sharing of thoughts and experiences. This was followed by interactive psychoeducation on a preset topic and ended with relaxation techniques. | Researchers | Face to face group sessions | Hospital | 1 hour session per week over 4 weeks | None | None | Power analysis was done to ensure appropriate sample size. A manual was available to guide facilitators. Psychoeducation topics were preset for each session. Physiological measures and validated scales were used for outcome measures. Data were collected pre- and post-intervention. | All participants completed the intervention. The intervention improved objective and subjective stress and psychological health. |
| Koh et al., 2020 | Person-centred Creative Dance Intervention | To promote QoL, wellbeing, and physical function in persons with mild to moderate dementia. | Music, movement prompts, creative dance activities | The intervention involved weekly 1-hour creative dance sessions over 8 weeks, conducted by a creative movement therapist and embedded into the day care centres' regular activities. Each session included warm-up exercises, improvised movements, arm movement exercises, and opportunities for social interaction among participants and therapist. | Creative movement therapist | Face to face group sessions | Daycare Centre | 1 hour session per week over 8 weeks | Improvised movement based on music familiar to participants' generation | None | Participants were recruited on stringent criteria including age and medical diagnosis of mild to moderate dementia. Validated scales were used for outcome measures. Data were collected pre- and post-intervention. | 2 participants were excluded from the study due to inconsistent attendance. For the remaining 35 participants, high adherence was noted with 30 participants attending 7 or more sessions. Statistically significant improvements in QoL and wellbeing measures. |
| Lai et al., 2019 | Collaborative Memory Clinic Model | To improve early detection and management of dementia through integrated care between primary care and geriatric psychiatry. | Electronic medical records, care protocols, assessment tools | The programme included a first visit consultation, which includes assessments with the patient and caregiver conducted by a CM, followed by a joint consultation with a family physician (FP)and psycho-geriatrician. Thereafter, community partners conducted home visits, and the patient attended joint consultation reviews about 2-3 months after the first consultation. Stable patients would be discharged from the Memory Clinic for follow-up at family medicine physician clinics or care programmes. | CM, FP, geriatric psychiatrists, community partners | Face to face consultations | Outpatient clinic, home visits | NR | Care plans individualised for each patient. Composition of home visit team depends on patient needs. | None | Audit of service delivery processes. Validated scales used for first assessment with patient and caregiver. | High retention and management rates within primary care without escalation. |
| Lee et al., 2003 | Child Guidance Clinic Mental Health Services | To provide mental health assessment and treatment for adolescents in Singapore. | NR | The programme involved outpatient mental health assessments, psychotherapy, and pharmacotherapy. | Psychiatrists, psychologists, social workers, nurses | Face to face clinical management | Outpatient clinic | NR | None | None | Review of clinic records and service utilisation. | Increased adolescent service usage and referrals from polyclinics and schools; depression most common diagnosis. |
| Lee et al., 2016 | Postpartum Depression Early Intervention Programme | To improve symptoms, functioning, and health related QoL in postpartum women with risk of postnatal depression through a screening and early intervention programme. | Assessment tools | The programme involved screening for postnatal depression to identify women at risk of postnatal depression. Interventions were customised based on severity, such as counselling, psychotherapy, and pharmacotherapy. Participants were also encouraged to participate in support groups. | Mental health CM, psychiatrists, therapists, social workers, community partners | Face to face | Outpatient clinic | At least 2 visits for 89.7% of participants | Intervention was customised based on each patient's depression severity and needs. Support group participation was encouraged but not compulsory. | None | Participants were screened by trained perinatal mental health CM using a validated postnatal depression scale. Validated scales were used to measure outcomes. Data were collected at baseline and post-intervention. | No participant withdrawal was noted. Improvements in postnatal depression, functioning, and QoL scores were reported. |
| Lee et al., 2024 | Intellect | To reduce subclinical obsessive-compulsive disorder (OCD) symptoms among university students in Singapore. The theoretical framework relies on Cognitive Behavioural Therapy (CBT) with Exposure and Response Prevention (ERP). | Intellect mobile app, daily reminders via WhatsApp/Telegram | The programme involved an 8-day OCD intervention on the Intellect mobile app, which provided psychoeducation on OCD, re-exposure and imaginal exposure, family accommodative behaviour and how it hinders recovery, and ERP. On days 2, 5 and 8, there were activities for participants to complete. Participants spent 5 minutes on the app daily for 8 days. | Self-directed | Mobile app | Community | 5 minutes daily for 8 days | None | None | Participants were pre-screened for OCD symptoms severity and randomly assigned to the treatment and comparator arm, and blinded to the study objective. Participants were not allowed to use any other mHealth apps or mental health services during the intervention period to prevent potential confounding effects. Adherence was ensured by sending daily reminders via WhatsApp/Telegram and mandating participants to send their daily logs to the investigator. Validated scales were used to measure outcomes. Data were collected at 3 timepoints: baseline, post-intervention, and 4-weeks follow-up. Data quality checks (rushed responses, long string analysis, attention checks) and ITT analysis was done. | High adherence was noted with 224 of 225 participants completing the intervention. The intervention group showed significantly lower OCD symptom severity compared to the control group at both post-intervention and 4-week follow-up. |
| Loh et al., 2023 | Sure Mums intervention | To improve mother-baby bonding in a group of new mothers which could reduce postnatal depression. The programme was developed based on cognitive behavioural family therapy, which uses cognitive restructuring and behavioural modifications to improve mother-baby interactions. | NR | All the mothers received 1 to 5 home-based therapy sessions that included validating mothers' difficult experiences and emotions, reflecting the needs of baby through direct observation of baby’s behavioural cues, speaking about baby using language that invokes mother’s curiosity and understanding of baby’s mind, soothing mother’s and baby’s emotions during challenging situations and identifying positive interactions between mother and baby, encouraging more of these moments. | Clinical Counsellor | Face to face | Participants' residence | 1 to 5 sessions, 1 hour each, conducted with a year of birth. | The number of sessions were tailored to the needs of the mother | NR | Self-reported outcome measures such as Postpartum Bonding Questionnaire (PBQ), Global Assessment of Functioning (GAF), and Edinburgh postnatal Depression Scale (EPDS) were used which were well validated scales and the interventions were delivered by a trained clinical counsellor. The questionnaires used aligned with the aim of the study. | The study team recruited 32 mothers, and 7 mothers dropped out. The analysis includes only 25 mothers. The intervention improved bonding and depression. |
| Low et al., 2013 | Assertive Community Management ACM) | To prevent relapses and reduce hospitalisation rates in patients with severe mental illness. This includes treatment monitoring, rehabilitation and support services primarily through visits to the patients at their homes or workplace. | NR | Participants who were not helped by the traditional outpatient services were referred by the psychiatrist based on severity and persistence of mental illness. Those who had 3 admissions or more and/or duration of hospitalisation for more than 30 days in the past year, with severe disability and poor functioning were included. A multidisciplinary team (MDT) visited patients at their home or workplace to deliver the care. Outcomes (number of admissions and total length of stay in the past year pre and post ACM period) were drawn from the medical records. | MDT | Face to face | Participants' residence | Patients were only discharged after meeting the discharge criteria such as improved functioning in all areas, self-initiate discharge, admission to rehabilitation or chronic stay wards. Outcomes monitored for 1 year. | Tailored to local setting and the patient population (aged 18-65 years, severe mental illness, or disability, having 3 or more admissions or 30 or more days hospitalisation) | NR | Care was delivered by trained MDT and the outcome measures were captured from the medical records rather than self-report. | It is not clear how many patients were offered ACM, among whom 156 patients accepted and 155 completed the programme. The programme showed good outcomes (reduced hospitalisation and length of stay). |
| Ng et al., 2020 | Community-Based Early Psychiatric Interventional Strategy (CEPIS) | To determine the effectiveness of collaborative care versus standard care for managing depression in older adults in primary care setting. Collaborative care involves a CM who works together with primary care and mental health professionals to improve the treatment outcomes of depression which is often under diagnosed and under treated. | Practice guidelines on the primary care treatment of depression for GPs, structured summary report of anti-depressant treatment and specialist referral along with various psychometric scales. | Participants were randomised into collaborative care (CC) and usual care (UC) groups, both of whom received care for 6 months with follow-up interviews at 3,6 and 12 months. For CC group, nurse educators visited the participants at home to deliver sessions on anti-depressants therapy and basic mental hygiene and counselling support, each session lasting 1 hour per week for 6 months. For usual care, patients received anti-depressant treatment referral to specialist if needed. Assessments were done during the follow-up interviews. The CEPIS programme encompassed 4 key components, beginning with neighbourhood outreach conducted through social service and activity centre portals, followed by routine screening of depressive symptoms. The programme then provided individual psychoeducation delivered by community nurses to encourage treatment acceptance. Finally, primary care treatment was administered by neighbourhood GPs. | GPs trained by a hospital psychiatrist on assessment, diagnosis, and management of depression. CM provided care support for referrals, treatment adherence and appointment booking. A trained nurse educator delivered the psychoeducation. Psychotherapist or psychiatric specialist where specialist service was needed. | Face to face | GP clinics and participants' residence | 1 hour session per week for 6 months | Personalized treatment was provided as per the care management protocol. Referral to specialists as needed. | NR | Well executed randomised (permuted blocks) blinded trial with validated measures to capture the data. Care management protocol for personalized treatment was executed. ITT analysis was planned. | 22 and 11 participants did not complete the follow-up for CEPIS and UC respectively. The protocol was carried out to all participants regardless and ITT analysis was conducted. Treatment receipt and patient satisfaction were captured. The intervention showed better treatment outcomes. |
| Nyunt et al., 2009 | Community-Based Early Psychiatric Interventional Strategy (CEPIS) | To investigate the impact of the programme to address the treatment seeking barriers in older adults with depression and provide community-based care for older adults. CEPIS involves a CM who works together with primary care and mental health professionals to improve the treatment outcomes of depression which is often under diagnosed and under treated. Core components include community outreach, screening, psychoeducation, and primary care treatment. | Care protocol and treatment algorithm for GPs along with validated questionnaire for screening and data collection. Printed material for patient education. | The programme included community wide outreach through 42 social service sites where free meals, social, recreational and community services to address various daily and treated needs of older adults, active screening and case detection, psychoeducation by trained nurses, support by CM and primary care engagement in an integrated structured collaborative shared care framework. Trained nurses and counsellors gave home-based education to the participants. | Trained CM, nurse educator, psychotherapist or psychiatric specialist and GP | Face to face session at the participant's residence and primary care consultations as group and individuals | GP clinics and participants' residence | After the initial screening, follow-up was scheduled after 2 weeks | The CEPIs programme was tailored to be culturally appropriate, in a language suitable for the local population, convenience of participants was taken into account for location. Conducted in individual or group sessions with personalised case management support. | NR | Study materials were translated in all local languages and the translations were validated. The questionnaires used were validated. Trained staff conducted the study procedures. | Study was conducted as planned. CM tracked the referrals and completion. No data on treatment dropouts. The programme improved help seeking. |
| Ong et al., 2019 | Regna Tales (mobile app) | To evaluate the usability and playability of a mobile app called "Regna tales" in reducing aggression. The theoretical basis of the app is CBT and problem-solving skills training for anger management. | Mobile apps, iPad, and validated questionnaires | Participants were asked to play the assigned regna app for 50 minutes before answering the questionnaires. Pre and post assessments were taken. Apps 1-4 teaches anger management skills and 5-6 focus on daily life skills. | NR | Face to face | Clinical setting | 50-minute session | Evolved from original face to face therapy to a web based and then to the interactive role-playing game app called Regna tales. Apps were developed to target different skills. | Apps 5 and 6 which were non game based were modified to add additional domain to assess knowledge attitude and intentions related to anger management. | The app was developed on a Corona development platform for mobile apps with the back end running PHP and a MySQL database on a Microsoft Windows Server. After the development 12 children were asked to test the different apps. Validated measures were used to capture the outcomes. | 72 children used the app. Although most of the procedures was done as planned, for 2 participants who used Abaddon mobile app, data was lost due to a server error. Improvements in anger management skills and emotion regulation was noted. |
| Pat-Horenczyk et al., 2015 | Building Emotion and Affect Regulation (BEAR) programme | To investigate the feasibility of the BEAR programme and its impact on regulation abilities and general coping of the children. It included mindfulness and CBT elements and was initially developed by the Israeli centre for the treatment of psychological trauma and was intended to build resilience among traumatised children aged 7-12 years. | BEAR manual, cardboard boxes for iBOX session, picture cards, cards showing different emotions, candles, and soda cans | The programme was an intervention with 5 core components: mindfulness, psychoeducation, experimental exercise, narrative approach and opening and closing rituals conducted in 6 sessions. These 6 sessions focused on social support, interpersonal regulation, cognitive emotional regulation, physical regulation and awareness of others. | 42 trained staff with majority psychologists and care staff were involved. 2 facilitators managed each group with a local supervisor per group. All received a 5-day training (on BEAR programme protocol) and were experienced in working with children in residential homes. Continued training was given through scope and email. | Face to face group sessions | Residential Homes | 6 sessions, 1.5 hours each, conducted biweekly or weekly | The programme was tailored for children's residential group homes locally. Cultural adaptations for the mindfulness element. | Apart from the cultural amendment to rename the mindfulness element, an exception was made to include a 13-year-old child. | Planned supervision and monitoring including a local supervisor and continued training and supervision by senior team. Pre and post assessments were taken. Information was captured from children, CG, and facilitators. | 3 dropped out from the 73 children and 10 groups completed. Procedures and measurements were delivered as planned with adaptations made to suit the local culture and setting. The programme improved coping and stress. |
| Rawtaer et al., 2015 | Community-based psychosocial interventions: Tai-Chi Exercise (TCE), Mindfulness Awareness Practice (MAP), Music Reminiscence Therapy (MRT) and ART Therapy (AT) | To study the impact of the intervention in improving the mental health outcomes of community dwelling older adults with subsyndromal depression and anxiety. | Evergreen songs, photographs, and pictures for MRT, art piece for AT | Eligible participants underwent screening and were assigned to either one of the 4 programmes of their choice. They received weekly activities for 10 weeks, fortnightly for 18 weeks and monthly for the rest of the year. Assessments were taken at week 1, 4, 10 and 24 and 1 year. The first 20 minutes of sessions for 6 months included health education. Initial 10 weeks focused on single intervention and involved designated activities for 30 minutes. The rest of the intervention period included combination of interventions which were 30 minutes each, lasting 2 hours per session. | Training conducted by qualified instructors, including certified psychotherapist and Tai Chi trainers with more than 10 years’ experience. Community nurses assisted in the assessments. | Face to face group sessions | Community-based research centres | Weekly sessions for 10 weeks, fortnightly sessions for 18 weeks, and monthly for the rest of the year. Session length was 50 minutes in the initial phase and 2 hours for combined intervention. | Participants were allowed to choose the intervention in the initial phase. | Individuals’ interventions were modified to combined interventions as per participants' request. For AT, participants indicated preference for narrative segments. | The sessions and interventions were planned in a structured way at regular sessions following a 1-year span. Validated tools were used at regular intervals. All interventions were provided by trained instructors with more than 10 years’ experience. | An initial drop in 10% participation led to modification of the protocol. Among 101 people who were assigned 99 completed the 1-year follow-up. A reduction in subsyndromal depression and anxiety was achieved. |
| Saxena et al., 2018 | Primary Care Dementia Clinic (PCDC) | To build capability of a MDT for community management of dementia patients, develop integrative structure and processes for PCDC and tertiary hospital Memory Clinic, and allocate resources appropriately and increase the overall capacity of dementia care, while providing seamless and prompt access to primary and tertiary dementia care. | NR | The programme involved a 15-minute consultation with a FP and a 15-minute consultation with a nurse in the polyclinic, co-ran with tertiary hospital clinicians. Referrals to Allied Health services were made where necessary. Every 2 weeks, the tertiary hospital team held case conferences to help the polyclinic team address any concerns in the management of dementia patients. | FP, nurse, nurse clinicians, geriatricians | Face to face individual sessions | Primary Care (polyclinic) | 15-minute consultations with FP and nurse, 2 visits. Case conferences every 2 weeks | Tailored for the specific population | None | Participants were recruited on stringent inclusion and exclusion criteria. Sample size was calculated to account for attrition. Validated scales were used to measure outcomes at baseline, 12 months, and against comparator conditions. ITT analysis was done. | The PDPC group had better caregiver satisfaction than the other polyclinics group. The PDPC group had higher Quality Adjusted Life Years (QALYs) than the MC group. High adherence was noted, with 91.3% retention at 6 months and 85.9% retention at 12 months. |
| Shah et al., 2015 | Virtual Reality-Based Stress Management Programme | To reduce stress, anxiety, and depression in people with mood disorders using VR technology. The theoretical framework relies on the Neuman System Model. | VR device, relaxation videos, participant education manual, relaxation audio CD | The programme involved daily 1-hour sessions incorporating VR relaxation practice and psychoeducation over 3 days. Psychoeducation was delivered face to face in a ward setting, whereas relaxation techniques were taught using the VR device and relaxation videos after demonstration by facilitator. At the end of the session, the facilitator summarised the content and skills learnt and allocated time for discussion. | Researcher | Face to face discussion and use of VR | Hospital | 1-hour sessions daily, 3 consecutive days | None | None | Participant education manual was provided. Physiological measurements and validated scales were used for outcome measures. Data were collected pre- and post-intervention at fixed time points. ITT analysis was done to account for missing data from early discharge. Qualitative feedback was sought regarding the strengths and weaknesses of the programme. | High response rate of 84.62%. 3 of 22 patients had early discharge before completing the intervention. The intervention improved subjective and objective stress, depression, anxiety, perceived relaxation, and knowledge on stress and stress management. |
| Shorey et al., 2013 | Postnatal Psychoeducation Programme (PPP) | To enhance maternal self-efficacy, social support, and reduce postnatal depression in first-time mothers. The theoretical framework relies on Bandura's self-efficacy theory as well as knowledge on social support, self-management of newborns and postnatal depression. | Educational booklet, intervention protocol weekly journal | The programme included an initial 90-minute home visit conducted by a midwife, during which mothers were educated on the significance of maternal parental self-efficacy and social support, and the risks and symptoms of postnatal depression. This was followed by 3 weekly phone calls and the provision of an educational booklet to reinforce the information and provide ongoing support. | Trained midwives and nurses | Face-to-face (pre-discharge) and telephone (post-discharge). | Hospital | 90-minute home visit, 3 weekly telephone calls post-discharge | None | None | Validated scales used for outcome measures. Power analysis was done to determine adequate sample size. The intervention protocol and contents of educational booklet were validated by an expert panel. Data were collected at 3 timepoints: baseline, 6 weeks, and 12 weeks post-partum. | High adherence was noted with 108 of 122 participants adhering to protocol. The intervention improved maternal self-efficacy and social support, reduced postnatal depression. |
| Shorey et al., 2019 | Technology-Based Peer-Support Intervention Program (PIP) | To prevent postnatal depression in mothers by providing peer support through technology. | Training manual for peer supporters, mobile phones | The programme involved providing peer support over phone calls, emails, and other mobile communication platforms, delivered by peer volunteers trained by a psychiatrist. Sessions were tailored to the needs of the participants and were held at least once a week for 4 weeks. | Trained peer volunteers (experienced mothers) who received formal training by psychiatrist | Phone calls, emails, and other text messaging via mobile communication platforms. | Community | 4 weeks and weekly follow-ups via call or text | Sessions were tailored according to patient needs. | None | A research protocol was published. Peer volunteers attended a half-day training by a psychiatrist. Validated scales were used for outcome measures. Data was collected pre- and post-intervention at 1 month and 3 months postpartum. ITT analysis was done. | The intervention improved postnatal depression scores at 3 months postpartum. No withdrawals were reported. |
| Shorey et al., 2021 | Integrated Personal Therapy (IPT) | To provide psychosocial support for older adults with subsyndromal depression/anxiety through community-led, volunteer-driven interventions. The theoretical framework relies on solution-focused brief therapy and structured life review therapy. | Hardcopy training handouts | The programme included group-based solution-focused brief therapy and mindfulness training conducted by trained volunteers. | Trained community volunteers | Face to face group sessions | Community | NR | None | Due to COVID-19 lockdown and study cessation, 7 older adults in second batch of participants could not proceed with the study. Only 21 participants were included in the analyses. | Mixed methods approach was undertaken. Validated scales and physiological measurements were used for outcome measures. .audio recordings were used for transcripts and thematic analysis was done for qualitative feedback. Control group was allocated to waitlist. Mindfulness training was developed by experts. Data were collected pre-intervention and post-intervention at 3 months and 6 months. | High adherence was noted with 16 of 21 participants (76.2%) completing the study. The intervention had no significant effect on depression, anxiety, life satisfaction, friendship, and quality of life. |
| Sim et al., 2007 | Early Psychosis Intervention Programme (EPIP) | To improve clinical and functional outcomes in first episode schizoaffective disorder or schizophrenia through an inpatient early intervention programme. | NR | The programme involved treatment and follow-up for patients with first-episode schizoaffective disorder or schizophrenia over 24 months. | Clinicians | Face to face sessions | Hospital | 24 months | Tailored to the specific population | None | Participants were recruited on stringent criteria, requiring a formal diagnosis of first-episode schizoaffective disorder or schizophrenia, be within a specific age range (18 to 40) and without prior psychiatric treatment. Validated scales were used to measure mental health outcomes. Data were collected at five timepoints: baseline, 6 months, 12 months, 18 months, and 24 months follow-up. | No participant withdrawal was reported. Improvements were observed for positive and negative symptoms, general psychopathology, awareness of psychiatric illness, psychological health and functioning for both schizoaffective disorder and schizophrenia. |
| Sim et al., 2021 | Cyber Wellness Enrichment Programme | To treat gaming disorders in adolescents through a programme involving individual and family counselling, group mentoring, social support, and provision of alternative activities. | Screening tools, genograms, video games | Participants are assessed on gaming disorder symptom severity and core personal and interpersonal issues at the start of the individual counselling sessions. These sessions aim to help adolescents control their gaming habits through proper time management, restricting visits to specific games and more. Family counselling sessions are held to provide psychoeducation and counselling to parents to help them support their adolescents. Adolescents also attend healthy group gaming sessions and identify alternative activities to participate in. | Trained psychologists and counsellors | Face to face sessions | Social Service Agency | 3 months | Tailored to the specific population | None | Data were analysed by social workers with postgraduate qualifications in family therapy and experience working with adolescents and families. All interview content was kept confidential from the counsellors to reduce social desirability bias. | No participant withdrawal was noted. Improvements in gaming habits and parent-child relationships were reported. |
| Tan et al., 2015 | Psychiatric Housing Unit programme | To improve social skills, coping strategies, and life management skills in offenders with psychiatric disorders. | Craft materials, games | The programme involved an assessment phase, basic phase, and maintenance phase. The assessment phase included 2 weekly sessions over 3 months and focused on building rapport, increasing confidence, and enhancing social interaction. The basic phase included 3 weekly sessions over 3 months focused on maintaining social ties and reducing unhealthy coping strategies. The maintenance phase involved 3 weekly sessions over 6 months focused on life management skills, budgeting, building roles and routines, and improving social skills. | Occupational therapists (OT), part-time psychiatrist, nurses, prison staff | Face to face group sessions | Prison | 1 hour and 15 minutes sessions, 2-3 times weekly over 12 months, | Activities adjusted based on risk levels of offenders [three-tier General Workers Programme (GWP)] | Due to the effectiveness of occupational therapy, an additional therapist was recruited after a year. | A scale was developed to measure outcomes at all 3 phases. Scores on the scale were jointly decided by both occupational therapists. | The intervention was effective in improving all Task Behavioural Scale (TBS) domains by the end of the basic phase, which was maintained till the participants were discharged into mainstream prison or the community. High adherence was noted with 100% of participants completing the intervention. |
| Tan et al., 2017 | Illness Management and Recovery (IMR) Programme | To reduce symptoms, lower readmission rates, and improve social functioning in individuals with mental disorders through a recovery-focused intervention. The intervention was adapted from the IMR programme, which is evidence-based and practised in many states and countries. | Handouts | The programme involved biweekly home visits by community psychiatric nurses, covering 8 modules adapted from the original IMR programme. The 8 modules were recovery strategies, practical facts about mental illness, stress-vulnerability model and strategies for treatment, using medication effectively, reducing relapses, coping with stress, and coping with problems and symptoms. Each session focused on rapport building, reviewing past content and home assignments, following up on goals, setting the agenda for the current session, discussion on new materials and practising new strategies, deciding on the week's home assignment, and summary of progress in the session. | Community psychiatric nurses (CPN) with advanced diploma in mental health. One of the CPNs is also a Motivational Interviewing Network Trainer, Milestone Recovery Mentor and Psychiatric Rehabilitation Practitioner. | Home visits | Participant's home | 1-hour sessions, biweekly over 12 months | Tailored to the specific population | None | Participants were recruited on stringent inclusion and exclusion criteria. Validated scales were used to measure outcomes. Data were collected at 4 time points: baseline, mid-intervention, post-intervention, and 12 months follow-up. | High adherence was noted with all participants completing the intervention. Significant improvements in length of stay, admissions, psychotic symptoms, functioning, and recovery outcomes were observed. |
| Tan et al., 2021 | V-DESSERTS | To improve stress management in inpatients with mental disorders through a VR-based intervention. | iTVGoggles, education booklet, relaxation videos | The programme involved 2 sessions of face-to-face psychoeducation followed by VR-based relaxation techniques. The psychoeducation component covered the definition, effects, and management of stress. The VR component required participants to watch videos covering abdominal breathing and muscle relaxation. | Researchers | Face to face, VR technology | Hospital | 40 minutes per session, 2 sessions | None | None | Participants were recruited on stringent inclusion and exclusion criteria. Power analysis was done to determine sample size. Researchers were blinded to the intervention and control group allocation to prevent selection bias. A checklist was developed to standardise intervention delivery. The environment was standardised with participants using the same room with the same temperature. Validated scales were used to measure outcomes. Relaxation and subjective stress were measured before and after each session, while perceived stress was measured before and after the intervention. Qualitative feedback was also sought from the participants. | High adherence was noted with 95% completing the intervention. Significant improvements in subjective stress and perceived relaxation were observed. |
| Tan et al., 2022 | Arts and Dementia Programme | To improve wellbeing in people with dementia through community-based art activities. | NR | The programme involved four community-based art activities involving elements of art-appreciation, artmaking, singing, or dance and movement. Only one activity was offered at each day care centre, with 6 to 9 hourly sessions held per week. | Artists, organisation staff, volunteers | Face to face | Community | 1-hour sessions per week, 6-9 sessions | Tailored to the specific population | Participants attended only one of 4 activities, depending on what was offered at their day care centre. | A validated scale was used to measure outcomes. Data were collected at 4 time points: baseline, second session, mid-intervention, and post-intervention. | High adherence of 86.2% was noted. Improvement in wellbeing was observed. |
| Teo et al., 2021 | Health Wellness Programme | To better integrate mental health care with primary care by providing access to community psychotherapy services for patients with mild to moderate mental health conditions. | NR | The programme used a shared care model between team members (psychiatrists and therapists) and primary care physicians. Patients were referred to the programme by primary care physicians receive psychotherapy sessions by the therapists. Primary care physicians also receive psychiatric liaison support from the team for these patients. The therapists discuss the cases with psychiatrists at weekly case conferences and provide regular feedback to the primary care physicians. | Psychiatrists, clinical psychologists, counsellors | Face to face | Community | Flexible | Psychotherapy approaches were customised for each patient and number of treatment sessions was determined by therapists' clinical judgement. | None | Participants were recruited on stringent inclusion and exclusion criteria. Upon referral, therapists did intake assessments to determine eligibility. Validated scales were used to measure outcomes. Data were collected at baseline, every 5 sessions, and at the end of therapy. | No participant withdrawal was reported. Significant improvements in mental health patient-reported outcomes were observed. |
| Verma et al., 2021 | Early Psychosis Intervention Programme (EPIP) | To raise awareness of and reduce stigma towards psychosis, collaborate with primary care providers in patient care, improve clinical outcomes and alleviate caregiver burden for patients with first-episode psychotic disorder. | Medicine | The programme involved outreach and clinical treatment involving pharmacotherapy, counselling, and psychoeducation for patients with first-episode psychotic disorder. | Psychiatrists, psychologists, CM, social workers, nurses, OT | Face to face | Hospital | 2 years | Tailored for the specific population | None | Participants were recruited on stringent inclusion and exclusion criteria. Validated scales were used to measure outcomes at baseline, 3, 6, 12 and 24 months. Ratings for clinical scales were done by psychiatrists trained in the instruments. Interrater reliability checks were done periodically and assessed to be high. | The programme was effective in achieving symptomatic remission and improving functioning. Moderate adherence was noted with 54.7% completing 2 years of the programme. |
| Xie et al., 2015 | Response, Early Intervention and Assessment in Community Mental Health (REACH) | To improve mental health of children and adolescents through early assessment and intervention, build capacity in schools and community partners to detect and manage mental health problems, and develop a mental health support network for children and adolescents in the community. The REACH model is based on five operating criteria of quality care and a support mechanism involving regional health systems. | Phone calls | The programme involved a mobile REACH team that provides telephone consultation to schools and community partners. Through the telephone consultation, the REACH team triages referred cases and communicates the result to the school. If a referral was accepted, the REACH team would assess the child and develop treatment plans accordingly. Treatment plans could include further interventions by REACH team, or referrals to GPs, specialist clinics, or family service centres. REACH also conducts regular workshops and monthly case conferences to train community partners and schools, and identifies partners for collaboration in the provision of mental health services. | Doctors, psychologists, MSW, CPN, OT | Face to face individual assessments telephone consultations | School | NR | Treatment options are developed based on nature and severity of child's mental health issues. Trainings customised to the needs of community partners. | None | Validated scales were used to measure outcomes at pre- and post-interventions. | The programme was effective in reducing behavioural and emotional problems, illness severity, and improving functioning. |
| Yeo and Choi, 2011 | Cognitive Behavioural Therapy programme | To improve self-management of challenging behaviours in school children, through a school-based psychoeducational group involving CBT components. | Workbook, report card, stars, token prizes | The programme involved 10 hourly sessions of group-based CBT, twice weekly over 3 months, followed by 2 booster sessions 1-month post-intervention. The sessions were conducted in small groups of 6-8 students by a school psychologist. The sessions aimed to increase self-awareness, self-esteem, and self-control through psychoeducation on affective, cognitive, and behavioural strategies. Each student received a workbook on the strategies taught and a report card for monitoring of progress and effort. Students were incentivised to self-monitor and exhibit good behaviour through stars and token prizes. | School psychologist | Face-to-face group sessions | School | Total 10 1-hour sessions over 3 months, 2 times a week, 2 booster sessions 1-month post-intervention | None | None | A control group was included to measure unique effects of CBT. Validated scales were used to measure outcomes. Data were collected at baseline and post-intervention. | No participant withdrawal was reported for the main 10 sessions. CBT group improved significantly in school and home behaviours, self-control, social skills, and self-esteem compared to control group. |
| Zhao and Leong, 2014 | Community Mental Health Team (CMHT) | To reduce hospital admissions and length of stay of patients with mental health conditions, keeping them in the community for as long as possible. The theoretical framework references the IIMR model and Motivational Interviewing (MI) | Depot injections | The CMHT offers services including ACM for severe, persistent cases, involving treatment and psychosocial rehabilitation. Standard Care Management is provided for stable patients, involving behaviour-focused psychoeducation and motivational interviewing, and monitoring of medication compliance. A Mobile Crisis Helpline is provided for crisis situations, and a Community Psychiatric Nursing Service focuses on medication adherence and providing caregiver support. | Psychiatrists, medical officers, CPN, OT, MSW psychologists, counsellors | Face to face individual setting | Community | NR | Services that the patient receives depend on type and severity of needs | The IMR model was introduced to Standard Care Management. | Participants were recruited on stringent inclusion and exclusion criteria. Weekly team meetings are held to review cases and discuss management plans. | The programme was effective in reducing the length of inpatient stay and number of hospital admissions. No patient withdrawal was reported. |
