# Supplementary material for: Mapping programmes for mental health promotion in Singapore: A scoping review
Source: PLoS One. 2026 Apr 28;21(4):e0347518. doi: 10.1371/journal.pone.0347518 (PMC13124008; doi:10.1371/journal.pone.0347518)
Supplement: S5 Table — (DOCX) [file pone.0347518.s005.docx]

**S5 Table:** **Study characteristics based on TIDieR checklist list for those targeting mental health outcomes in those with physical health conditions**

| **Author** | **Name of intervention** | **Rationale/goal of elements essential to the intervention** | **Materials used in the intervention** | **Procedures** | **Provider's details** | **Mode of delivery** | **Location** | **Timing and dose** | **Tailoring** | **Modifications** | **Fidelity** | **Actual adherence** |
| --- | --- | --- | --- | --- | --- | --- | --- | --- | --- | --- | --- | --- |
| Chandran et al., 2024 | Multidimensional rehabilitation programme | To reduce the side effects of breast cancer treatment through education and therapeutic exercises. | Exercise equipment (e.g., dumbbells, machine weights, treadmill, resistance bands) | The programme included 24 sessions of hospital-based exercise classes, 12 sessions of home exercise, and 10 sessions of education classes over 12 weeks. For exercise, participants attended 2 hospital-based supervised group exercise classes a week, each lasting an hour. Participants also received home exercise prescriptions for a single 30-minute session per week. At the end of 12 weeks, participants attended a 2-hour survivorship transitional class on cancer monitoring and follow-up. | Physiotherapist, assistant, Occupational Therapist (OT), Advanced Practice Nurse (APN), Dietician, Medical Social Worker (MSW) | Face to face group sessions and home | Hospital | Exercise class: 1 hour session, twice a week over 12 weeks Education class: Once a week over 12 weeks | None | Participants who were unable to attend an exercise class were advised to do home exercises. | Participants were recruited on stringent criteria. To be included, participants had to be ambulatory women receiving breast cancer treatment, not pregnant, without uncontrolled medical conditions, and not already engaging in regular moderate to high intensity exercise. Assessors were blinded. Data were collected at three timepoints: baseline, post-intervention, and 6-months follow-up. Validated scales were used to measure outcomes. | High adherence was noted with 32 of 38 participants completing the programme, of which 25 attended at least half of the sessions. A decline in perceived cognitive ability was observed. |
| Griva et al., 2019 | Combined Diabetes and Renal Control Trial (C-DIRECT) | To support patients with comorbid late-stage renal diseases and diabetes mellitus (DM) in the management of their conditions. The theoretical framework relies on Social Cognition Theory and Motivational Interviewing. | Agenda mapping chart | The programme included 3 weekly bedside sessions delivered by diabetes specialist nurses to encourage self-management through motivational interviewing. The sessions were typically delivered in the first 30 to 60 minutes upon cannulation and connection to a haemodialysis machine. Patients choose the topic for discussion using an agenda mapping chart and collaboratively set goals for self-management. The Elicit-Provide-Elicit framework was used to facilitate awareness, provide personalised advice, and share clinical results. | Diabetes specialist nurses | Face to face individual bedside sessions | Community | 30-minute session per week over 3 weeks | None | None | Validated scales were used for outcome measures. Data was collected pre-and post-intervention. Qualitative feedback was sought from both nurses and patients on the experience of the intervention. ITT and per protocol analyses were also done to control for ethnicity. | High adherence was noted with 42 of 44 participants completing the programme. No significant improvement in mental health outcomes was observed. |
| Guna et al., 2022 | Biography and Life Storybook (BLSB) Intervention | To investigate if BLSB improves depression, satisfaction with care and quality of life (QoL) among older adults when compared to usual care. The intervention was developed based on literature review and Singapore's Agency for Integrated Care Dementia Resource Kit. | Life storybook materials (photos, artifacts etc.) | Nursing home residents were divided into intervention and control group. The intervention group received BLSB protocol by a nurse and the control group received recreational activities and healthcare training which included physical exercise. The intervention consisted of nurse-led sessions for 45-90 minutes. Initial 4 sessions included autobiography, photo reminiscence and artefact collection from a caregiver (CG). The participants actively participated in the production of the life story book by providing photos, feedback and designing the book. The life story was compiled to an individual life storybook at the end of these sessions. The next 4 sessions involved sharing between family, friends and fellow residents along with usual activities. Data collected at week 1, 2, 4, 8 and 12. | Trained nurse | Face to face group sessions | Nursing Home | 8 sessions over 3 months. 4 weekly sessions in the first month and 4 biweekly sessions thereafter for 2 months, each lasting for 45-90 minutes. | NR | NR | Participants were involved actively in story book creation including design choices of the book. Validated measures were used. Although regular assessments were conducted incorporating feedback, the study was quasi experiment involving a single site and small sample size. The intervention was provided by a single person. | All participants completed the assessments as planned and life story book was delivered which improved the outcomes. The intervention improved depression, QoL and life satisfaction. |
| He et al. 2015 | Therapeutic play intervention | To reduce children and parents' anxiety levels over the children's upcoming surgical procedure by familiarising them with the hospitalisation process and surgical procedure. | Videos, dolls, photographs | The programme included a 1-hour therapeutic play session, 3-7 days before the child's surgery. The session involved the use of videos, dolls, and photographs to familiarise the child and parents with the hospitalisation process and surgical procedure. | Researcher | Face to face dyad session with intervention delivered via video | Hospital | 1 hour intervention, 3-7 days before surgery | None | None | Validated scales were used for outcome measures. Data was collected pre-and post-intervention for both the intervention and control groups. Qualitative feedback was sought from parents. | All participants completed the study. No significant effect of the therapeutic play intervention in reducing anxiety levels was observed. |
| Hoong et al., 2023 | Chronic Disease Self-Management Programme (CDSMP) | To improve disease management and promote self-efficacy in patients living with chronic conditions. The theoretical framework relies on Bandura's self-efficacy theory. | NR | The programme included 6 weekly workshops delivered by trained allied health professionals (AHP). Participants were split into small groups, where they developed action planning and problem-solving skills to elicit behavioural changes, and shared experiences for support and co-solving of problems. | AHP | Face to face group sessions | Community | Weekly workshop sessions over 6 weeks | None | None | Participants were recruited from multiple sources. Validated scales were used for outcome measures. Data was collected pre-and post-intervention. | 258 of 461 participants returned for the post-intervention questionnaire. Adherence was not reported. There was a significant improvement in self-efficacy, social activities, sleep, and depression. |
| Leow et al. 2015 | Caring for the Caregiver Programme | To help caregivers (CG) of advanced cancer patients to cope with CG stress. The theoretical framework: self-efficacy (Personal mastery, vicarious experiences, verbal persuasion, physiologic feedback) | Video clips, follow-up phone calls and support groups. | The programme included face to face sessions of psychoeducational sessions to cope with stress (video, discussions, follow-up calls, online forum), improve communication between the patient and caregiver (video, discussion of care plan, follow-up calls), increasing social support of the CG (video, discussion of care plans, follow-up calls, online forum), provide CG with information on advanced care planning and community resources (video, discussion of care plan and follow-up calls) | Nurse Researcher | Face to face and online/tele sessions | Hospice | Discussion of care plan : 40 minutes follow-up calls: 15-13 minutes Online forum: 15-30 minutes | None | None | Participants were recruited from the hospice based on stringent criteria which included the prognosis of the patient, duration of admission and caregiving commitment to standardize the severity of caregiving burden across the board. Outcome measures were relevant and included stress, depression, quality of life (QoL), positive aspects of caregiving, self-efficacy, relationship between CG and patient, and social support. | Intervention was delivered a as planned. All 38 CG in the intervention group completed face to face meetings and phone calls and initial follow-up. 6 CG withdrew because of the demise of the patient. Online forum was attended by only 2 CG. |
| Lim et al., 2014 | Efficacy of Relaxation Intervention for TKR (Total Knee Replacement) surgery patients | To reduce pain and stress, and improve self-efficacy and reduce anxiety in TKR surgery patients | Audiotaped relaxation instruction, soothing background music | The intervention consisted of 3 daily 1-hour individual sessions combining theory and practice. Theory covered postoperative pain-tension effects, relaxation benefits, and daily implementation strategies. Practice included guided breathing exercises and imagery using 20 minutes .audio recordings with background music while patients lay comfortably in bed. Sessions ended with positive reinforcement and encouragement for regular practice. | NR | Face to face | Surgical ward | 3 daily 1-hour individual sessions postoperatively | Tailored for specific patients with mobility limitation | NR | Validated scales were used to measure the outcomes. Objective assessments of stress complimented the subjective measures. English and Chinese versions of the questionnaires were used. Content validity assessed by experts. | Moderate adherence was noted with 18 out of 30 eligible participants completed the study. 12 declined participation. Intention to Treat (ITT) analysis was performed to tackle missing data. Measurements taken under same conditions for consistency. Qualitative feedback on ease of use collected. |
| Lim et al., 2019 | Support availability, Thinking positively with acceptance, Overcoming social stigma, Minimising negative feelings, Analysing self‐efficacy in stoma care (STOMA) psychosocial intervention programme | To address and promote stoma self-care efficacy, acceptance, psychological wellbeing and QoL in people with colorectal cancer. The theoretical framework relies on self-efficacy theory and stoma acceptance framework. | Educational booklet on stoma care and coping | The STOMA programme included individual face to face psychoeducation sessions done preoperatively and 5 telephone follow-up sessions conducted preoperatively (n=1), postoperatively (n=4). The contents were introduction to psychosocial interventions, STOMA programme outline, community resources and support services, common issues with postoperative stoma, step-by-step stoma care training, and stoma education protocol for goal setting. | Trained nurse | Face to face and tele follow-up | Inpatient ward | Intervention was delivered pre‐operatively, during admission and 5 days postoperatively | NR | NR | Experiences of the patients were captured through qualitative interviews post intervention. | The findings showed perceived benefits of the programme and positive experiences. None of the participants withdraw or refused. |
| Lim et al., 2019 | STOMA psychosocial intervention programme | The intervention was aimed to address and promote stoma self-care efficacy, acceptance, psychological wellbeing and QoL in people with colorectal cancer. The theoretical framework relies on self-efficacy theory and stoma acceptance framework. | Educational booklet on stoma care and coping | The programme integrated a multi‐modal and multidimensional approach, which included a pre‐operative individual face to face psychoeducational session, an educational booklet provided and 4 telephone follow‐ups (1 pre‐operatively and 4 postoperatively) | Nurse with experience in colorectal nursing | Face to face and tele follow-up | Inpatient ward | Intervention was delivered pre‐operatively, during admission and 5 days postoperatively | Tailored for the specific population | NR | The contents of the intervention were developed and cross‐examined by an expert panel, including an experienced stoma care nurse clinician, a colorectal surgeon, and an academic assistant professor. Anxiety, depressive symptoms and QoL were measured through validated questionnaires. | There was an improvement in acceptance of stoma in the intervention group. No significant change noted for other outcomes. Only a single participant dropped out from the study. ITT analysis was performed. |
| Mahendran et al., 2015 | Brief nurse-led psychosocial intervention programme | Cancer patients typically share their emotional concerns with nurses rather than actively seeking professional mental health support. A structured intervention early in their cancer journey is particularly beneficial, as newly diagnosed patients respond well to comprehensive programs that combine health education, stress management techniques, behavioural training, coping strategies, and psychosocial support. | Supplementary print and audio material for participants to practice the techniques at home | The programme involved psychoeducation elements such as stress management, sleep hygiene measures, recognising anxiety and depression symptoms, how to access help and included behavioural techniques (e.g., deep breathing exercises, progressive muscle relaxation, positive self-talk), counselling and supportive therapy. | Trained oncology nurses who received training from psychologists and psychiatrist and through simulation sessions. | Face to face | Medical Oncology Clinics | 20-30 minute sessions (initial 2 visits done monthly and the remaining bimonthly, total 6 months) | NR | NR | Programme was conceptualised by a multidisciplinary team (MDT) of oncologist, nurse, psychologist, and psychiatrists. Locally validated questionnaires were used to capture the outcomes (Hospital Anxiety and Depression Scale [HADS], and EuroQol QOL Visual Analogue Scale ([EQ VAS}) | 70 out of 121 eligible patients (58%) chose to participate in the study, of which 63 participants (90%) completed all four sessions and 52% completed overall follow-up. Significant improvements were noted in distress, anxiety, depression and QoL. |
| Merchant et al., 2024 | Exercise and Cognitive Stimulation Therapy (CST) | To improve intrinsic capacity in pre-frail seniors through exercise and CST. | NR | The programme included 60 minutes of group-based exercise and subsequently 30 minutes of CST, twice a week for the first 3 months. This was followed by group-based exercise only, twice a week for the next 3 months. Participants also received general health education advice. | Trained research staff, health coaches | Face to face group sessions | Community | Group-based exercise: 60 minutes per session, twice weekly over 6 months CST: 30 minutes per session, twice weekly over first 3 months | Participants were split into Mandarin and English-speaking groups for CST. | Recruitment was affected  by Covid-19 restrictions. | Participants were pre-screened for frailty. Validated scales were to measure outcomes. Data were collected at 4 timepoints: baseline, 3-months, 6-months, and 12-months. Trained research staff administered the study protocol and trained health coaches led the group exercise sessions. | High adherence was noted with 187 of 190 participants completing baseline and 3-month assessments and thus were included in the data analysis. The intervention group showed improvement in cognition and psychological function. |
| Neo et al., 2024 | Nurse-led telehealth programme | To provide regular monitoring and management of cancer patients requiring palliative care in the outpatient setting through a nurse-led telehealth intervention. | Zoom, mobile phone, phone calls, psychoeducation guide, helpline | The programme involved a first consult with a specialist palliative care physician and nurse on Zoom, followed by weekly reporting of symptoms and concerns. If concerns were flagged, nurses would contact participants via phone/video calls and develop a treatment plan. Participants were also given a psychoeducation guide and helpline number to call. | Palliative care physician, nurse, oncologist, pharmacist, social worker | Online (Zoom) | Community | Weekly monitoring of symptoms over 12 weeks, nurse contact on needs basis | Participants were triaged based on symptom severity, with customised treatment plans based on needs | None | Participants were recruited on stringent criteria, requiring advanced cancers (Stages 3 and 4) in areas without existing palliative care telehealth interventions, and a prognosis of 2 years or less. Symptoms were monitored weekly using a locally validated scale. | High adherence rate was noted with 74 of 88 participants completing the programme. Mean Integrated Palliative Outcome Scale (IPOS) scores decreased from pre- to post-intervention. |
| Ramazanu et al., 2021 | 3H (Head, Heart, Hands) intervention | To evaluate the 3H intervention and explore the experiences of couple taking part in post stroke 3H interventions. The intervention focused on information support, shared decision making and practical skills necessary for coping. | .audio-recorders and interview guides | Participants and CG underwent 6 sessions over a period of 3 weeks, consisting 4 face to face or group sessions and 2 dyadic sessions. The sessions focused on how stroke affected their marriage, shared decision making on social support and rebuilding marriage, balancing roles and support for CG. An interview was conducted which lasted for 15-50 minutes, which was .audio-recorded and transcribed verbatim. Content analysis was performed. | Registered stroke nurse, interviews conducted by the first and second authors (role NR). | Face to face individual or group sessions | Rehabilitation hospital | 6 sessions (timing NR) and a follow-up interview (timing NR) that lasted for 15-50 minutes. | Intervention was tailored to the duration of patients' stay in the hospital. | NR | 3H interventions were planned by trained nurses and tailored to the length of stay. In-depth interviews , .audio recordings and verbatim transcription was planned. The interview was only conducted in English. | All procedures conducted as planned by trained team members and analysis was conducted as planned. All participants completed the sessions and interviews. The intervention improved their coping skills. |
| Tan et al., 2009 | Group CBT programme | To improve functioning and wellbeing in patients with chronic pain. The theoretical framework relies on CBT. | NR | The programme involves a 6-9 day group CBT to help patients with chronic pain manage their pain effectively. Components of the programme include teaching patients cognitive-behavioural methods to cope with pain, including goal-setting, pacing, cognitive restructuring, distraction, problem solving, sleep hygiene, communication skills, maintaining changes and relaxation techniques among others. | Anaesthesiologists, psychologists, physiotherapists, nurse clinicians | Face to face group sessions | Hospital | 6-9 days over 2 weeks | Programme was adapted from the Australian pain management programme ADAPT but shortened to suit the local population that had difficulty committing to long programme durations. | None | Participants were recruited on stringent inclusion and exclusion criteria. Validated scales were used to measure outcomes at pre- and post-intervention and at 1 month and 6 months follow-up. | The group CBT programme was effective in reducing the use of unhelpful strategies, fear of harm, pathophysiological beliefs, depression, anxiety, and stress. It also improved self-efficacy and self-statements. High adherence was noted with 100% of participants completing the intervention. |
| Tan et al., 2024 | OPTIMUM—Optimising care of Patients via Telemedicine In Monitoring and Augmenting their control of Diabetes Mellitus | To improve self-efficacy in adults with type-2 diabetes mellitus (T2DM) through a telemonitoring, tele support and tele education intervention. The theoretical framework relies on the health belief model. | OPTIMUM mobile app, phone calls, educational videos | The programme involved different intervention tiers depending on T2DM severity, ranging from automated messages for low risk participants, motivational messages for normal risk participants, phone calls for moderate to high risk participants, and an in-app survey with phone calls for high risk participants. Participants also received 9 mandatory and 9 optional educational videos on the OPTIMUM mobile app. | Nurse, self-directed | Mobile app (self-directed), phone calls, text messages | Primary Care | 6 months of weekly monitoring, nurse contact on needs basis | Mode of contact was dependent on level of risk. | None | Participants were recruited on stringent criteria, defining poor controlled T2DM based on clinical parameters. An interview guide was provided to the interviewers to ensure consistency in data collection. Data were collected from participants until data saturation was reached. Interviews were audio-recorded and transcribed by a professional and later audited for accuracy. | All 21 participants completed the semi-structured interviews. Enhanced self-efficacy was reported. |
| Teo et al., 2020 | Cognitive Behavioural Therapy with Mindfulness and Values-based activity (CBT-MV) | To improve symptom management in patients with advanced breast cancer. The theoretical framework relies on CBT and Acceptance and Commitment Therapy (ACT). | .audio recordings, handouts | The programme involved 4 hourly sessions over 8 weeks. Session 1 focused on psychoeducation, session 2 focused on activity pacing to manage pain and values-guided activity planning, session 3 focused on identifying and coping with unhelpful thinking patterns, and session 4 involved a review the skills learnt and planning ahead for potential challenges. | Psychologists with experience working in oncology settings | Face to face individual sessions | Hospital | 4 hourly sessions, delivered within 8 weeks | None | None | Validated scales were used to measure outcomes at pre- and post-intervention and at 3 months follow-up. A manualised intervention protocol was used. Psychologists delivering the intervention had experience working in oncology settings and received training from the study Principal Investigators (PI). | For the Singapore sample, small effect sizes were observed for anxiety and depression and high levels of engagement were observed. High adherence was noted with 85% of participants completing the intervention. |
| Teo et al., 2020 | CBT-based intervention | To improve self-efficacy in patients with advanced colorectal cancer. The theoretical framework relies on CBT. | Information sheets, manualised therapist guide, writing exercises, reading materials | The programme involved 4 hourly sessions over 8 weeks. Session 1 focused on psychoeducation, session 2 behavioural skills training for symptom management, session 3 focused on cognitive strategies to manage worries, and session 4 involved strategies to enhance social support and changed dynamics within the family. | Psychologists trained in CBT | Face to face individual sessions | Hospital | 4 hourly sessions, delivered within 8 weeks | Intervention adapted for local use e.g. materials, exercises, examples used | None | Participants were recruited on stringent inclusion and exclusion criteria. Validated scales were used to measure outcomes at baseline, 8 weeks, and 16 weeks. A manualised therapist guide was used. Psychologists delivering the intervention were trained in CBT and 10% of audio recordings were checked. | The intervention was effective in increasing self-efficacy in patients. High adherence was noted with 88% of participants completing the intervention. |
| Teo et al., 2024 | Renewing Intimacy and SExuality Intervention (RISE) | To address marital and sexual distress in female breast and gynaecologic cancer survivors through a psychological-based intervention involving psychosexual education and behavioural skills training. | Video conferencing platform, printed summaries, vaginal moisturiser, personal lubricant, intervention guide for therapists | The programme included psychoeducation about relationship and sexual changes occurring along with cancer treatment, couple communication strategies, sexual response cycle, identifying and relaying intimacy and sexual needs, and cognitive strategies. Activities were also assigned as needed. | Couples-work trained therapists | Face to face sessions, online (video call) sessions during Covid-19 | Hospital | 3 2-hour sessions over 6 weeks | Appointments were flexibly scheduled. Suggested exercises were not mandatory. Participants were given the option to bring their romantic partners into the sessions. | Due to Covid-19 restrictions, the option to receive the intervention virtually through video conferencing was offered. | An intervention guide was developed to guide the implementation. Facilitators were allied health professionals trained in couples work. A waitlist control group was included to reduce confounds. Validated scales were used to measure outcomes. Data were collected pre- and post-intervention. | High adherence was noted with 88% of participants completing the programme. Improvements in marital satisfaction, sexual satisfaction, sexual dysfunction, and body image were observed. |
| Wang et al., 2015 | Comprehensive Psychological Intervention | To improve psychological outcomes in patients with refractory functional gastrointestinal disorders (FGID) through a psychological intervention programme. | Information handout | The programme included an interview by a trained coordinator, usually within the first day of referral. During the interview, the coordinator educated participants on FGIDs before psychological screening. Those who met criteria were offered an appointment to consult a psychiatrist for assessment and treatment (medication and therapy) Participants who declined received further psychoeducation on stress management, including demonstrations of relaxation techniques and countering the validity of stressful thoughts. All participants were encouraged to join a monthly support group facilitated by a psychologist and trained coordinator, involving peer sharing and talks by FGID specialists. | Psychiatrist, psychologist, coordinator, dietician, gastroenterologist | Face to face | Hospital | Total duration: 6 months Interview with coordinator: 30-60 minutes  Support group: 1 1.5-hour session monthly | Support group participation was encouraged but not compulsory. Participants were given the option to consult a psychiatrist or receive psychoeducation from the coordinator. | For participants who could not be screened physically within the first 24 hours of referral, phone calls were arranged. | Participants were screened by trained coordinators. Validated scales were used to measure outcomes. Data were collected at baseline and 3 months follow-up. | Moderate adherence of 76.2% completing at least 3 months of follow-up was reported. Improvements in quality of life and anxiety scores were observed over time. |
| Wang et al., 2018 | Coronary Heart Disease Self‐management Programme (CHDSMP) | To empower patients with coronary heart disease (CHD) to manage their condition at home, thereby improving health-related quality of life (HrQOL) and psychological wellbeing. | Booklet, DVD, telephone follow-up interview script, phone calls | The programme involved an educational booklet on CHD, a video disc containing educational content (e.g., relaxation techniques, physical exercises, coping strategies), an individual 30-40-minute in-person education session, and weekly follow-up phone calls. | Research Nurse | Face to face, self-directed (booklet and DVD), follow-up phone calls | Participant's home | 4 weeks, 1 30-40 minute education session, weekly follow-up phone calls | Programme and materials were developed in consideration of local context. | None | Sample size was calculated by considering effect size. Block randomisation was done. Validated scales were used to measure outcomes at pre- and post-intervention, and at 16 weeks. ITT analysis was done. An interview script was developed for the follow-up phone calls. | The programme had no effect in improving psychological status and HRQOL. High adherence was noted with 84% completing the programme. |
| Wong et al., 2021 | Music Therapy | To improve functional outcomes in children undergoing cancer treatment through a music therapy intervention. | Guitar, keyboard, percussion instruments, digital devices (e.g., phones, laptops, tablets), recorded music | The programme involved initial rapport building between a music therapist and the child. Depending on the child's musical preferences, a suitable music therapy intervention would be recommended. These interventions can be receptive, recreative, improvisational, or compositional. The child would also be given emotional freedom to verbally express their thoughts and emotions as elicited by the music. | Music therapist | Face to face | Hospital | 1-3 sessions per week, up to 1 year | Frequency of sessions depended on treatment schedule and agreement between the music therapist and the child and family. while type of intervention depended on child's musical preferences | None | Validated scales were used to measure outcomes. Data were collected every 3 months up to 1 year. | No participant withdrawal from music therapy was reported. 89.2% of these children achieved their therapeutic goals. |
| Woo et al., 2022 | Nurse-led Integrated Chronic care E-enhanced Atrial Fibrillation (NICE-AF) | To study the effectiveness of NICE-AF in improving patient reported and clinical outcomes which could lead to adoption of this to a large scale permanent service for the needy. The programme followed elements from chronic care management with a shift to integrate care model from physician centric care at hospital. | Electronic decision support flow sheet, online materials for patient education | The NICE-AF clinic was led by an APN who collaborated with a cardiologist and family physician (FP) to deliver the care. The baseline activities included AF screening. follow-up was done at 3 and 6 months. The APN used electronic decision support flow sheet for taking clinical decisions. The APN also delivered regular education through websites in multiple languages which covered lifestyle and health related topics. Participants received fast track appointments and teleconsultations with cardiologists when needed. Validated measures were used to assess outcomes at 2 time points: at first consultation and 6 months after the first visit. Clinical outcomes were captured 6 months before and after the study entry from medical records. | APN, FP, cardiologist | Face to face consultations, online education, tele support with cardiologist | Polyclinic | Baseline consultation was followed by 3- and 6-months visits at the clinic. If there was any change to the management plan, the follow-up visits would be 3 months and 6 months post change. | NR | Recruitment was suspended due to the Covid-19 pandemic for 8 months which led to sample size being reduced to 43 from 170. | Systematically arranged study procedures, patient support, and follow-up. Trained APN who collaborated with other clinicians to improve care. Self-reported outcomes were captured through validated measures and clinical outcomes through medical records. Pre- and post-measurements. No comparison group was present. | Only 43 completed the programme instead of proposed 170 due to pandemic-induced disruption. Data were collected and analysed as planned at pre-post time points. Improvements in depressive symptoms were noted. |
| Yang et al., 2017 | iACT-CEL | To improve mental health outcomes in people with chronic pain through an internet-based ACT intervention. The theoretical framework relies on the psychological flexibility model. | Online programme | The programme involved 2 face to face sessions with a therapist and 6 self-directed online sessions of ACT. In week 0, participants completed baseline questionnaires and scheduled the first face-to-face session with the therapist the following week. In week 1, the session was used to guide participants on how to use the online programme. Online sessions were then completed twice weekly for the next 3 weeks, covering core components of ACT, and involving a combination of online written exercises, experiential exercises, and metaphors. The final face to face session with the therapist was scheduled for week 5. | Self-directed (online programme), therapist | Face to face, online | Community | Face to face sessions: 2 45-minute sessions for start and end of programme Online: 2 45-minute sessions per week for 3 weeks | Tailored to the specific population | None | Participants were recruited on stringent inclusion and exclusion criteria, and were screened for eligibility using a predesigned checklist and referencing past medical records. Validated scales were used to measure outcomes. Data were collected at 3 time points: baseline, post-intervention, and 3 months follow-up. ITT analysis was done to control for missing data. | High adherence was noted with 90.9% completing the intervention. Significant improvement in depression was observed. |
| Zhang et al., 2025 | Cancer Prehabilitation Exercise Diary | To guide patients in their daily exercises through the use of instructional videos and improve adherence to exercise prescriptions. | Cancer Prehabilitation Exercise Diary within the Health Buddy mobile app, which includes instructional exercise videos | The intervention involved a cancer prehabilitation exercise diary embedded in the Health Buddy mobile app, and includes instructional exercise videos to guide participants with their daily exercises. | Psychiatrists, nurses, AHP | Mobile app (self-directed) | Home | Daily, 20 days on average | Exercises and reminders were customised for each patient | None | A standardised video library was provided to participants. Validated scales were used to measure mental health outcomes. Data were collected pre- and post-intervention. | High adherence of 80% was observed. No significant outcomes on anxiety and depression symptoms were observed. |
