# Supplementary material for: Mapping programmes for mental health promotion in Singapore: A scoping review
Source: PLoS One. 2026 Apr 28;21(4):e0347518. doi: 10.1371/journal.pone.0347518 (PMC13124008; doi:10.1371/journal.pone.0347518)
Supplement: S6 Table — (DOCX) [file pone.0347518.s006.docx]

**S6 Table:** **Study characteristics based on TIDieR checklist list for those targeting mental health outcomes in those with neurodevelopmental and learning conditions**

| **Author** | **Name of intervention** | **Rationale/goal of elements essential to the intervention** | **Materials used in the intervention** | **Procedures** | **Provider's details** | **Mode of delivery** | **Location** | **Timing and dose** | **Tailoring** | **Modifications** | | **Fidelity** | **Actual adherence** |
| --- | --- | --- | --- | --- | --- | --- | --- | --- | --- | --- | --- | --- | --- |
| Bernard-Opitz et al., 2004 | Behavioural Interventions vs Natural Play Interventions | To assess and compare the benefits (attention, compliance, and communication) of behavioural and natural play interventions on children with autism spectrum disorder (ASD). | Teaching and play materials including toys, objects, pictures etc. | For play conditions the trainers followed aspects such as levels of play, types of interaction games and toy, enhancing imitation and communication during play and experienced based learning and role play was used to demonstrate natural language paradigm. Praise and natural reinforcers were used. Differences between the intervention are not demarcated well. Pre-post assessments were conducted by co-ordinators and a naive observer which were videorecorded and a cross over design was adopted. Parental feedback was collected. A tele follow-up was conducted after 4 months. | Psychology honours graduate with 2 years’ experience together with a psychology graduate conducted behavioural condition. Play condition was delivered by a psychology graduate with 6 months experience. | Face to face individual sessions | Home and Behavioural Intervention Centre | Children received 6 hours training for each condition which lasted for 5 weeks totalling 30 hours per child. Parents provided 10 additional hours. | Trainers set individualised intervention gals with caregivers (CG), comparison groups were based on variables such as chronological age and parent education. | NR | The sessions were supervised by trained psychologists who gave timely feedback to the trainers for quality control. Sessions were also conducted by trained staff/student volunteers and witnessed by a naïve coordinator and were videotaped. Feedback was collected form participants and parents. | | Only 8 children and parents were involved in the study who completed all the procedures. Interrater reliability was calculated to be 75.1% for compliance, 83.9% for attending behaviour and 86.9% for communications. Improvements in symptoms were noted. |
| Fitrya, 2021 | English Main Literacy Programme (MLP) | To investigate if MLP can improve the reading and writing skills in dyslexic students. The intervention was developed based on evidence-based research, instructions from National Reading Panel (US), Professional Practice Guidelines (SG) and the Rose Report (UK). The programme provides individualised group lessons taught in accordance with the Orton-Gillingham (OG) principles. | Curriculum-Based Assessment (CBA) involving writing, reading, and spelling tests | Student were randomised into the programme where they attended the programme in small groups of 3-4 students for 2 hours weekly, from 2016 to 2018. CBA assessments were done at Term 2 and term 4 annually with a period of 6 months interval between tests. | Dyslexia Association of Singapore (DAS) educators | Face to face group sessions | DAS | 2-hour sessions per week over 6 terms (2016-2018) | NR | NR | Evidence based method delivered and assessed systematically. Large sample size. Validated measures conducted by trained educators. | | Regular attendance. Dropout rates was not indicated. Study procedures was delivered and assessed as planned which demonstrated improvements in reading and writing scores. |
| Fitrya, 2022 | English Main Literacy Programme (MLP) | To improve reading, spelling, and writing skills in students with Dyslexia. The theoretical framework relies on the OG principles, and the PPP (Presentation, Practice, Production) stages approach. | Educational digital tools, CBA | The programme included personalised group-based lessons supported digital tools. The programme starts with increasing vocabulary, followed by phonemic awareness and phonics, reading fluency, reading comprehension, and finally writing. | Dyslexia Association of Singapore (DAS) educators | Face to face group sessions | DAS | NR | Tailored for the specific population | None | Participants were recruited on stringent criteria, requiring an official diagnosis of Dyslexia based on IQ and low literacy resulting in poorer academic performance. Separate tests were administered to assess reading, writing, and spelling components. Scores were collected pre- and post-intervention. | | There was a statistically significant improvement in mean percentage scores post-intervention. No participant withdrawal was reported. |
| Lim et al., 2012 | Brain-computer interface (BCI) attention training programme | To investigate if BCI can improve attention-deficit/hyperactivity disorder (ADHD)symptoms in children, clinical predictor of response and electroencephalography (EEG) changes as a function of response. | BCI game system includes head band with Bluetooth connected dry EEG sensors, CogoLand game software and English and Mathematic worksheets | The programme an included intensive phase of 3 training sessions weekly for 8 weeks followed by a booster session monthly for 3 months. Mathematics and English assessments were done from second session which took 10 minutes. EEG was monitored during the assessment which was monitored by therapists. Assessments done at baseline, week 8, 20 and 24. | 3 therapists with graduate degrees in psychology, who were trained in headband fitting and standard treatment protocol. | Face to face, computer based individual sessions | Clinical setting | Intensive phase: 3 training sessions weekly for 8 weeks . Booster Phase: Monthly sessions for 3 months. | More intensive BCI sessions in unmedicated children | NR | Procedures administered by trained clinicians. Objective measures through regular EEG monitoring and validated assessment tools. Outcome measured academically. | | Out of 20 children enrolled 85% completed. Procedures completed as planned with improvements in symptoms achieved. |
| Ooi et al., 2008 | Cognitive Behavioural Therapy | To investigate the efficacy of a manualised group treatment programme for high functioning children with ASD in Singapore. The study aimed to achieve a significant reduction in anxiety for children and stress for parents and teachers. | Validated questionnaires, visual cues, and social stories. | The programme had 16 sessions, each lasting 90 minutes and conducted in groups of 3. Sessions 1-3 included understanding the feelings and emotions of the child and lessons for them to handle anxiety, sessions 4-8 focus on techniques to manage anxiety which included relaxation techniques such as breathing exercise and positive thoughts. Sessions 9-15 focused on problem-solving strategies based on STAR procedure (Stop, think act and reflect). | Two therapists, who hold postgraduate degrees in psychology and are experienced with working with children with ASD. They were assisted by collaborators. | Face to face group sessions | Special education school in Singapore | 90-minute session per week over 16 weeks | The CBT was adapted for anxiety in children with ASD children. Additional tailoring included sessions in small groups, visual cues, and social stories. | NR | Validated questionnaires were used and an adapted version of a well-accepted form of therapy was employed. Trained staff members supported by collaborators conducted the sessions. Data was collected pre and post session. Sample size was too small. | | Dropout rate was not reported. Study was conducted and data was analysed as planned. Improvements in anxiety symptoms and stress levels were achieved. |
| Tan et al., 2022 | Lexicaid | To improve reading, learning difficulties, reading motivation and self-perception in students with dyslexia. | Lexicaid mobile application, phones, headphones, charging unit, weekly log | The Lexicaid application involved 2 modules. The word scanning module helped students identify words by scanning texts, while the flashcard module helped to reinforce word learning through repetition. Students were instructed to access the app on the assigned mobile phones for reading activities or while doing homework for at least 2.5 hours a week, and submit a weekly log for monitoring of usage and reading activity. | Self-directed | Self-directed | Participant's home | 2.5 hours a week, minimum 6 weeks | Customisable features such as audio output, font display, flashcard function, and text-to-speech options | Lexicaid was expected to be used during school hours or intervention sessions, but was converted to home-based due to Covid-19 restrictions. | The app was developed in conjunction with inputs from dyslexia experts. Validated scales were used to measure outcomes at pre- and post-intervention for both experimental and control groups. | | The programme was effective in improving perceived writing and reading competence. Moderate adherence was noted with 68% retention in the study. |
| Tan et al., 2024 | Immersive Interactive Mixed Reality educational intervention | To improve mental wellbeing, academic and social skills in children with cognitive disabilities through the use of immersive interactive mixed reality in the teaching curriculum. | Projectors, interactive whiteboard, 3D tracking camera, templates and default scenes, proprietary authoring tool | The programme involved 30-minute immersive interactive mixed reality technology sessions conducted once a week in school, with educational content focusing on life skills, numerical skills, and literacy/verbal skills. Most schools assigned students into groups of 5 to 6 to maximise learning. | Teachers trained in using the software and content development | Face to face group sessions | School | 30-minute session per week over 4-8 months | Content was customised by the teachers | None | A validated scale was used to measure outcomes on well-being. Data were collected at 3 timepoints: baseline, interim, and post-intervention. Qualitative data were also collected from teachers. Teachers received 2 training sessions on content development and software usage. | | No participant withdrawal was reported. Significant improvements in mental wellbeing, social skills, teachers' work satisfaction and teachers' sense of efficacy were observed. |
| Yap et al., 2019 | Signposts for Building Better Behaviour programme | To help parents and CG learn strategies to avoid and manage difficult behaviour in their children with developmental disabilities. | NR | The programme involved sessions focused on behavioural management strategies such as measurement of child's behaviour, setting goals for the difficult behaviour, triggers and consequences of behaviour, and behavioural reinforcement. | Psychologists, OT and speech-language therapists, early childhood teachers, professionals employed by hospital department, non-profit organisation related to child and family service | Face to face group sessions (parents and child) | Hospital | 2-2.5-hour sessions per week over 5 weeks | None | None | Validated scales were used to measure outcomes at pre- and post-intervention and at 3 months follow-up. | | The programme was effective in increasing parenting efficacy. High adherence was noted with 85.9% attending at least 4 of 5 sessions. |
