# Supplementary material for: Mapping programmes for mental health promotion in Singapore: A scoping review
Source: PLoS One. 2026 Apr 28;21(4):e0347518. doi: 10.1371/journal.pone.0347518 (PMC13124008; doi:10.1371/journal.pone.0347518)
Supplement: S7 Table — (DOCX) [file pone.0347518.s007.docx]

**S7 Table:** **Study characteristics based on TIDieR checklist list for those targeting mental health outcomes in healthy population**

| **Author** | **Name of intervention** | **Rationale/goal of elements essential to the intervention** | **Materials used in the intervention** | **Procedures** | **Provider's details** | **Mode of delivery** | **Location** | **Timing and dose** | **Tailoring** | **Modifications** | **Fidelity** | **Actual adherence** |
| --- | --- | --- | --- | --- | --- | --- | --- | --- | --- | --- | --- | --- |
| Ang, 2018 | Hospital-based Suicide Prevention Programme | To reduce the high rates of suicide in the inpatient setting. | Learning video with post-video Multiple Choice Questions (MCQ) to assess learning | The programme involved training new and existing staff with knowledge and skills in recognising and preventing and managing suicide attempts. This also included reviewing hospital policies to prevent suicides and environmental modification to remove hazards such as installation of grills in windows and lobbies. Lectures and video guidance given to staffs in high-risk wards, medical officers, and all house staff. | Department of Psychological Medicine at Tan Tock Seng Hospital | Face to face lectures, e-learning modules, and environmental modifications | Hospital | Implemented in 2005 and ongoing | Tailored for the hospitals environmental, manpower and inpatient setting. Special training for new appointees and those in high-risk wards. | Modifications include introduction of e-learning module to reach a wider audience and an MCQ to assess learning, refurbishment of medical psychiatry care unit for close monitoring of high-risk patients. Initial attention focused on high-risk areas which was modified to include all house officers, medical officers and as well as other regular staffs both new and existing. | Multipronged approach planned to include training, policy changes and structural changes to reduce suicides. Evaluation of training outcomes through MCQ. Mandatory sentinel event reporting. | Tracked suicide rates through mandatory reporting which showed a drop from 12 suicides to 7 during the period. |
| Bos et al., 2018 | Neonatal Discharge Programme | To enhance parental efficacy and reduce psychological distress among parents of very low-birth-weight infants. The theoretical framework relies on Bandura's self-efficacy theory. | follow-up phone calls and home visits | The programme included a half-day (4 hours) face to face teaching session covering topics like home feeding, infant care, and basic life support. | Dieticians, speech therapists, physiotherapists (PT), and nurses. | Face to face teaching and demonstrations. | Hospital | NR | Parents were given the option to choose the time for their session. Post-discharge questionnaires were administered by phone call or home visit depending on the participants' preference. | None | Validated scales used for outcome measures. Data were collected pre- and post-intervention (2 weeks post-discharge). | Improved parental efficacy and reduced psychological distress post-intervention. |
| Chan et al., 2015 | Driver Retirement Programme | To help older taxi drivers navigate the challenges of transitioning out of their driving career and maintain meaningful engagement in daily activities after retirement. The programme was based on the UQDRIVE framework, which includes essential skills such as understanding the aging process, maintaining purposeful activities, managing finances, developing fulfilling routines, building social support networks, and staying actively engaged in community life. | Booklet and slide format in English and Chinese versions | Participants (73 years and above) were recruited through hospitals or self-referral by researchers. The programme was delivered through a combination of individual (1.5 hrs per session) and group sessions (3 hours), over 4-7 weeks. Each participant first underwent collaborative planning with a health professional to identify personal needs and goals that focused on personalised support and goal-setting, while group sessions with 2-7 participants facilitated peer support and shared learning experiences. Data collection was done at baseline, post intervention and at 3 months. | Occupational therapist (OT) | Face to face, individual and group sessions | Participants' residence or community location | Conducted weekly for 4-6 weeks. Each individual session lasted for 1.5 hrs and group sessions for 3 hrs. | The components of the programme, delivery, timing, and outcome measures were tailored to the local needs. Translations were done to include Chinese speaking population. | NR | The participants were screened using the Abbreviated Mental Test (AMT) to ensure cognitive capacity. Those at the retirement age were screened and recruited. Standardised individual and group sessions across all participants. Validated measures which were translated in Chinese were used to capture the outcome. Inconvenience fee was given to minimise attrition. | A total of 18 drivers were recruited of which 15 completed the programme. Outcome measures were all collected as planned and participant feedback collected. Programme met the intended outcomes such as improved mood, self-efficacy, and satisfaction with goals. |
| Chong et al., 2024 | Get Well, Live Well programme | To promote health literacy and modify health behaviours, and improve wellbeing among community-dwelling seniors. | Educational materials, Biopsychosocial (BPS) risk screener | The programme included biopsychosocial risk screening to assess needs. Based on needs, Care Connectors provided personalised preventive health education, coordinated care services, facilitated social prescriptions, and provided social and emotional support. | Care Connectors, social worker, general practitioners (GP), allied health professionals (AHP), doctors, community, and social partners | Face to face | Community | 12 months | Interventions were personalised based on needs identified during the BPS risk screening. | Telephone interviews were conducted due to Covid-19 restrictions. | Care Connectors were trained by a team of medical and allied health professionals and received ongoing supervision and support. Preliminary data analysis was performed to guide subsequent data collection and the refinement of the interview guide. Data collection was executed until data saturation was reached. Thematic analysis was performed by three evaluators fluent in Mandarin and formally trained in qualitative research methodology. Follow-up calls were done if further clarification was required. | All participants had been participating in the programme for 12 months. Participants reported that the programme offered emotional support and enhanced social interaction. |
| Gan et al., 2021 | Family Functional Therapy (FFT) | To improve wellbeing and family functioning in youth offenders. The theoretical framework relies on the FFT clinical model which is derived from systems therapy (ST) and cognitive-behavioural therapy (CBT) | NR | The programme included FFT sessions as the first programme in addition to standard probation services. | Psychologists and social workers with experience in working with youths and trained in FFT. | Face to face group sessions | Community | 12 sessions (average) | NR | None | The team of FFT therapists had relevant professional qualifications, experience working with youths, and training in FFT. 100% were on target for dissemination adherence and fidelity. Validated scales were used to measure outcomes. Data were collected for intervention and control groups at three timepoints, pre-programme, post-programme, and at the end of probation. | High adherence was noted with 51 of 61 participants completing the programme. ITT analyses were done to account for dropouts and non-compliance. |
| Keng et al., 2022 | Headspace Mobile App Mindfulness Practice | To reduce psychological distress and improve wellbeing and working memory in healthcare workers during COVID-19 through the use of mindfulness practices. | Headspace mindfulness app, smartphones, guided meditation audio. | The programme involved the use of Headspace, a mindfulness-based mobile app. Participants first completed an introductory course on the app comprising of daily 10-minute practices for 10 days. At the end of 10 days, a 2-week follow-up call was arranged to address any concerns with mindfulness practice. For the remaining 11 days, participants continued with their preferred mindfulness practices in the app for 10 minutes daily. | Self-guided | Mobile app (self-directed) | Community | 10 minutes daily for 3 weeks, optional continued use thereafter | None | None | Usage tracked via app: pre, post, and 1-month follow-up assessments. | High adherence was noted with 79 of 80 participants completing the study. The intervention improved fear of COVID-19, compassion satisfaction, sleep quality, trait mindfulness, self-compassion, and forward digit-span task performance. |
| Kit et al., 2019 | Live Chat Online Counselling Portal | To help children address social or emotional problems through online counselling, delivered synchronously by trainee counsellors. | Live chat counselling platform | The intervention included single-session online counselling delivered through a live chat function (Ask iZ Master) within the iZ Hero Challenge portal. The intervention was delivered by trainee counsellors for children identified by teachers to be facing social or emotional problems. Most children were allocated time to have their online counselling sessions in school after school hours. | Trainee counsellors trained in online counselling techniques | Online (live chat) | Community | 1 session | 6 children received a second counselling session upon request. | Children were allocated time in school after school hours to attend their online counselling sessions, as most had indicated preference to do so due to limited access to computers at home. | Junior researcher presented her views first in all discussions to minimise the impact of power differences. Transcripts were independently coded and later cross-checked for consensus. Researchers who conducted the interviews audited the transcripts. | All children attended the online counselling session but only 23 of 33 children attended the semi-structured interview. Children found counselling helpful and solution focused. There was positive feedback across emotional/practical needs. |
| Lee at al., 2013 | Brain-Computer Interface (BCI) System | To improve attention and memory in healthy older adults through a brain-computer interface based cognitive training system. | Computers, BCI system | The programme involved 24 30-minute sessions of brain-computer interface based cognitive training over 8 weeks. In each session, participants played a virtual card-pairing memory game. | Clinicians | Virtual | NR | 3 30-minute sessions a week over 8 weeks | None | 2 participants were found to be ineligible after randomisation and were removed from the intervention. | Participants were recruited on stringent inclusion and exclusion criteria. Participants were randomly allocated to intervention and waitlist control group, stratified by education level. Validated scales were used to measure outcomes. Data were collected at baseline, post-intervention, and 8 weeks follow-up, with different versions of the scale administered to counter practice effects. Safety and Intention to Treat (ITT) analyses were done. | High adherence of 93.9% was noted. Significant improvements in memory, visuospatial/constructional, and attention were observed. |
| Lee at al., 2015 | Brain-Computer Interface (BCI) System | To improve attention and memory in healthy Chinese-speaking older adults through a brain-computer interface based cognitive training system. | Computers, BCI system | The programme involved 24 30-minute sessions of brain-computer interface based cognitive training over 8 weeks. In each session, participants played a virtual card-pairing memory game. | Clinicians | Virtual | NR | 3 30-minute sessions a week over for 8 weeks | None | 3 participants were found to be ineligible after randomisation and were removed from the intervention. | Participants were recruited on stringent inclusion and exclusion criteria. Participants were randomly allocated to intervention and waitlist control group, stratified by education level. Validated scales were used to measure outcomes. Data were collected at baseline, post-intervention, and 8 weeks follow-up, with different versions of the scale administered to counter practice effects. Safety and ITT analyses were done. | High adherence of 100% was noted. Significant improvements in memory, visuospatial/constructional, and attention were observed. |
| Lee et al., 2020 | Social norm-based intervention for physical activity | To increase exercise levels among adolescents through the use of descriptive norm messages and personal identification. The theoretical framework relies on the Social Cognitive Theory. | Text messages, Fitbit Flex wireless pedometer | The programme included weekly text messages to participants on their weekly step count, together with group members' performance. In the treatment arm, group members' names are disclosed. | Researcher | Individual text messages | Community | Weekly | None | None | Participants were randomised by gender and baseline step counts. Data was collected for the intervention and control groups, pre- and post-intervention. Validated scales and equipment (Fitbit Flex) were used to measure outcomes. ITT analysis was done to account for missing step count data. | All participants completed the study. No significant differences in mental health outcomes between the Anonymous and Onymous arms were observed. |
| Leong et al., 2022 | Intergenerational Programme (IGP) at SDCC | To enhance memory, physical strength, emotional wellbeing, and intergenerational relationships among older adults through semi-structured activities with students. | Craft materials, games (e.g., bingo, ping pong), music for singing, arts and crafts supplies. | The programme included creative and social activities or performances facilitated by students. Each visit was 1.5 hours long and the programme lasted for 4 weeks. | Students from local secondary school | Face to face group sessions | Community | 1.5-hour sessions over 4 weeks | None | None | Thematic analysis used to explore older adults’ perceptions. Contact theory applied in design. | All elderly completed the programme. It was found that: 1. IGP enhances memory and strength 2. Social interaction between older adults and younger generation 3. Emotional responses to IGP (enhancing positive emotions and satisfaction, improving self-confidence and self-esteem) 4. Attitudes and perception towards the younger generation |
| Li et al., 2014 | Course based Disability awareness programme | To improve university students' attitudes towards people with intellectual disabilities (ID). The theoretical framework includes Mere Exposure Effect where repeated exposure or contact to something could potentially change one’s attitude towards it and theory of “Persuasive Communication” which suggests that new information repeatedly presented to individuals could change their attitudes. | Coursework materials and simulations | The course included knowledge about different types of disabilities, social contact and disability simulation activities. | Certified educator with multiple years of experience | Face to face | University | 2.5-hour sessions per week over 10 weeks | NR | NR | The assessments included quantitative assessment using Community Living Attitudes Scale and qualitative assessment through reflection journals. Data was collected pre- and post-intervention. | Students attended all course work. Adherence was presumed acceptable, and the intervention attained targeted outcomes. |
| Lim et al., 2024 | Educational | To improve the knowledge, attitudes, and practices of youths in physical and mental health through raising health awareness and training them in primary preventive health. | Zoom | The programme involved Zoom lecture sessions covering physical and mental health education. Participants also attended a motivational health interviewing workshop to build capabilities in modifying health behaviours and empower them to influence their communities. Participants presented their knowledge in the form of posters and videos. | Local experts on lecture topics | Online (Zoom) | Community | 3 days | None | All activities were delivered online via Zoom due to Covid-19 restrictions. | Data were collected pre- and post-intervention. Lectures were delivered by local experts on the topic and the content was validated by local content experts and doctors from the National University Health System (NUHS). | Moderate adherence was observed with 131 of 180 participants completing the pre- and post-intervention survey. Significant improvements were observed in mental health attitudes and practices. |
| Lim et al., 2024 | "Wise and Well" programme | To empower seniors to make better lifestyle decisions through a community-based health intervention targeting health knowledge, goal attainment, and lifestyle behaviours. The theoretical framework relies on Bandura's Social Cognitive Theory. | Visual reference charts | The programme involved 8 60-minute sessions on dietary practices, engagement in exercise, health-promoting lifestyle change, and stress management and time management strategies, delivered by a multidisciplinary team. The curriculum focused on skill development through experiential and visual learning. | Dieticians, PT, occupational therapists (OT) | Face to face group and individual sessions | Community | 60-minute sessions, 8 sessions over 3 months | Tailored to the culture and literacy levels of the specific population. | None | The programme was developed by a multidisciplinary team of allied health professionals and culturally adapted for older adults. Validated scales were used to measure outcomes. Data were collected pre- and post-intervention. | High adherence was noted with 464 of 484 participants completing the programme, hence being included in data analysis. Significant improvement was observed in lifestyle goal attainment. |
| Metrat-Depardon et al., 2023 | Happiness Mentoring Programme (HMP) | To foster wellbeing amongst polytechnic students using positive psychology interventions as they are relatively vulnerable compared to the other population. The theoretical framework involved positive psychology interventions (PPI) based on PERMA model that takes into account 5 different elements such as Positive Emotions, Engagement, Relationships, Meaning and Accomplishment. | 31 item survey ( interview protocol, questionnaires, PERMA profiler, etc.) | The HMP comprised 10 weekly 45-minute sessions, each session followed a consistent structure: beginning with debriefing of previous week's activities, followed by theoretical explanation of PERMA elements, and concluding with both group and individual coaching on implementing PPIs. The first 6 sessions systematically covered PERMA elements (Positive Emotions, Relationships, Engagement, Meaning, and Accomplishment) through various exercises like "Three Good Things," "Gift of Time," and "Growth Mindset Action Plan," while session 7 added altruism and empathy components. The final 3 sessions focused on reflection, sustainability planning, and program evaluation through surveys and structured interviews, with all sessions conducted in small groups of up to 8 participants to ensure individual attention. | Researcher | Face to face group and individual sessions | Tutorial room in a Singapore polytechnic | 45-minute sessions per week over 10 weeks | PERMA framework adapted to local culture. PPI implementation was flexible according to the student's needs, personal concerns and feedback was captured. | NR | Structured weekly sessions based on established framework. Parental consent obtained for minors and the study received ethics approval. Validated questionnaires were used to capture the outcome. | Study was conducted as planned and accomplished the intended outcomes. In the experimental group, 7 students dropped out and 6 dropped out from the control group. Feedback was collected from the students regarding the programme. |
| Muckle and Lasikiewicz, 2017 | Animal assisted activities (AAA) vs quiet reading | To assess the effectiveness of AAA in reducing anxiety, blood pressure, stress and self-esteem compared to quiet reading. Stressful challenges in university life can lead to psychological distress, suicides, and attrition. Animals provide acceptance and unconditional positive regard without creating a fear of rejection which reduced the feeling of helplessness and promote self-esteem, sense of safety and confidence. | Reading materials and Therapy dogs | The participants were recruited through posters in the campus and interacted with trained therapy dogs (large and small breeds, n=15) during a 1-hour session. The interaction included petting, hugging, feeding, doing tricks, walking, grooming, playing fetch, and photo taking. | Therapy Dogs Singapore | Face to face | Classrooms or lecture halls in the university | 1 hour session | Accommodated to religious and cultural beliefs, availability of small and large breed dogs and activities which are suitable/comfortable for the students. | NR | The study used different breeds of trained dogs and a comparison session for fidelity. The questionnaire used was well validated. Pre- and post-measurements were taken to track outcomes. | All procedures and measures were completed as planned for 59 students. No deviations reported. The intervention reduced anxiety and blood pressure. |
| Ng et al., 2018 | Horticultural Therapy (HT) | To improve mental wellbeing and modulate immune and endocrine biomarkers in older adults through gardening activities. | Plants, gardening tools, horticulture materials. | The programme involved 15 sessions of horticultural activities over the span of 6 months. The sessions were conducted weekly for the first 3 months, followed by monthly for the following 3 months. Each session lasted an hour. Activities ranged from park walks, cooking, gardening, and other activities. | Experienced instructors | Face to face group sessions | Community | Weekly sessions for 3 months, monthly sessions for next 3 months | None | None | Biological and psychosocial outcome measures were collected pre- and post-intervention. | High adherence was noted with no participant withdrawal. The horticultural therapy group saw a significant improvement in social connectedness. |
| Shahwan et al., 2020 | Anti-stigma intervention | To reduce stigma and improve help-seeking attitudes among university students. The theoretical framework relies on the Theory of Planned Behaviour. | Video | The programme involved (a) a lecture delivered by a mental health professional on depression and a video on the experience and recovery from depression, (b) a sharing by a person with lived experience, and (c) a Q&A session with a psychiatrist and mental health researcher. | Mental health professional, person with lived experience, consultant psychiatrist, senior mental health researcher | Face to face group session | University | 50-minute session | None | None | Validated scales were used to measure outcomes at pre- and post-intervention and at 3 months follow-up. The same intervention was delivered for all 9 sessions, with 6 of 9 facilitated by the same person. | The intervention was effective in increasing psychological openness, help seeking attitudes, and reducing self-stigma. High adherence was noted with 100% of participants completing the intervention. |
| Shorey et al., 2015 | Postnatal Psychoeducation Programme (PPP) – Process Evaluation | To explore first-time mothers’ experiences of the PPP and its impact on maternal self-efficacy, emotional wellbeing, and infant care knowledge. | Educational booklet, telephone calls | The programme included an initial 90-minute home visit conducted by a midwife, during which mothers were educated on the significance of maternal parental self-efficacy and social support, and the risks and symptoms of postnatal depression. This was followed by 3 weekly phone calls and the provision of an educational booklet to reinforce the information and provide ongoing support. | Trained midwives and nurses | Face to face home visit and tele follow-up. | Community | 1 90-minute home visit and 3 weekly telephone calls post-discharge | None | None | Qualitative thematic analysis was used for evaluation of programme acceptability. Data was collected past data saturation. Transcribed data was reviewed and coded independently and discussed for consensus. Field notes and audio recordings were used for accuracy. | The intervention improved knowledge on newborn care, self-care and breastfeeding, increased confidence levels in maternal roles, and improved help-seeking behaviour and emotional wellbeing. |
| Shorey et al., 2017 | ‘Home-but not Alone’ Mobile Health App Programme | To enhance parental self-efficacy, social support, and satisfaction with parenting using mHealth technology. The theoretical framework relies on Bandura's self-efficacy theory. | Mobile app | Participants accessed psychoeducation content and interacted with the app at their own time for 4 weeks. Reminders were sent for content engagement. | Midwives and research team | Mobile-based intervention via app. | Community | 4 weeks access post-discharge | None | None | A research protocol was published. Participants were recruited on stringent criteria including age, medical history of parents and newborns. The app was pilot tested before it was introduced to the parents. Content was developed by experts. Validated scales were used for outcome measures. Data was collected pre- and post-intervention. ITT analysis was done. | The intervention improved parental self-efficacy, social support, and parenting satisfaction scores 4 weeks postpartum. |
| Shorey et al., 2023 | Supportive Parenting App | To support new parents during the perinatal period through a theory- and evidence-based mobile app intervention. The theoretical frameworks determining the type of information and mode of delivery within the app rely on the mHealth user engagement pyramid, Bandura's Social Cognitive Theory, and Bowlby's Attachment Theory. | Supportive Parenting App, messaging platforms | The programme included parental access to the Supportive Parenting App, which contains knowledge-based content in the form of written articles, audio clips, and videos. Parents could use the app to access the content and utilise discussion forums, FAQs, and expert advice on the app to address any concerns. Parents were also matched to peer volunteers who provided emotional and informational support. | Self-directed, trained peer volunteers with lived experience of postnatal depression | Mobile app, messaging platforms | Community | NR | None | None | Content on the app was developed by a multidisciplinary team (MDT) of healthcare professionals, while peer volunteers were trained and had lived experience of postnatal depression. Sample size was calculated to consider a medium effect size and 20% attrition rate. Validated scales were used to measure mental health outcomes. Data were collected at 7 timepoints: baseline, 1 month, 2 months, 4 months, 6 months, 9 months, and 12 months postpartum. | Moderate adherence was noted with 76% completing the intervention. Significant improvement was observed in perceived social support. |
| Sia et al., 2020 | Therapeutic Horticulture Programme | To promote health and wellbeing in seniors through therapeutic horticulture. The theoretical frameworks rely on the Biophilia Hypothesis and the Attention Restoration Theory. | Plants, craft materials, gardening supplies | The programme included a combination of horticulture-based and nature art activities. The programme was group-based, starting with a series of basic horticulture sessions, followed by nature art sessions, and returning to horticulture sessions. Each session started with warm-up exercises, introduction to the activity, the activity, and a sharing session at the end. | Horticultural therapy expert, facilitators trained in conducting urban farming workshops | Face to face group sessions | Public garden | 1-hour session per week over 24 weeks | None | The Visual Analogue Scale (VAS) was implemented after the first group completed the intervention. | Validated scales were used to measure outcomes. Data were collected at 5 time points: baseline, 3 months (mid-intervention), 6 months (post-intervention), 9 months (3-months follow=up), and 12 months (6-months follow-up). | High adherence was noted with all participants completing the intervention. Significant improvements were observed in mental state, dementia scores, and depression. |
| Subramaniam et al., 2020 | Advancing Research Toward Eliminating Mental Illness Stigma (ARTEMIS) | To reduce stigma and improve depression literacy among university students. The theoretical framework relies on the stigma reduction theory. | Vignette, PowerPoint presentation | The programme involved (a) a lecture delivered by a mental health professional on depression, (b) a sharing by a person with lived experience, and (c) a Q&A session with a psychiatrist and mental health researcher. | Mental health professional, person with lived experience, consultant psychiatrist, senior mental health researcher | Face to face group session | University | 1 hour session | None | None | Validated scales were used to measure outcomes at pre- and post-intervention and at 3 months follow-up. The same intervention was delivered for all 9 sessions, with 6 of 9 facilitated by the same person. All sessions were delivered in the evenings after classes to facilitate participation.\ | The intervention was effective in improving depression literacy, personal stigma, and reducing social distance. High adherence was noted with 100% of participants completing the intervention. |
| Tan and Mankiewicz, 2024 | Brief video contact-based intervention | To reduce public stigma associated with psychosis through a psychoeducational video contact-based intervention (CBI). CBIs seek to bridge social gaps and promote meaningful relationships. The video incorporated evidence-based elements such as sharing of experiences, personal perception of their experiences, and reflecting on functional coping skills. | Video | The programme included a 90 second video of a Singaporean woman sharing her experiences with and recovery from psychosis. | Researcher | Remote | Community | 1 session, 90 second video | None | None | A validated scale was used to measure outcomes on stigma. Data were collected at 3 timepoints: baseline, post-intervention, and at 30-day follow-up. Two comparator arms were included to determine that effects were unique to the mode of delivery. a priori analysis was done to determine sample size and attention checks were done to strengthen data quality. | All participants completed the video intervention. All stigma scores except social distancing attitudes improved initially at post-intervention, before worsening over time at follow-up. Social distancing attitudes worsened post-intervention and even more at follow-up. |
| Tan et al., 2021 | Advancing Research Toward Eliminating Mental Illness Stigma (ARTEMIS) | To improve depression recognition and help-seeking preferences among university students. | Video, vignette | The programme involved (a) a lecture delivered by a mental health professional on depression and a video on the experience and recovery from depression, (b) a sharing by a person with lived experience, and (c) a Q&A session with a psychiatrist and mental health researcher. | Mental health professional, person with lived experience, consultant psychiatrist, senior mental health researcher | Face-to-face group setting | University | 50-minute session | None | None | Validated scales were used to measure outcomes at pre- and post-intervention and at 3 months follow-up. The same intervention was delivered for all 9 sessions, with 6 of 9 facilitated by the same person. | The intervention was effective in improving participants' recognition of depression, which was sustained at follow-up. The intervention also improved help-seeking beliefs, although effects were not sustained at follow-up. High adherence was noted with 100% of participants completing the intervention. |
| Tay et al., 2022 | Online HOPE Intervention | To improve help-seeking attitudes and intentions among university students through an online website intervention. The theoretical framework relies on Bandura's Self Efficacy Theory. | Website | The programme involved 4 sessions of the online HOPE intervention over 2 weeks. Each session comprised of quizzes, videos, and mental health knowledge. The first session was an introduction to depression, including self-help strategies and treatments. The second session taught strength-based, affect-based and gratitude exercises. The third session covered anxiety, while the last session was about relaxation techniques and managing unhelpful thoughts. | Self-directed (website) | Online | University | 2 sessions per week over 2 weeks | None | No face-to-face recruitment was conducted due to Covid-19 outbreak. | A waitlist control group was included to control for confounding effects of time. Validated scales were used to measure outcomes. | High adherence was noted with 99% completing the intervention. Significant improvements in depression and anxiety literacy, and personal stigma for depression were observed. |
| Tay, 2022 | Online HOPE Intervention | To improve help-seeking attitudes and intentions among university students through an online website intervention. The theoretical framework relies on Bandura's Self Efficacy Theory. | Website | The programme involved 4 sessions of the online HOPE intervention over 2 weeks. Each session comprised of quizzes, videos, and mental health knowledge. The first session was an introduction to depression, including self-help strategies and treatments. The second session taught strength-based, affect-based and gratitude exercises. The third session covered anxiety, while the last session was about relaxation techniques and managing unhelpful thoughts. | Self-directed (website) | Online | University | 2 sessions per week over 2 weeks | None | No face to face recruitment was conducted due to Covid-19 outbreak. | A waitlist control group was included to control for confounding effects of time. Qualitative feedback was sought until data saturation was reached. Interview responses were recorded and transcribed. ITT analysis was completed to account for dropouts. | High adherence was noted with 99% completing the intervention. Significant improvements in recognition of depression, help-seeking, and acknowledgement of the use of medications. |
| Wong et al., 2018 | Mindfulness-based Training | To improve sustained attention and subjective energy maintenance in nurses through a mindfulness-based intervention. The intervention was based on the mindfulness-based stress reduction (MBSR) programme. | NR | The programme involved 8 weekly 90-minute sessions of mindfulness-based training conducted by an experienced mindfulness instructor. The programme comprised of mindfulness practices, group sharing, in-class activities. Participants were also encouraged to practise the mindfulness techniques at home for at least 15 minutes each day. | Mindfulness instructor | Face to face | Hospital | 90-minute session per week over 8 weeks | None | None | Validated scales and neuroscientific tools were used to measure outcomes. Data were collected every session to observe improvements over time. | On average, participants attended 4.14 of 8 sessions. The data suggest that mindfulness training enhances the ability to sustain attention, with associated and measurable neurophysiological and subjective changes, for participants with regular attendance. |
| Wong et al., 2020 | CBT-based paraprofessional training programme | To improve depression literacy, stigma, CBT skills and help-seeking attitudes in foreign domestic workers through a CBT-based paraprofessional training programme. | Handouts | The programme involved weekly 3-hour sessions of CBT-based paraprofessional training over 4 weeks. The sessions were delivered in a group setting and addressed common issues faced by foreign migrant workers that could potentially lead to depression. The sessions involved didactics, discussions, and roleplays. Handouts and homework practices were also given to strengthen learning. | Masters' level clinical psychology trainees | Face to face | Non-Governmental Organisation office | 3-hour session per week for 4 weeks | None | Tailored to the specific population | Validated scales were used to measure outcomes. Data were collected at baseline, post-intervention, and 2 months follow-up. | High adherence of 95% completing the intervention. Significant improvements in depression literacy, CBT knowledge, and help-seeking attitudes were observed. |
| Yang et al., 2017 | Mindfulness intervention | To promote mindfulness and compassion and reduce stress and burnout in mental health professionals through a mindfulness intervention. The mindfulness intervention was adapted from the MBSR course by Jon Kabat-Zinn. | Guided meditation recordings | Participants underwent mindfulness training once a week for 6 weeks, with each session lasting 2 hours. The trainings covered psychoeducation on mindfulness and meditation practices. Participants were also tasked to practise meditation for 30 minutes daily as homework. | Certified MBSR instructor, certified Mindfulness-Based Cognitive Therapy instructors | Face to face group sessions, self-directed meditation | Hospital | 2-hour session per week for 6 weeks | Tailored to the specific population | None | Two programmes of differing durations were piloted to determine the appropriate duration for the intervention. Validated scales were used to measure mental health outcomes. Data were collected at baseline, post-intervention, and 3 months follow-up. | High adherence of 84% was noted. Improvements in levels of stress, mindfulness, and compassion were observed. |
| Yap et al., 2014 | Signposts for Building Better Behaviour Programme | To empower families to effectively address challenging behaviours of children with special needs and reduce parental stress through a family-oriented behavioural management programme. | Questionnaire booklet | The programme involved weekly group sessions facilitated by trained social workers, psychologists, therapists, and teachers over 5 weeks. The sessions involved the use of case examples to guide parents/caregivers on how to observe and manage their child's behaviour effectively. Up to 2 caregivers (CG)/parents accompanied 1 child, and up to 10 pairs attended the group session run by 2 facilitators based on a 1:5 ratio between child and facilitator. | Social workers, psychologists, therapists, and teachers trained and supervised by local Signpost trainers certified by the Parenting Research Centre | Face to face group sessions | Hospital | 1 session per week for 5 weeks | Different combinations of patients/CG were allowed. Sessions were conducted weekly unlike the fortnightly sessions in Australia (original programme). | None | Validated scales were used to measure outcomes. Data were collected at baseline, post-intervention, and 3 months follow-up. | Moderate adherence of 69% completing the post-intervention questionnaires was noted. Parents were significantly less hassled, stressed, depressed, and anxious. They were more confident and satisfied with managing their child and rated their children’s behaviours as having improved. |
| Yap et al., 2017 | Rhythm Wellness Programme | To improve quality of life (QoL), depressive mood, sleep quality and social isolation in older adults through a rhythm-centred music making intervention. | Percussion instruments | The programme involved weekly 1 hour Rhythm-centred music making (RMM) sessions for a total of 10 sessions. Instructors facilitated group drumming in a circle using various percussion instruments. Participants were encouraged to express themselves freely and interact with each other through playing their instruments. | Experienced RMM instructors | Face to face group sessions | NR | 1 hour session per week, 10 sessions | None | None | Cross over study design was used. Sample size was calculated with consideration of attrition. Validated scales were used to measure outcomes. Data were collected at baseline, 11 weeks (post-intervention for group A) and 22 weeks (post-intervention for group B). | Moderate adherence of 57.4% was noted. No significant effect on QoL was observed. |
| Yeo et al., 2016 | Preventive Test Anxiety Intervention Programme | To equip young students with coping skills to manage test anxiety through a preventive test anxiety intervention programme involving group-based CBT. | Audio CDs with relaxation script, after-school timetable, picture charts, handouts | The programme included 4 half hour CBT sessions over 4 weeks during school hours. The sessions involved components of psychoeducation, relaxation training, self-instruction, exposure to anxiety-inducing environments, and skill trainings. It was delivered in a class setting by a therapist, with homework issued at the end of each session. Students were incentivised to practise intervention skills daily and maintain self-monitoring records through rewards. | Therapist (psychologist and graduate student in an Applied Psychology programme) | Face to face group sessions | School | 30-minute session per week over 4 weeks | None | None | A control group was included to measure unique effects of CBT. Validated scales were used to measure outcomes. Data were collected at baseline, post-intervention, and at 2 months follow-up. | No participant withdrawal was reported. Significant improvement in text anxiety was observed. |
| Yeo et al., 2021 | NeeuroFIT: A community-based computerized cognitive training (CCT) | The programme was aimed at improving cognition, gait and balance in healthy older adults aged 55 years and older who are potential retirees. | Mobile apps, Electroencephalography (EEG) headset, GAITRite walkway | In this single blinded Randomised Controlled Trial (RCT), participants were randomised into the intervention and waitlist control group. While the control group continued their usual activities intervention group received game-based training for attention, memory, decision making, visuospatial and cognitive abilities using apps which were eventually paired with EEG headsets that quantified attention that indicates their performance. | Trained instructor and CCT developers | Instructor-led group-based sessions | Community Centres | 10-week programme, 2 sessions per week each lasting for approximately 2 hours | Tailored to local population (bilingual delivery), gamified to elicit interest among participants and included adaptive training to cognitively demanding levels. | Eligibility criteria (sedentary adults) was relaxed to improve recruitment rates. The class size of 9-15 attendees was relaxed to include a session with 3 participants who preferred English class instead of bilingual classes. | Multiple validated assessments that included both subjective and objective measures and single blinded RCT design that reduced the biases, planned monitoring, baseline and follow-up schedules and assessments at participant and provider levels. | All completed baseline and follow-up assessment despite some not following randomisation sequence. ITT and Per Protocol analyses were conducted and missing data was accounted through statistical methods. All assessments were done within 2 weeks of CCT. Better executive function was noted. |
| Zheng et al., 2020 | Exergames | To improve emotional wellbeing in older adults through an exergame intervention. The theoretical framework relies on social identity theory and intergroup contact theory. | Games on Microsoft Kinect | The programme involved exergame sessions twice a week for a total of 6 weeks. Participants were randomly assigned to 1 of 3 game play conditions - playing alone, playing with peers, and playing with youths. The same set of exergames were used in every session, with each game being played for 5-10 minutes. | Self-directed (games on Microsoft Kinect) | Virtual | Community | 2 sessions per week over 6 weeks | Tailored to the specific population | None | Validated scales were used to measure outcomes. Data were collected at baseline, and post-intervention. | No participant withdrawal was reported. Significant improvements in positive and negative affect were observed. |
| Zheng et al., 2022 | Web-based and home-based postnatal psychoeducational intervention | To improve maternal health outcomes in first-time mothers through a home-based or web-based postnatal psychoeducational intervention covering content on breastfeeding, breast engorgement, infant bathing, and Kegel exercises. The theoretical framework relies on Bandura's self-efficacy theory and the social support model. | Educational booklet, phone calls, website with content identical to the educational booklet and including audio clips, videos, peer discussion forums | The home-based intervention involved a 1-hour home visit by a nurse. During the home visit, the nurse provided psychoeducation through a self-developed educational booklet. 3 weekly telephone calls were also included to address any queries. | Registered Nurse, self-directed (website) | Face to face, virtual | Home | Home-based: 1 hour session, 3 weekly phone calls Web-based: 1 month access to website, 3 weekly phone calls | None | None | Validated scales were used to measure outcomes. Data were collected at baseline, 1 month, 3 months, and 6 months after childbirth. Probabilistic sensitivity analyses were adopted to address parameter uncertainty for cost and effectiveness. | No participant withdrawal was reported. The web-based intervention was most effective with the most significant improvements in self-efficacy, social support, and psychological wellbeing. |
| Zhou et al., 2017 | Positive Parenting Programme (Triple P) | To improve parenting practices and prevent recurrence of child maltreatment for parents and caregivers referred to the Child Protection Service. The theoretical framework relies on parent-child interaction theories, and risk and protective factors for child adjustment. | NR | The programme uses a tiered intervention system ranging from basic media education to intensive parenting training. Level 4 focuses on prevention and early intervention through positive parenting skills, while Level 5 provides additional emotional and cognitive training for parents at risk of child maltreatment. | Trained counsellors and psychologists certified by Triple P International | Face to face individual or group setting | Community | Weekly. Level 4: 6 individual sessions or 8 group sessions Level 5: Expedited Level 4 (2 sessions shorter than usual) + 4 additional sessions | Caregivers were assigned to either Level 4 or Level 5 depending on severity of inappropriate parenting practices and children's problem behaviours. | None | Validated scales were used to measure outcomes pre- and post-intervention. Sessions were delivered by trained counsellors and psychologists certified by Triple P International. | The programme was effective in reducing caregiver perceptions of the amount and intensity of child behaviour problems, dysfunctional parenting style, stress, depression, and increasing parenting satisfaction. High adherence was noted with 79% completing the intervention. |
